# Blood-based tumor mutational burden as a biomarker for atezolizumab in non-small cell lung cancer: the phase 2 B-F1RST trial

**DOI:** 10.1038/s41591-022-01754-x

**Published:** 2022-04-14

**Authors:** Edward S. Kim, Vamsidhar Velcheti, Tarek Mekhail, Cindy Yun, Sarah M. Shagan, Sylvia Hu, Young Kwang Chae, Ticiana A. Leal, Jonathan E. Dowell, Michaela L. Tsai, Christopher S. R. Dakhil, Philip Stella, Yanling Jin, David S. Shames, Erica Schleifman, David A. Fabrizio, See Phan, Mark A. Socinski

**Affiliations:** 1 City of Hope National Medical Center, Los Angeles, CA USA; 2 Cleveland Clinic, Cleveland, OH USA; 3 AdventHealth Cancer Institute, Orlando, FL USA; 4 Genentech, Inc., South San Francisco, CA USA; 5 Northwestern University Feinberg School of Medicine, Chicago, IL USA; 6 School of Medicine and Public Health, University of Wisconsin-Madison, Madison, WI USA; 7 Department of Medicine, Veterans Affairs North Texas Healthcare System, University of Texas Southwestern Medical Center, Dallas, TX USA; 8 Minnesota Oncology, Minneapolis Clinic, Minneapolis, MN USA; 9 Cancer Center of Kansas, Wichita, KS USA; 10 St. Joseph Mercy Hospital, Ann Arbor, MI USA; 11 F. Hoffmann-La Roche, Ltd, Mississauga ON, Canada; 12 Foundation Medicine, Inc, Cambridge, MA USA; 13 Present Address: New York University School of Medicine, New York, NY USA

**Keywords:** Predictive markers, Non-small-cell lung cancer, Diagnostic markers

## Abstract

Tumor mutational burden (TMB) in circulating tumor DNA (ctDNA) has shown promise in predicting benefit from PD-L1/PD-1 inhibitors in retrospective studies. Aiming to assess blood TMB (bTMB) prospectively, we conducted B-F1RST (NCT02848651), an open-label, phase 2 trial that evaluated bTMB as a predictive biomarker for first-line atezolizumab monotherapy in locally advanced or metastatic stage IIIB–IVB non-small cell lung cancer (*n* = 152). The co-primary endpoints were investigator-assessed objective response rate (ORR) per RECIST version 1.1 and investigator-assessed progression-free survival (PFS) between high and low bTMB subgroups at the pre-defined bTMB ≥ 16 (14.5 mutations per megabase) cutoff. Secondary endpoints included investigator-assessed PFS, overall survival (OS) and duration of response at various bTMB cutoffs, as well as safety. Investigator-assessed PFS in the bTMB ≥ 16 versus bTMB < 16 groups was not statistically significant. However, bTMB ≥ 16 was associated with higher ORR, and ORR improved as bTMB cutoffs increased. No new safety signals were seen. In exploratory analyses, patients with maximum somatic allele frequency (MSAF) < 1% had higher ORR than patients with MSAF ≥ 1%. However, further analysis showed that this effect was driven by better baseline prognostics rather than by MSAF itself. At 36.5-month follow-up, an exploratory analysis of OS found that bTMB ≥ 16 was associated with longer OS than bTMB < 16. Further study and assay optimization will be required to develop bTMB as a predictive, standalone biomarker of immunotherapy or for use in conjunction with other biomarkers.

## Abstract (web summary)

The randomized B-F1RST trial evaluating the clinical utility of blood tumor mutational burden as a predictor of benefit from atezolizumab in patients with advanced lung cancer did not meet its pre-specified primary objective, underscoring the need to further investigate the significance of bTMB as a relevant biomarker for patient selection.

## Main

Atezolizumab monotherapy is effective in the first-line treatment of patients with *EGFR*/*ALK* wild-type squamous or non-squamous locally advanced or metastatic non-small cell lung cancer (NSCLC) whose tumors have high programmed death ligand 1 (PD-L1) expression. The phase 3 IMpower110 trial (*n* = 572) included patients who had PD-L1 expression on ≥ 1% of tumor cells (TCs) or ≥ 1% of tumor-infiltrating immune cells (ICs) assessed by the SP142 immunohistochemistry assay (Ventana). Patients with the highest PD-L1 expression (on ≥ 50% of TCs or ≥ 10% of ICs) had a median OS of 20.2 months with atezolizumab monotherapy versus 13.1 months with platinum-based chemotherapy (hazard ratio (HR) = 0.59 (95% confidence interval (CI) 0.40, 0.89); *P* = 0.0106)^1^.

In addition to PD-L1, TMB may also be a useful biomarker for cancer immunotherapy benefit. tTMB, as determined by whole-exome sequencing and targeted panels, is associated with clinical benefit from multiple checkpoint inhibitors, particularly in the monotherapy setting^2–5^. TMB also appears to identify patients with NSCLC who benefit from anti-PD-L1/PD-1 treatment and patients who express low levels of PD-L1 (refs. ^1,2,6^). However, as many as 30% of patients with NSCLC may not have enough high-quality tissue biopsied at diagnosis for biomarker analyses^7^, clearly indicating a need for a non-invasive cancer immunotherapy biomarker assay. An advantage of bTMB is that the source material is readily available and is less susceptible to sampling bias due to tumor heterogeneity of biopsies obtained from single sites at a single time point^8,9^.

B-F1RST (NCT02848651) used the Foundation Medicine bTMB assay to evaluate TMB status in clinical blood samples. The assay uses a hybridization capture-based method that targets 1.1 megabases (Mb) of genomic sequence^10^. The assay can detect bTMB, provided there is adequate ctDNA, defined as an MSAF ≥ 1%. The bTMB score is expressed as the total number of single-nucleotide mutations in the genes targeted by the assay after germline and driver mutation filtering. The bTMB cutoff score of ≥ 16 (equivalent to ≈ 14.5 mutations per Mb (mut/Mb) (16 mut/1.1 Mb)) was defined in the phase 2 POPLAR training set and validated in the phase 3 OAK study in 2L + NSCLC^10^. In OAK, the HR for PFS in patients above the cutoff of ≥ 16 who were treated with atezolizumab monotherapy versus docetaxel was 0.65 (95% CI: 0.47, 0.92) compared to 0.98 (95% CI: 0.80, 1.20) for patients with bTMB < 16 (ref. ^10^).

In the IMpower110 study, exploratory analyses showed that the median OS in patients with PD-L1-positive tumors who had a bTMB score ≥ 16 (14.5 mut/Mb) was 13.9 months in the atezolizumab monotherapy arm versus 8.5 months in the chemotherapy arm (HR = 0.75, 95% CI: 0.41, 1.35) compared to an HR of 1.07 for patients with bTMB < 16 (ref. ^1^). PFS in the any-PD-L1-expression (intent-to-treat (ITT)) population who received atezolizumab versus chemotherapy was 5.7 versus 5.5 months (HR = 0.77, 95% CI: 0.63, 0.94) and, in the bTMB ≥ 16 group, was 6.8 versus 4.4 months (HR = 0.55, 95% CI: 0.33, 0.92), suggesting that bTMB ≥ 16 is predictive of improved outcomes with atezolizumab but also that it is prognostic of worse outcomes with chemotherapy.

B-F1RST was the first study to evaluate bTMB prospectively in patients with locally advanced or metastatic NSCLC treated with atezolizumab monotherapy in the first-line setting^11^. The primary efficacy objective was to evaluate response rate, and the primary biomarker objective was to assess the relationship of investigator-assessed PFS with bTMB ≥ 16. Here we report the final analysis of B-F1RST, which included OS after long-term (>3-year) follow-up, updated results for the non-biomarker-evaluable patients^12^ and additional exploratory biomarker analyses of the genomic landscape and association of the most prevalent alterations with bTMB and efficacy outcomes.

## Results

### Baseline characteristics

This study was conducted from 21 September 2016 through 14 May 2019. The final analysis included all patients with at least 18 months of follow-up at the clinical cutoff date of 26 July 2019, resulting in a median follow-up of 20.9 (range, 0.5–31.4) months. A follow-up analysis of OS (clinical cutoff date: 2 December 2020) was conducted with a median follow-up of 36.5 months.

**Extended Data Fig. 1:**
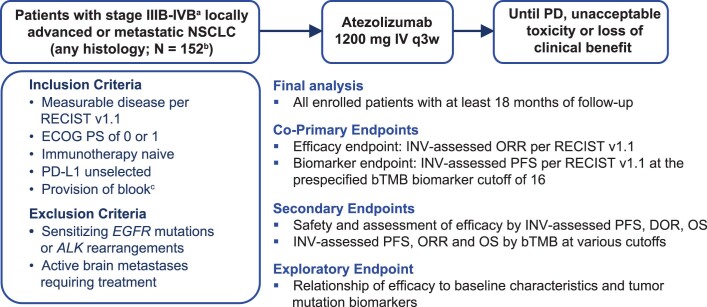
B-F1RST study design. B-F1RST prospectively evaluated atezolizumab monotherapy in the first-line treatment of patients with NSCLC. bTMB, blood-based tumor mutational burden; DOR, duration of response; ECOG PS, Eastern Cooperative Oncology Group performance status; INV, investigator; NSCLC, non-small cell lung cancer; OS, overall survival; PD, progressive disease; PD-L1, programmed death-ligand 1; PFS, progression-free survival; RECIST v1.1, Response Evaluation Criteria in Solid Tumors version 1.1. ^a^ Staging based on International Association for the Study of Lung Cancer Lung Cancer Staging Project 8th Edition of the TNM Classification for Lung Cancer^28^. ^b^ Total enrolled, N = 153; however, 1 patient was never treated and was not included in the intention-to-treat population. ^c^ Tissue biopsy was optional.

**Extended Data Fig. 2:**
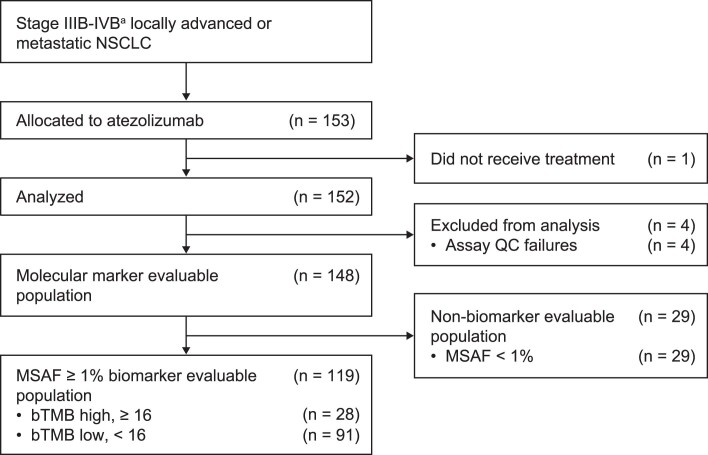
Patient flow. 153 patients were allocated to receive atezolizumab. One patient was not treated and was excluded from the primary analysis. The biomarker-evaluable population excluded patients with MSAF < 1% and assay quality control failures. bTMB, blood-based tumor mutational burden; MSAF, maximum somatic allele frequency; NSCLC, non-small cell lung cancer; QC, quality control. ^a^ Staging based on International Association for the Study of Lung Cancer Lung Cancer Staging Project 8th Edition of the TNM Classification for Lung Cancer^28^.

B-F1RST enrolled patients with immunotherapy-naive stage IIIB–IVB NSCLC, regardless of PD-L1 status, excluding those with *EGFR* mutations or *ALK* alterations (Supplementary Methods). The ITT population excluded one enrolled patient who was not treated (Extended Data Fig. 1). Of the 152 patients in the ITT population, 119 patients (78%) had adequate ctDNA (MSAF ≥ 1%) to generate a valid bTMB result (the biomarker-evaluable population). Samples with MSAF < 1% were not evaluable due to reduced sensitivity of the bTMB assay. The MSAF ≥ 1% population had similar baseline characteristics to those of the ITT population except for having a greater sum of longest diameters (SLD) (Supplementary Table 1 and Extended Data Fig. 2). Of the MSAF ≥ 1% population, 28 patients (24%) had bTMB ≥ 16, and 91 patients (76%) had bTMB < 16. When comparing the bTMB ≥ 16 and bTMB < 16 groups, there were notable differences, with the bTMB ≥ 16 group having more patients younger than 65 years of age (36% versus 27%), higher median SLD of baseline tumor lesions (105 mm versus 66 mm), an absence of never-smokers (0% versus 7%) and more patients with squamous histology (46% versus 24%). Additionally, the bTMB ≥ 16 group had fewer PD-L1-positive patients (32% positive versus 39% negative, 29% missing), and the bTMB < 16 group had more PD-L1-positive patients (40% positive versus 24% negative, 36% missing), suggesting that there is no strong correlation between bTMB and PD-L1 status (χ^2^ test *P* = 0.18), consistent with previous observations in OAK and POPLAR^10^. However, it should be noted that 36% of patients in the ITT population were missing a PD-L1 result.

### Efficacy in the ITT population

**Fig. 1:**
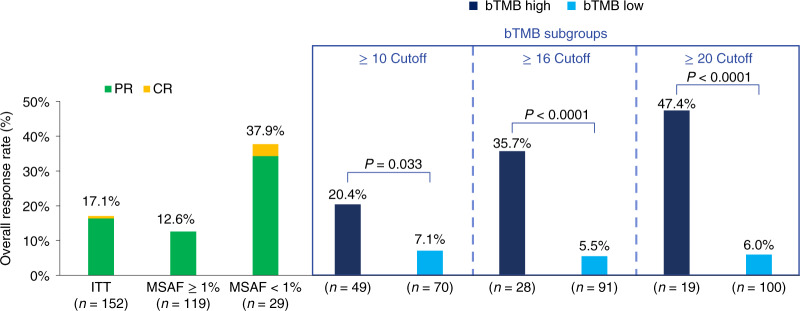
Confirmed responses with atezolizumab. ORRs are shown for bTMB-high (dark blue) and bTMB-low (light blue) subgroups at three different cutoffs, all of which showed significant differences between the subgroups. Data cut: 26 July 2019. Statistical tests (Cochran–Mantel–Haenszel) were unadjusted for multiple comparisons and two-sided at the 0.10 significance level. Rate difference (90% CI): bTMB ≥ 10, 13.3% (2.5, 24.0); bTMB ≥ 16, 30.2% (14.8, 45.6); and bTMB ≥ 20, 41.4% (22.1, 60.6). CR, complete response; PR, partial response.

The primary efficacy endpoint was investigator-assessed overall response rate (ORR) per Response Evaluation Criteria in Solid Tumors version 1.1 (RECIST 1.1). The ORR in the ITT population was 17.1% (95% CI: 11.6, 23.9; Fig. 1). Median duration of response in the ITT population was 16.3 (range, 1.4–23.0) months. Median PFS in the ITT population was 4.1 months (95% CI: 2.8, 4.9), and median OS was 14.8 months (95% CI: 12.7, 21.3).

### Association between efficacy and bTMB

**Fig. 2:**
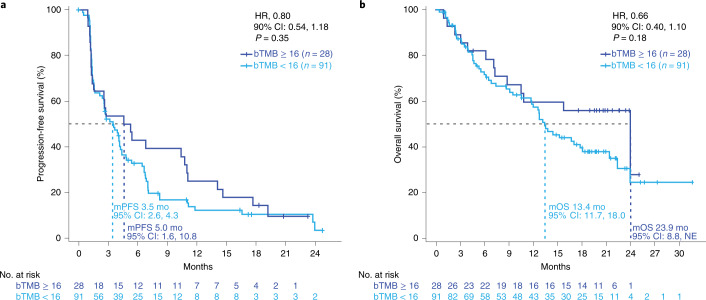
Final analysis outcomes at the bTMB16 cutoff. **a**. Kaplan–Meier survival curves of PFS for bTMB < 16 (light blue) versus bTMB ≥ 16 (dark blue). **b**. Kaplan–Meier survival curves of OS for bTMB < 16 (light blue) versus bTMB ≥ 16 (dark blue). Data cut: 26 July 2019. Statistical tests (log-rank) were unadjusted for multiple comparisons and two-sided at the 0.10 significance level. m, median; mo, months; NE, not estimable.

The primary biomarker endpoint was investigator-assessed PFS per RECIST 1.1 at the pre-specified cutoff of bTMB ≥ 16, which was previously determined in the POPLAR study and validated in the OAK trial^10^. Statistical tests for PFS and OS HRs between groups above and below each bTMB cutoff were two-sided at a 0.10 significance level. The primary biomarker endpoint was not met. At the pre-specified cutoff of bTMB ≥ 16, the median PFS was 5 versus 3.5 months in patients in the bTMB < 16 group (HR = 0.80, 90% CI: 0.54, 1.18, *P* = 0.35; Fig. 2a). The primary biomarker endpoint of PFS at the bTMB ≥ 10 cutoff was not formally tested owing to the study’s hierarchical design. Median OS was 23.9 months in the bTMB ≥ 16 group versus 13.4 months in the bTMB < 16 group (HR = 0.66, 90% CI: 0.40, 1.10, *P* = 0.18; Fig. 2b).

In a secondary biomarker analysis, ORR in the bTMB ≥ 16 versus bTMB < 16 subgroups was 35.7% (95% CI: 19.2, 55.5) versus 5.5% (95% CI: 2.2, 12.2) (*P* < 0.0001). ORR (95% CI) in the bTMB ≥ 10 versus bTMB < 10 groups was 20.4% (10.5, 33.7) versus 7.1% (2.9, 15.3). ORR (95% CI) for bTMB:≥ 20 versus bTMB < 20 was 47.4% (25.2, 69.1) versus 6.0% (2.6, 12.2).

**Fig. 3:**
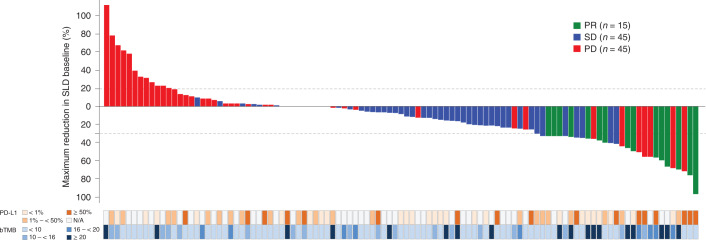
Depth of response according to PD-L1 and bTMB categories. This waterfall plot shows the maximum change in SLD from baseline by response category. PD-L1^a^ and bTMB status by category are displayed below the graph. Patients without a post-baseline SLD assessment were not included. Data cut: 26 July 2019. N/A, not available; PD, progressive disease; PR, partial response; SD, stable disease. ^a^ PD-L1 status was determined by any standard-of-care assay.

The patients with bTMB ≥ 20 and bTMB ≥:16 to bTMB < 20 were predominately clustered in the responder groups (Fig. 3). There was little overlap between the patients with bTMB ≥ 16 and those with PD-L1 tumor proportion score (TPS) ≥ 50%, and bTMB ≥ 16 (yes versus no) was not significantly associated with TPS ( < 1%, ≥ 1% to < 50% and ≥ 50%) among patients shown in Fig. 3 (*n* = 64, *P* = 0.82), with 29% missing PD-L1 results.

**Fig. 4:**
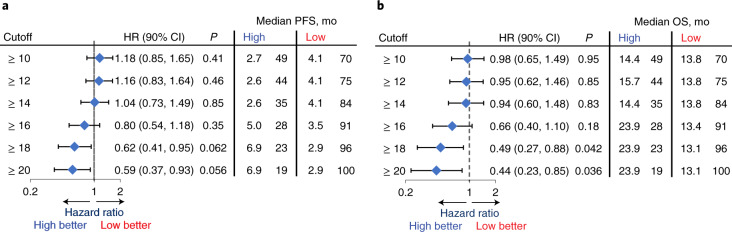
PFS and OS HRs at different bTMB cutoffs. **a**, PFS by bTMB cutoff. **b**, OS by bTMB cutoff. *n* refers to the number of patients in the respective subgroup. The blue diamonds indicate the HR. Statistical tests (log-rank) were unadjusted for multiple comparisons and two-sided at the 0.10 significance level. The error bars show the 90% CI. mo, months.

**Fig. 5:**
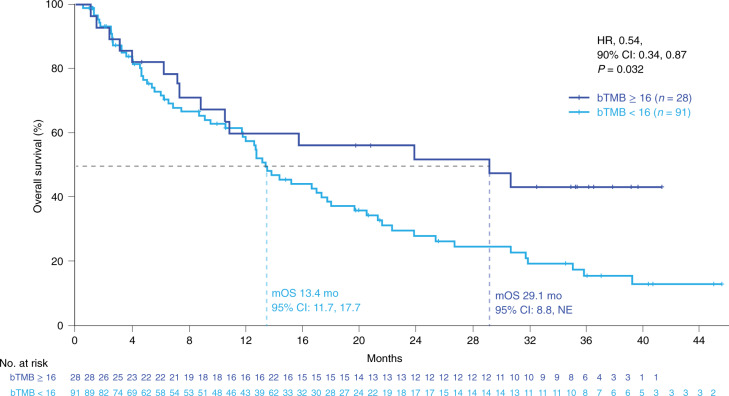
Long-term follow-up OS. Kaplan–Meier plots of OS at the bTMB ≥ 16 cutoff. Statistical tests (log-rank) were unadjusted for multiple comparisons and two-sided at the 0.10 significance level. m, median; mo, months; NE, not estimable.

In the secondary analyses, the HRs for both PFS and OS monotonically improved with increasing bTMB cutoff, with bTMB ≥ 18 and bTMB ≥ 20 crossing the significance boundary of 0.1 for both PFS and OS. (Fig. 4a). At bTMB ≥ 10, the PFS HR was 1.18 (90% CI: 0.85, 1.65, *P* = 0.41), whereas, at bTMB ≥ 18, the PFS HR was 0.62 (90% CI: 0.41, 0.95, *P* = 0.062), and, at bTMB ≥ 20, the PFS HR was 0.59 (90% CI: 0.37, 0.93, *P* = 0.056). Similar trends were noted for OS where, at bTMB ≥ 10, OS HR was 0.98 (90% CI: 0.65, 1.49, *P* = 0.95), whereas, at bTMB ≥ 18, the PFS HR was 0.49 (90% CI: 0.27, 0.88, *P* = 0.042), and, at bTMB ≥ 20, the HR was 0.44 (90% CI: 0.23, 0.85, *P* = 0.036; Fig. 4b). However, the trend toward better outcomes with increasing bTMB cutoff was accompanied by a decrease in the number of patients with higher bTMB. Additionally, with longer follow-up at the 2 December 2020 clinical cutoff date (median duration of follow-up, 36.5 months), OS increased for the bTMB ≥ 16 group (median OS, 29.1 versus 13.4 months for bTMB ≥ 16 versus bTMB < 16; HR = 0.54, 90% CI: 0.34, 0.87, *P* = 0.032; Fig. 5).

### Subgroup analyses by ctDNA fraction

Patients whose ctDNA levels were low (MSAF < 1%) were non-evaluable for bTMB and were defined as the MSAF < 1% population. These patients had a higher response rate than patients with MSAF ≥ 1% (ORR 37.9% versus 12.6%, odds ratio (OR) = 4.2, 95% CI: 1.7, 10.7; Fig. 1). The single complete responder in the ITT population was in the MSAF < 1% subgroup.

We conducted a propensity score analysis using an inverse probability weighting (IPW) model to explore potential differences among groups in baseline characteristics that could account for the higher ORR seen in the MSAF < 1% population^12^. We compared baseline characteristics between the MSAF < 1% and MSAF ≥ 1% subgroups and calculated *P* values. Baseline characteristics with a notable difference between subgroups (defined, in this case, as *P* < 0.15) included median age (65 versus 70 years, *P* = 0.03), current smokers (14% versus 25%, *P* = 0.14), PD-L1-positive status (52% versus 38%, *P* = 0.13), mean number of target lesions (1.8 versus 2.4, *P* = 0.02) and tumor size (median SLD: 42.4 mm versus 70.0 mm, *P* = 0.001) and were included in the IPW model (Supplementary Table 2).

**Extended Data Fig. 3:**
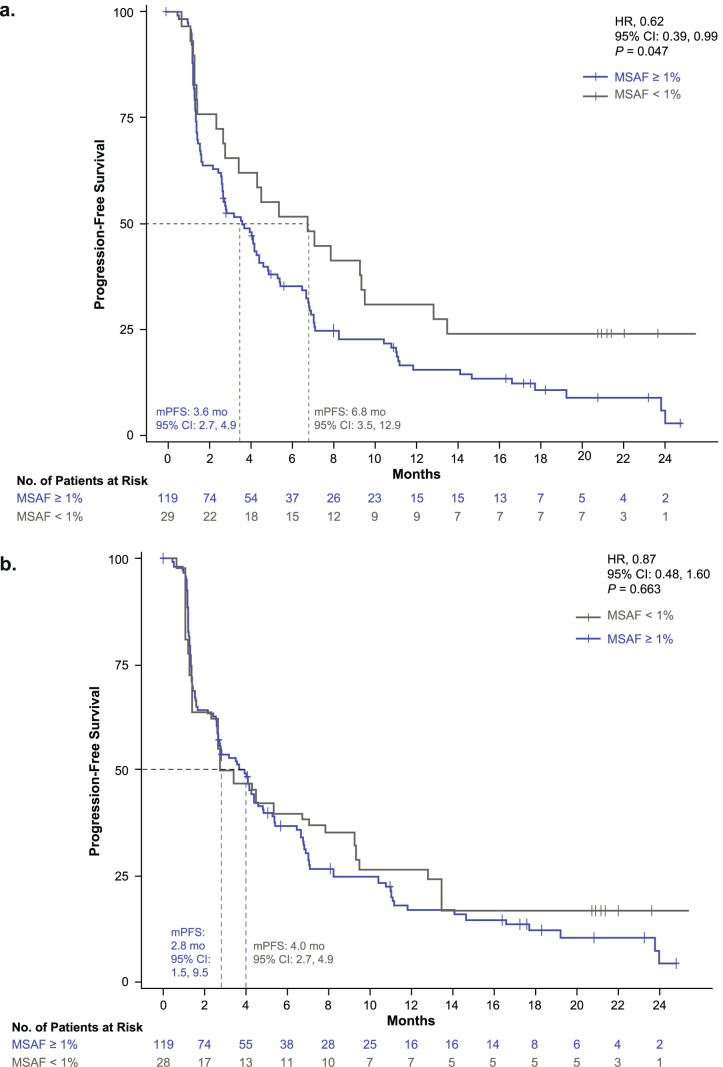
Unadjusted and adjusted MSAF-associated PFS. **a**. Kaplan–Meier plots of unadjusted PFS. **b**. Kaplan–Meier plot of PFS adjusted for baseline imbalances. After the PFS curves were adjusted, the difference between arms was not significant. Statistical tests (log-rank) were unadjusted for multiple comparisons and 2-sided at the 0.05 significance level. HR, hazard ratio; m, median; MSAF, maximum somatic allele frequency; PFS, progression-free survival.

After employing the IPW model, the differences between the baseline characteristics for the adjusted MSAF groups were not significant (Supplementary Table 2). Objective responses were estimated for the adjusted MSAF populations. The confirmed objective responses for the adjusted MSAF < 1% and MSAF ≥ 1% populations were 20.3% and 13.4%, respectively (OR = 1.7, 95% CI: 0.3, 8.7, *P* = 0.56) and were not significantly different. The unadjusted PFS medians for the MSAF < 1% and MSAF ≥ 1% populations were 6.8 months and 3.6 months (HR = 0.62, 95% CI: 0.39, 0.99, *P* = 0.047; Extended Data Fig. 3a). After adjustment, PFS medians were 4.0 months and 2.8 months (HR = 0.87; 95% CI: 0.48, 1.6, *P* = 0.66; Extended Data Fig. 3b).

### Safety data

The safety-evaluable population included 152 patients. The dose intensity (number of doses received divided by the expected number of doses) was 97.7% (minimum to maximum, 60–102), and a median of six (range, 1–39) doses were administered.

**Fig. 6:**
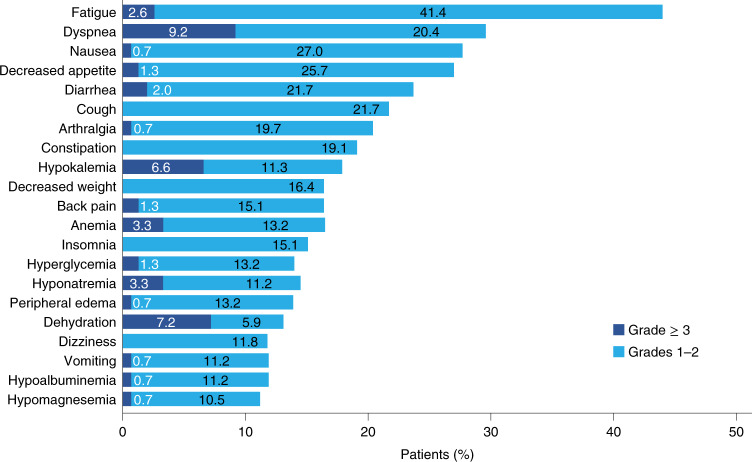
Atezolizumab AEs observed in ≥ 10% of the ITT population. The incidence of AEs by grade ≥ 3 (dark blue) and grade 1–2 (light blue).

Treatment-related adverse events (AEs) occurred in 76% of patients (Supplementary Table 3). AEs leading to treatment discontinuation occurred in 18% of patients. The three most frequent AEs of special interest were skin and subcutaneous events (20%) and aspartate aminotransferase elevation and hypothyroidism (9% each) (Supplementary Table 4). The three most frequent all-grade AEs were fatigue (44%), dyspnea (30%) and nausea (28%) (Fig. 6). One patient died of treatment-related respiratory failure (Supplementary Table 3).

### Genomic analysis of the bTMB-high population

**Extended Data Fig. 4:**
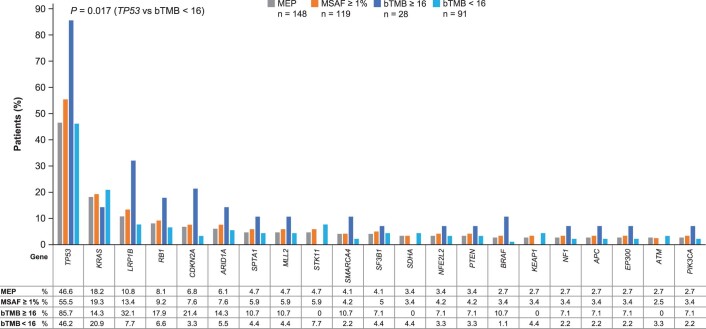
Mutant genes in bTMB subgroups with > 2% frequency. **a**. The prevalence of genes in the MEP, MSAF ≥ 1%, bTMB ≥ 16 and bTMB < 16 populations. The bTMB-high population was enriched in *TP53* mutations compared with the other groups. Differences for all other genes were not significant. bTMB, blood-based tumor mutational burden; MEP, molecularly evaluable population; MSAF, maximum somatic allele frequency. ^a^Does not include *FANCD2*.

An exploratory analysis was conducted on the molecularly evaluable population (MEP), defined as patients who had an evaluable blood sample at baseline (*n* = 148). Patients with any MSAF values were included, as well as those with either driver or insertion and/or deletion mutations (Methods). Genes mutated in ≥ 2% of the B-F1RST MEP, along with prevalence in the MSAF ≥ 1%, bTMB ≥ 16 and bTMB < 16 groups, are shown in Extended Data Fig. 4. Alterations in *TP53*, *LRP1B* and *CDKN2A* appear to be enriched in the bTMB ≥ 16 group versus the bTMB < 16 group, but only *TP5*3 was significantly associated with the bTMB ≥ 16 group (false discovery rate adjusted *P* = 0.017).

## Discussion

The B-F1RST study was designed to evaluate bTMB and its association with clinical outcomes in patients with advanced or metastatic NSCLC treated with first-line atezolizumab. At the pre-specified bTMB cutoff of ≥ 16, PFS was not statistically different between the high versus low groups, although a numerical improvement in PFS was observed. The lack of statistical significance may be attributed to the smaller biomarker-evaluable population of 119 relative to the originally planned 150 patients, resulting in a reduction in statistical power. With this population size, a PFS HR of 0.55 between high versus low groups would have been needed to detect a statistically significant difference (Methods). Although *P* values for the other analyses are descriptive, secondary analyses showed that ORR was significantly better in the bTMB ≥ 16 group versus the bTMB < 16 group. This increased benefit of atezolizumab monotherapy was evident despite the bTMB ≥ 16 group having more indicators of poorer prognosis than the bTMB < 16 group, including a higher baseline SLD and more patients with squamous histology^13–15^. Secondary and exploratory analyses showed OS improvement between high and low groups at the bTMB ≥ 20 cutoff, even though OS was not statistically better at the bTMB ≥ 16 cutoff. However, a final exploratory analysis after 36.5 months of follow-up showed a longer OS in the bTMB ≥ 16 versus bTMB < 16 group (median OS, 29.1 versus 13.4 months, HR = 0.54, 90% CI: 0.34, 0.87, *P* = 0.032). Atezolizumab monotherapy was well tolerated, and no new safety signals were observed.

The B-F1RST bTMB results are consistent with data from other first-line studies of checkpoint inhibitors in NSCLC that have evaluated TMB. In CheckMate026, patients with PD-L1 > 5% of TCs and high tTMB who were treated with nivolumab had longer PFS and higher ORR than patients treated with chemotherapy, but the difference in OS was not significant^2^. In CheckMate227, low-dose ipilimumab plus nivolumab significantly improved median PFS versus chemotherapy in patients with tTMB ≥ 10 mut/Mb but not with tTMB < 10 mut/Mb. The OS benefit was similar regardless of tTMB status, PD-L1 status or any combination of the two^16^. In MYSTIC, the OS benefit for durvalumab plus tremelimumab versus chemotherapy was greater in patients with bTMB ≥ 16 mut/Mb than those with bTMB < 16 mut/Mb^17^. Finally, in IMpower110, bTMB ≥ 16 was associated with a longer PFS benefit than bTMB < 16 in patients treated with atezolizumab versus chemotherapy^1^. It should be noted that IMpower110 enrolled only PD-L1-positive patients; therefore, the bTMB ≥ 16 patients were selected for two biomarkers. The results from B-F1RST, on the other hand, suggest that bTMB may be associated with atezolizumab benefit in a PD-L1-unselected, bTMB-high population. However, as B-F1RST did not require collection of tumor tissue, the independent roles of bTMB versus PD-L1 in the first-line setting cannot be definitively addressed.

bTMB has been prospectively evaluated in the phase 3 randomized Cohort C of the BFAST trial as a predictive biomarker in the first-line treatment of NSCLC with atezolizumab versus platinum-based chemotherapy. Although Cohort C did not meet its primary endpoint, there was a trend toward a PFS benefit in patients with bTMB ≥ 16 who received atezolizumab^18^.

Considering the totality of the recent data in the field, tTMB and bTMB are relatively weak predictive biomarkers for first-line treatment of NSCLC in chemotherapy-free combination immunotherapy or monotherapy settings but may still be of value in patients whose tumors do not express high levels of PD-L1 or in the case of patients who have high bTMB and inadequate tissue for PD-L1 testing^19^. Moreover, TMB has been shown to have predictive value in later lines of therapy and in other indications, which is supported by recent approval of pembrolizumab in the TMB-high metastatic pan-tumor setting^20^. However, the strength of TMB as a predictive biomarker has been variable.

Part of the reason for the variability in TMB results may be that tTMB and bTMB are surrogate biomarkers in that they measure a phenotype that is indirectly related to neoantigen load, which itself is distal to the direct action of anti-PD-L1/PD-1 therapies. Moreover, tTMB and bTMB do not provide information on the antigenicity of the relevant neoantigens involved in tumor/immune response, nor do they provide information on the capacity of tumors to present antigens, both of which are likely to be relevant to the overall response to anti-PD-L1/PD-1 therapy. As such, the biological difference between a surrogate such as TMB and the actual biological variable (for example, neoantigen quality or quantity) is likely to vary between tumors.

Furthermore, developments in assay technology and a deeper understanding of TMB and its biological consequences may be needed to select the population that will benefit most from checkpoint inhibitors. Insertion/deletion mutations may produce more antigenic neoantigens than single-nucleotide variants^21^, and the fact that insertion/deletion mutations were not included in the bTMB assay used in this study could have influenced the findings. In addition, biomarkers that may inform new bTMB algorithms are being investigated^22^. Variables such as clonality of neoantigens, MHC-1 genotype^23^, human leukocyte antigen loss of heterozygosity, T cell receptor repertoire, other genomic alterations that might affect immune response and other immune considerations, including T cell levels^24^, have shown promise for informing new bTMB algorithms in the future. Another factor that may need to be re-evaluated is the cutoff, and using a higher threshold than bTMB ≥ 16 might have shown improved PFS and OS, as the high versus low subgroups at bTMB ≥ 18 and bTMB ≥ 20 cutoffs derived significant benefit on both of these outcomes, albeit in smaller patient populations. The ORR for the high versus low subgroups at bTMB ≥ 20 was also improved compared to the ORR for the subgroups at the bTMB ≥ 16 cutoff. Finally, bTMB might also be used to predict the benefit of checkpoint inhibitor therapy in combination with other biomarkers, including PD-L1. Although the dataset for PD-L1 status in B-F1RST is incomplete (36% of patients had unknown PD-L1 status), the higher baseline prevalence of PD-L1-negative patients in the bTMB ≥ 16 group, together with the higher prevalence of PD-L1-positive patients in the bTMB < 16 group, support the concept of PD-L1 expression and bTMB as independent predictive biomarkers with a limited overlap between high-TMB and high-PD-L1 patient populations^10,25^. Therefore, PD-L1-positive patients who benefit from atezolizumab may be a distinct group from bTMB-high patients, who also may benefit. However, further study is needed to determine how these two biomarkers might be used in combination to predict patient outcomes.

Patients with low ctDNA levels (MSAF < 1%) also had significantly longer PFS with atezolizumab monotherapy than patients with MSAF ≥ 1% (Extended Data Fig. 3), consistent with previous reports^1,26,27^. Indeed, low MSAF is associated with favorable prognostic factors, such as lower age, current non-smoking status, PD-L1-positive status, fewer lesions or lower overall tumor burden (Supplementary Table 2). Notably, when these prognostic factors were accounted for, our IPW model showed that low MSAF as a marker of benefit was not independent from these other baseline factors.

Major limitations of this study include its single-arm design, small number of patients and lack of PD-L1 data. The genomic results have the additional limitations in that the next-generation sequencing pipeline used was exploratory and had limited depth of sequencing for mutation calls, as the assay was designed to measure bTMB.

In conclusion, the greater ORR, the trend toward increasing OS and PFS benefit that we observed at higher bTMB cutoffs in patients with NSCLC treated with first-line atezolizumab monotherapy, and the longer OS at longer-term follow-up in patients with bTMB ≥ 16 together suggest that bTMB may be a predictive biomarker for atezolizumab benefit with additional development. Further exploration of the biologic mechanisms of TMB as it relates to checkpoint inhibitor therapy, along with refinement of the bTMB assay and additional clinical validation, will be necessary before such selection parameters can be employed in the clinical setting.

## Methods

### Study design

B-F1RST is registered with ClinicalTrials.gov as NCT02848651. The study protocol is available as a supplementary file. A total of 153 patients with stage IIIB–IVB locally advanced or metastatic NSCLC were enrolled from 20 regional and community practice sites in the United States. The study design and key inclusion criteria are shown in Extended Data Fig. 1. Patients were treated with atezolizumab 1,200 mg intravenously every 3 weeks until disease progression, unacceptable AEs or loss of clinical benefit. Co-primary endpoints were investigator-assessed ORR (RECIST 1.1) and investigator-assessed PFS. A pre-specified bTMB cutoff of 16 was used to evaluate efficacy, which was equivalent to ≈14.5 mut/Mb (16 mut/1.1 Mb). Kaplan–Meier curves and a log-rank test were used to evaluate the differences in PFS between bTMB-high and bTMB-low groups. Tests between bTMB-high and bTMB-low subgroups at each cutoff were two-sided at a 0.10 significance level. To balance speed of enrollment while minimizing type I error, this significance level for co-primary biomarker endpoints has been used in phase 2 studies primarily as proof of concept for phase 3 confirmatory studies. Secondary endpoints included duration of response, PFS and OS. Exploratory endpoints explored the relationship among efficacy, baseline characteristics and biomarkers. Secondary and exploratory biomarker analyses were not adjusted for multiple comparisons. Therefore, *P* values presented were for descriptive purposes only. Safety was assessed by rates of AEs and changes in laboratory test results. Based on a population of 150 patients, the study was designed to have 80% power to detect statistical difference in PFS if the PFS HR was 0.6 between bTMB-high and bTMB-low subgroups, based on a two-sided significance level of 0.1. A post hoc analysis of the power based on the 119 biomarker-evaluable patients who were actually enrolled showed that the study had an 80% power to detect a statistical difference at the 0.1 level if the PFS HR was 0.55 between bTMB-high and bTMB-low subgroups. B-F1RST was approved by the relevant institutional review board (IRB) and ethics committee for each participating center, including Advarra and WCG IRB (formerly WIRB-Copernicus Group, Inc.), and was performed in full accordance with the guidelines for Good Clinical Practice and the Declaration of Helsinki, and all patients gave written informed consent.

### Propensity score model

Baseline factors were compared between the MSAF < 1% and MSAF ≥ 1% subgroups. Factors with notable difference between the groups (*P* < 0.15) were included in a model to adjust for baseline imbalances using the IPW method. Factors were included in a propensity score model to estimate probability of being in either the MSAF < 1% or MSAF ≥ 1% group. The IPW used probabilities from the propensity model to estimate efficacy by adjusting for imbalances in baseline factors. It was used because prognostic and predicted factors could not be directly adjusted due to the small sample sizes of the MSAF < 1% (*n* = 29) and MSAF ≥ 1% (*n* = 119) groups. The model was used to estimate ORR and PFS, adjusted for baseline imbalances.

### Determination of bTMB status and contributing genetic alterations

The methodology for bTMB determination from DNA extraction through computational pipeline was a proprietary assay developed by Foundation Medicine^10^. In brief, cfDNA was extracted, and 20–100 ng was used for library construction. A set of specific, designed, fragment-level indexed adaptors was ligated randomly onto both ends of each input duplex of cfDNA fragment, and select genes were pulled down using hybrid capture technology targeting 394 genes or 1.1 Mb of coding region of the human genome. Samples were sequenced on Illumina HiSeq 4000 to at least 800× median exonic coverage.

#### Analysis methods

The bTMB score was determined by identifying all base substitutions present at an allele frequency of ≥ 0.5% across the coding region of 394 genes (≈1.1 Mb) and filtering out germline events by comparing against the dbSNP and ExAC databases in samples where the MSAF was ≥ 1%. Rare germline events were filtered using the somatic-germline-zygosity algorithm^10^, and further filtering removed known driver alterations to minimize the bias associated with the genes used for capture. The estimation of the tumor fraction by the MSAF was defined according to the highest allele fraction for confirmed somatic base substitutions of less than 20%, regardless of their driver status. The bTMB assay required a minimum MSAF of ≥ 1%.

#### Exploratory biomarker analysis

An exploratory computational pipeline was developed to call alterations using the bTMB sequencing data. Non-synonymous single-nucleotide variants and insertions and/or deletions were analyzed. Copy number variants were not determined. Single-nucleotide variants were validated to 0.5% allele frequency. Insertions and/or deletions were not validated in this platform but are validated at 1% on FoundationOneCDx (Foundation Medicine), which uses the same bait set as the bTMB assay.

Somatic-germline-zygosity filtering was not applied to MEP, so germline alterations and clonal hematopoiesis of indeterminate potential mutations may be present, and driver mutations were not removed. Quality control of genes with prevalence of ≥ 2% was manually performed. Prevalence of genes in the B-F1RST MEP was compared to a Foundation Medicine database to identify artifacts. Normalization for gene size was not performed. Predicted known and likely alterations are shown in Extended Data Fig. 4.

#### Statistical methods

SAS (version 9.4) software was used for all analyses except for the exploratory biomarker analysis, which used R (version 3.5.3).

### Analysis population

Efficacy and safety analyses included all patients who received at least one dose of study drug. Biomarker analyses included all patients who received at least one dose of study drug and had a baseline biomarker assessment.

### Primary efficacy endpoint and analysis

The primary efficacy endpoint of this study was confirmed investigator-assessed ORR, defined as the proportion of patients whose confirmed best overall response was either a partial response or a complete response per RECIST 1.1. An estimate of the ORR for all patients who received study drug and the exact 95% CI were calculated by using the Blaker method.

### Primary biomarker endpoint and analysis

Kaplan–Meier curves and a log-rank test were used to evaluate the differences in investigator-assessed PFS between bTMB-high (bTMB ≥ 16) and bTMB-low (bTMB < 16) groups. Tests were two-sided at a significance level of 0.10 for this phase 2 study.

### Secondary efficacy and biomarker endpoints and analysis

Secondary efficacy endpoints included OS, investigator-assessed duration of response and PFS per RECIST 1.1. Secondary biomarker cutoff points included baseline bTMB at various cutoff points (10–20 by intervals of 2) besides 16.

Kaplan–Meier curves and log-rank tests were used to evaluate the differences in PFS and OS between bTMB-high and bTMB-low groups at various cutoff points. Descriptive statistics for PFS and OS curves, including median PFS time and 6-, 9- and 12-month PFS probabilities, were estimated for various cutoff points as well. The Brookmeyer–Crowley methodology was used to construct the 95% CI for the medians for PFS and OS. The Greenwood formula was used to construct the 95% CI for the landmark PFS. Association between confirmed investigator-assessed ORR and baseline bTMB were evaluated at various biomarker cutoff points using the Cochran–Mantel–Haenszel test.

### Exploratory biomarker analyses

Associations between gene mutation and bTMB-high and bTMB-low status at the bTMB cutoff of 16 were also explored. *P* values were adjusted using the false discovery rate adjustment method to address multiple gene testing.

### Reporting Summary

Further information on research design is available in the Nature Research Reporting Summary linked to this article.

## Online content

Any methods, additional references, Nature Research reporting summaries, source data, extended data, supplementary information, acknowledgements, peer review information; details of author contributions and competing interests; and statements of data and code availability are available at 10.1038/s41591-022-01754-x.

## Supplementary information

Supplementary Information Supplementary Tables 1–4.

Reporting Summary.

Supplementary Data The final version of the B-F1RST trial protocol.

## Data Availability

To minimize the risk of patient re-identification, data will only be shared upon reasonable request. For eligible studies, qualified researchers may request access to individual patient-level clinical data through a data request platform. At the time of writing, this request platform is Vivli (https://vivli.org/ourmember/roche/). Datasets can be requested 18 months after a clinical study report has been completed and, as appropriate, once the regulatory review of the indication or drug has completed. As this has since passed for this trial, access to patient-level data from this trial can now be requested and will be assessed by an independent review panel, which decides whether the data will be provided. Once approved, the data are available for up to 24 months. For up-to-date details on Roche’s Global Policy on the Sharing of Clinical Information and how to request access to related clinical study documents, see https://go.roche.com/data_sharing. Anonymized records for individual patients across more than one data source external to Roche can not, and should not, be linked owing to a potential increase in risk of patient re-identification. Figures with associated raw data include the main text, Figs. 1–6 and Extended Data Figs. 3 and 4. The dbSNP (https://www.ncbi.nlm.nih.gov/snp/) and ExAC (https://gnomad.broadinstitute.org/) databases were used in this research.
