# Supplementary material for: Blood-based tumor mutational burden as a biomarker for atezolizumab in non-small cell lung cancer: the phase 2 B-F1RST trial
Source: Nat Med. 2022 Apr 14;28(5):939–45. doi: 10.1038/s41591-022-01754-x (PMC9117143; doi:10.1038/s41591-022-01754-x)
Supplement: Supplementary file 1 — Supplementary Tables 1–4. [file 41591_2022_1754_MOESM1_ESM.pdf]

nature portfolio

https://doi.org/10.1038/s41591-022-01754-x

Supplementary information

Blood-based tumor mutational burden as a biomarker for atezolizumab in non-small cell lung cancer: the phase 2 B-F1RST trial

In the format provided by the authors and unedited

Online supplement

Supplementary Tables

Table S1. Patient demographics and characteristics

Table S2. Unadjusted and adjusted baseline characteristics between MSAF<1% and MSAF≥1% groups

Table S3. Safety summary

Table S4. AEs of special interest

Confidential. DO NOT DISTRIBUTE.

Table S1. Patient demographics and characteristics

Characteristic	ITT (N = 152)	MSAF ≥ 1% (n = 119)	MSAF ≥ 1%	
			bTMB < 16 (n = 91)	bTMB ≥ 16 (n = 28)
Age, median (range), y	69 (39-90)	70 (47-90)	70 (47-90)	70 (52-89)
Age group, n (%)				
< 65 y	51 (34)	35 (29)	25 (27)	10 (36)
≥ 65 y	101 (66)	84 (71)	66 (73)	18 (64)
Sex, n (%)				
Male	83 (55)	66 (55)	47 (52)	19 (68)
Female	69 (45)	53 (45)	44 (48)	9 (32)
Race, n (%)				
American Indian/Alaska Native	1 (0.7)	1 (0.8)	1 (1)	0
Asian	1 (0.7)	0	0	0
Black	13 (9)	9 (8)	8 (9)	1 (4)
White	135 (89)	107 (90)	80 (88)	27 (96)
Unknown	2 (1)	2 (2)	2 (2)	0
Smoking status, n (%)				
Never	11 (7)	6 (5)	6 (7)	0
Current/previous	141 (93)	113 (95)	85 (93)	28 (100)
ECOG PS, n (%)				
0	40 (26)	29 (24)	21 (23)	8 (29)
1	112 (74)	90 (76)	70 (77)	20 (71)
Study entry staging, n (%)				
IIIB	11 (7)	9 (8)	9 (10)	0
IVA	68 (45)	52 (44)	40 (44)	12 (43)
IVB	73 (48)	58 (49)	42 (46)	16 (57)
Histology, n (%)				
Non-squamous	110 (72)	84 (71)	69 (76)	15 (54)
Squamous	42 (28)	35 (29)	22 (24)	13 (46)
PD-L1 IHC, n (%)[a]				
Negative	37 (24)	33 (28)	22 (24)	11 (39)
Positive	61 (40)	45 (38)	36 (40)	9 (32)
Missing	54 (36)	41 (34)	33 (36)	8 (29)
MSAF, median (range), %	N/A	4.0 (1.0, 19.4)	3.3 (1.0, 19.4)	10.7 (1.9, 19.4)[a]
SLD, median (range), mm	63 (13-257)	70 (13-257)	66 (13-212)	105 (13-257)
Prior radiation therapy, n (%)	60 (39)	43 (36)	34 (37)	9 (32)
Prior neoadjuvant/adjuvant therapy, n (%)	8 (5)	6 (5)	5 (5)	1 (4)

Confidential. DO NOT DISTRIBUTE.

Data cut: July 26, 2019. bTMB, blood-based tumor mutational burden; ECOG PS, Eastern Cooperative Oncology Group performance status; IHC, immunohistochemistry; ITT, intention to treat; MSAF, maximum somatic allele frequency; N/A, not available; PD-L1, programmed death-ligand 1; SLD, sum of the longest diameters. [a] $P < 0.001$ for bTMB≥16 vs <16.

Confidential. DO NOT DISTRIBUTE.

Table S2 Unadjusted and adjusted baseline characteristics between MSAF<1% and MSAF≥1% groups

Characteristic	Unadjusted			Adjusted		
	**MSAF < 1%**	**MSAF ≥ 1%**	***P* value**	**MSAF < 1%**	**MSAF ≥ 1%**	***P* value**
No. of patients, n	29	119		28	119	
Age, median (range), years	65 (39-84)	70 (47-90)	0.03	66 (39-84)	69 (47-90)	0.53
Age group, n (%)						
< 65 years	14 (48)	35 (29)	0.086	8 (30)	39 (33)	0.80
≥ 65 years	15 (52)	84 (71)		19 (70)	80 (67)	
Sex, n (%)						
Female	14 (48)	53 (45)	0.88	12 (44)	53 (45)	0.96
Male	15 (52)	66 (55)		15 (56)	66 (55)	
Tobacco history, n (%)						
Never	4 (14)	6 (5)	0.14	2 (7)	8 (7)	0.52
Current	4 (14)	30 (25)		4 (13)	27 (23)	
Previous	21 (72)	83 (70)		22 (80)	84 (71)	
Pathology/histology per eCRF, n (%)						
Non-squamous	23 (79)	84 (71)	0.48	19 (68)	86 (72)	0.75
Squamous	6 (21)	35 (29)		9 (32)	33 (28)	
Study entry staging, n (%)						
IIIB	1 (3)	9 (8)	0.71	1 (2)	9 (7)	0.45
IVA	14 (48)	52 (44)		11 (39)	53 (44)	
IVB	14 (48)	58 (49)		16 (59)	58 (48)	
ECOG PS score, n (%)						
0	9 (31)	29 (24)	0.62	8 (28)	30 (25)	0.76
1	20 (69)	90 (76)		20 (72)	89 (75)	
PD-L1 IHC result, n (%)						
Negative	3 (10)	33 (28)	0.13	4 (15)	29 (24)	0.65
Positive	15 (52)	45 (38)		14 (49)	49 (41)	
Missing	11 (38)	41 (34)		10 (36)	42 (35)	
No. of target lesions, mean (SD)	1.8 (0.9)	2.4 (1.2)	0.02	2.3 (1.1)	2.3 (1.2)	1.00
Sum of longest diameters, median (range), mm	42.4 (13.0-200.0)	70.0 (12.7-257.0)	0.001	48.2 (13.0-200.0)	64.8 (12.7-257.0)	0.77

Data cut: July 26, 2019. T-tests (for continuous variables) and chi-square tests (for categorical variables) were unadjusted for multiple comparisons and at the 0.15 two-sided significance level. ECOG, Eastern Cooperative Oncology Group performance status; eCRF, electronic case report form; IHC, immunohistochemistry; ITT, intention to treat; MSAF, maximum somatic allele frequency; PD-L1, programmed death-ligand 1.

Confidential. DO NOT DISTRIBUTE.

Table S3. Safety summary (N = 152)

Patients in ITT	n (%)
All-Grade AEs, any cause<br>Treatment-related AEs	152 (100)<br>116 (76)
All-Grade SAEs, any cause<br>Treatment-related SAEs	81 (53)<br>22 (14)
Grade 3-4 AEs, any cause<br>Treatment-related Grade 3-4 AEs	86 (57)<br>30 (20)
Grade 5 AEs, any cause<br>Treatment-related Grade 5 AEs	10 (7)[a]<br>1(1)
AEs leading to treatment discontinuation	27 (18)
AEs of special interest (immune related)	66 (43)

Data cut: July 26, 2019. AE, adverse event; ITT, intention to treat, SAE, serious adverse event. [a] Grade 5 events reported as respiratory failure (n = 2, 1 treatment related) and death, cardiac arrest, pulmonary embolism, acute respiratory failure, dyspnea, pneumonia, sepsis, malignant pleural effusion (n = 1 each).

Confidential. DO NOT DISTRIBUTE.

Table S4. AEs of special interest (N = 152)

AEs of special interest per MedDRA PT, n (%)	Overall	Grade 3-4[a]
Skin and subcutaneous[b]	30 (20)	1 (0.7)
AST elevation	13 (9)	1 (0.7)
Hypothyroidism[c]	13 (9)	0
Pneumonitis	12 (8)	6 (4)
ALT elevation	11 (7)	1 (0.7)
Colitis	5 (3)	2 (1)
Hyperthyroidism[c]	2 (1)	0
Adrenal insufficiency	1 (0.7)	1 (0.7)
Keratitis	1 (0.7)	0
Myocarditis	1 (0.7)	0

Data cut: July 26, 2019. AE, adverse event; ALT, alanine aminotransferase; MedDRA, Medical Dictionary for Regulatory Activities; PT, preferred term; TSH, thyroid-stimulating hormone. [a] All Grade 3 events except for colitis (Grade 4). [b] Includes PTs of maculopapular rash, erythema, dermatitis allergic, rash, rash pruritic, skin ulcer, dermatitis, dermatitis acneiform. [c] Does not include PTs such as thyroxine free decreased, tri-iodothyronine free decreased, blood TSH decreased.

Confidential. DO NOT DISTRIBUTE.
