# Supplementary material for: Blood-based tumor mutational burden as a biomarker for atezolizumab in non-small cell lung cancer: the phase 2 B-F1RST trial
Source: Nat Med. 2022 Apr 14;28(5):939–45. doi: 10.1038/s41591-022-01754-x (PMC9117143; doi:10.1038/s41591-022-01754-x)
Supplement: Supplementary file 2 — Reporting Summary. [file 41591_2022_1754_MOESM2_ESM.pdf]

nature portfolio

Corresponding author(s): Vamsidhar Velcheti

Last updated by author(s): Feburary 10, 2022

Reporting Summary

Nature Portfolio wishes to improve the reproducibility of the work that we publish. This form provides structure for consistency and transparency in reporting. For further information on Nature Portfolio policies, see our Editorial Policies and the Editorial Policy Checklist.

Statistics

For all statistical analyses, confirm that the following items are present in the figure legend, table legend, main text, or Methods section.

n/a	Confirmed	
☐	☒	The exact sample size (*n*) for each experimental group/condition, given as a discrete number and unit of measurement
☐	☒	A statement on whether measurements were taken from distinct samples or whether the same sample was measured repeatedly
☐	☒	The statistical test(s) used AND whether they are one- or two-sided<br>*Only common tests should be described solely by name; describe more complex techniques in the Methods section.*
☐	☒	A description of all covariates tested
☐	☒	A description of any assumptions or corrections, such as tests of normality and adjustment for multiple comparisons
☐	☒	A full description of the statistical parameters including central tendency (e.g. means) or other basic estimates (e.g. regression coefficient) AND variation (e.g. standard deviation) or associated estimates of uncertainty (e.g. confidence intervals)
☐	☒	For null hypothesis testing, the test statistic (e.g. F, t, r) with confidence intervals, effect sizes, degrees of freedom and P value noted<br>*Give P values as exact values whenever suitable.*
☒	☐	For Bayesian analysis, information on the choice of priors and Markov chain Monte Carlo settings
☒	☐	For hierarchical and complex designs, identification of the appropriate level for tests and full reporting of outcomes
☐	☒	Estimates of effect sizes (e.g. Cohen's *d*, Pearson's *r*), indicating how they were calculated

Our web collection on statistics for biologists contains articles on many of the points above.

Software and code

Policy information about availability of computer code

Data collection	No software was used for data collection.
Data analysis	We used R (v3.5.3) for exploratory biomarker analysis and SAS (v9.4) for all of the remaining analyses.

For manuscripts utilizing custom algorithms or software that are central to the research but not yet described in published literature, software must be made available to editors and reviewers. We strongly encourage code deposition in a community repository (e.g. GitHub). See the Nature Portfolio guidelines for submitting code & software for further information.

Data

Policy information about availability of data

All manuscripts must include a data availability statement. This statement should provide the following information, where applicable:

- Accession codes, unique identifiers, or web links for publicly available datasets
- A description of any restrictions on data availability
- For clinical datasets or third party data, please ensure that the statement adheres to our policy

In order to minimize the risk of patient re-identification, data will only be shared upon request. For eligible studies qualified researchers may request access to individual patient level clinical data through a data request platform. At the time of writing this request platform is Vivli. https://vivli.org/ourmember/roche/. Datasets can be requested 18 months after a clinical study report has been completed and, as appropriate, once the regulatory review of the indication or drug has completed. As this has since passed for this trial, access to patient level data from this trial can now be requested and will be assessed by an Independent Review Panel, which decides whether or not the data will be provided. Once approved the data are available for up to 24 months. For up to date details on Roche's Global Policy on the Sharing of Clinical Information and how to request access to related clinical study documents, see here: https://go.roche.com/data_sharing. Anonymized records for individual patients across more than one data source external to Roche can not, and should not, be linked due to a potential increase in risk

of patient re-identification. Figures with associated raw data include main text figures 1-6, and extended data figures 3 and 4. The dbSNP (https://www.ncbi.nlm.nih.gov/snp/) and ExAC (https://gnomad.broadinstitute.org/) databases were used in this research.

Field-specific reporting

Please select the one below that is the best fit for your research. If you are not sure, read the appropriate sections before making your selection.

☒ Life sciences ☐ Behavioural & social sciences ☐ Ecological, evolutionary & environmental sciences

For a reference copy of the document with all sections, see nature.com/documents/nr-reporting-summary-flat.pdf

Life sciences study design

All studies must disclose on these points even when the disclosure is negative.

Sample size	The study was designed to have 80% power to detect statistical difference in PFS between bTMB high and low subgroups, based on a 2-sided significance level of 0.1.
Data exclusions	Data were excluded from the gene analyses (molecular evaluable population) if the mutation prevalence was <2% (not pre-established) due to the low numbers of patients. Data were excluded from the biomarker evaluable population if the maximum somatic allele frequency was <1% (pre-established).
Replication	Biomarkers measured in patient plasma samples were not replicated due to limited availability. The bTMB assay has been analyltically validated previously. Other experiments were clinical in nature and were not replicated.
Randomization	This study was a non-randomized single arm study. Therefore, the control of covariates was not applicable.
Blinding	Because this is a single-arm study, subject were not blinded.

Reporting for specific materials, systems and methods

We require information from authors about some types of materials, experimental systems and methods used in many studies. Here, indicate whether each material, system or method listed is relevant to your study. If you are not sure if a list item applies to your research, read the appropriate section before selecting a response.

Materials & experimental systems

n/a	Involved in the study	
☐	☒	Antibodies
☒	☐	Eukaryotic cell lines
☒	☐	Palaeontology and archaeology
☒	☐	Animals and other organisms
☐	☒	Human research participants
☐	☒	Clinical data
☒	☐	Dual use research of concern

Methods

n/a	Involved in the study	
☒	☐	ChIP-seq
☒	☐	Flow cytometry
☒	☐	MRI-based neuroimaging

Antibodies

Antibodies used	The VENTANA PD-L1 (SP142) rabbit monoclonal primary antibody (RPA; Ventana Medical Systems Inc.) was optimized for use as a fully automated IHC assay on the BenchMark ULTRA (Ventana Medical Systems Inc., Tucson, AZ) staining platform using the OptiView DAB IHC Detection Kit and OptiView Amplification Kit (Ventana Medical Systems Inc., Tucson, AZ). The antibody supplier was Spring Biosciences (catalog number, NA); the clone used was SP142 (19H3L2).
Validation	VENTANA PD-L1 (SP142) assay validation can be found at the following link: https://www.accessdata.fda.gov/cdrh_docs/pdf16/p160002c.pdf

Human research participants

Policy information about studies involving human research participants

Population characteristics	Patients were enrolled with immunotherapy-naïve stage IIIB to IVB NSCLC regardless of PD-L1 status, excluding those with EGFR mutations or ALK alterations. Patients had a median age of 69 years, were 55% male, 89% white, 13% black and 1% each asian and American Indian/Alaska native. 12% had stage IIIB disease, 42% had stage IVA, and 47% had stage IVB. 93% were current or previous smokers. 72% had non-squamous histology and 28% had squamous. 24% were PD-L1 negative, 40% were PD-L1 positive and 36% had a missing result. 39% had prior radiation therapy and 5% had prior neoadjuvant or adjuvant therapy. The median sum of largest diameters was 60 mm.

March 2021

Recruitment	Patients were recruited from 20 sites in the US regional and community practice sites. These sites were selected based on factors such as patient population availability and site staff experience in conducting clinical trials. Regarding patient enrollment, potential patients were approached by the sites and enrolled in the study upon confirmation of eligibility. Because the trial did not require a baseline tissue biopsy, patients enrolled may have been those that did not have sufficient tissue or did not want to undergo a baseline biopsy. These factors, however, would have minimal impact on the interpretation of results as the objectives for the study were to evaluate a blood-based marker.
Ethics oversight	The trial was conducted according to Good Clinical Practice and the Declaration of Helsinki. All patients provided written informed consent. Protocol approval was obtained from independent review boards or ethics committees at each site.

Note that full information on the approval of the study protocol must also be provided in the manuscript.

Clinical data

Policy information about clinical studies

All manuscripts should comply with the ICMJE guidelines for publication of clinical research and a completed CONSORT checklist must be included with all submissions.

Clinical trial registration	NCT02848651
Study protocol	The trial protocol will be provided as a supplementary file. We have added this fact in the methods.
Data collection	Data was collected between September 21, 2016 to May 14, 2019 from 153 patients with stage IIIB-IVB locally advanced or metastatic NSCLC who were enrolled from 20 US regional and community practice sites. Patients were recruited from September 21, 2016 to November 13, 2017.
Outcomes	The primary efficacy endpoint was investigator-assessed objective response rate (ORR) by RECIST v1.1, used to evaluate the clinical efficacy of atezolizumab. Investigator-assessed ORR was defined as the proportion of patients who had a confirmed best overall response of either PR or CR per RECIST v1.1. The secondary efficacy endpoint investigator-assessed DOR by RECIST v1.1 was defined as the time from initial occurrence of documented CR or PR until documented disease progression as determined by the investigator, or death, whichever occurred first. Duration of response was analyzed for the subset of patients who achieved an objective response. The secondary efficacy endpoint investigator-assessed PFS by RECIST v1.1 was defined as the time from the first dose of study drug to the time of PD or death from any cause during the study, whichever occurred first. The secondary efficacy endpoint OS was defined as the time from the first dose of study drug to the time of death from any cause during the study. Patients who were still alive at the time of analysis were censored at the date of their last study assessment (for active patients) or at the last date known to be alive (for patients in follow-up). The primary biomarker endpoint was to evaluate whether “positive vs. negative bTMB” could predict for improved PFS with study drug. The differences in PFS between bTMB high versus low groups at two different, primary cutoffs, 16 and 10, (i.e., bTMB ≥16 vs <16, and bTMB ≥10 vs <10) were evaluated. Gatekeeping testing procedures, with the cutoff of 16 tested first, were used to test PFS differences between the high and low groups. Secondary biomarker endpoints were the relationship between efficacy endpoints (including ORR, investigator-assessed PFS rate at 6, 9, and 12 months; and DCR), and various bTMB cutoffs, used to evaluate the correlation between clinical outcomes and various definitions of positive bTMB. For ORR, PFS, and DCR, the differences between bTMB mutation high versus low groups, measured at various cutoffs other than the primary cut off points 16 and 10 (defined as a range of 4 to 24 by twos), were evaluated.
