# Supplementary material for: Blood-based tumor mutational burden as a biomarker for atezolizumab in non-small cell lung cancer: the phase 2 B-F1RST trial
Source: Nat Med. 2022 Apr 14;28(5):939–45. doi: 10.1038/s41591-022-01754-x (PMC9117143; doi:10.1038/s41591-022-01754-x)
Supplement: Supplementary file 3 — The final version of the B-F1RST trial protocol. [file 41591_2022_1754_MOESM3_ESM.pdf]

PLEASE READ CAREFULLY:

By opening the attached document you agree to the following terms of use:

- You may not use this document or the information contained herein to a regulatory authority in connection with an application for a marketing authorization or any other regulatory submission without the express written consent of Roche. Please contact global.data_sharing@roche.com

- You may not use this document or the information contained herein to identify clinical trial patients.

- You may not copy, reproduce, or make this document available in any manner that would permit a third-party to review or use the document without first agreeing to these terms of use.

To open the document, please follow the instructions below:

- Please use Adobe Acrobat software to view the document.

- On the left hand panel, open the Attachments tab (paper clip) to see the document attached *(see screenshot below for an example).*

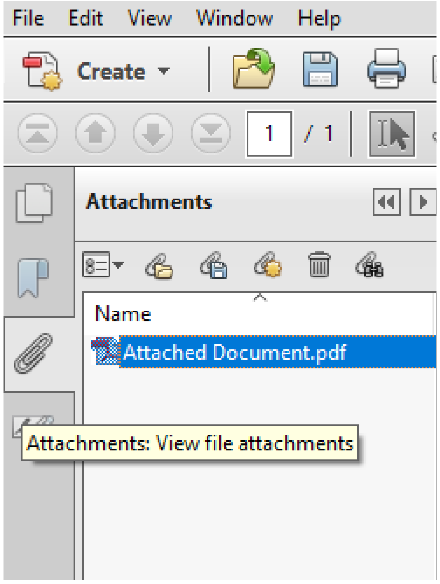

"Use of this document is governed by the terms of use on the first page of this document."

PROTOCOL

**TITLE:**	**A PHASE II SINGLE-ARM STUDY OF ATEZOLIZUMAB MONOTHERAPY IN LOCALLY ADVANCED OR METASTATIC NON–SMALL CELL LUNG CANCER: CLINICAL EVALUATION OF NOVEL BLOOD-BASED DIAGNOSTICS**
**PROTOCOL NUMBER:**	ML39237
**VERSION NUMBER:**	5
**EUDRACT NUMBER:**	Not applicable
**IND NUMBER:**	117296
**TEST PRODUCT:**	Atezolizumab (RO5541267)
**MEDICAL MONITOR:**	[redacted], Pharm.D.
**SPONSOR:**	Genentech, Inc.
**DATE FINAL:**	Version 1: 29 April 2016
**DATES AMENDED**	Version 2: 16 February 2017<br>Version 3: 05 December 2017<br>Version 4: 28 March 2018<br>Version 5: See electronic date stamp below

PROTOCOL AMENDMENT APPROVAL

CONFIDENTIAL

The information contained in this document, especially any unpublished data, is the property of Genentech, Inc. (or under its control) and therefore is provided to you in confidence as an investigator, potential investigator, or consultant, for review by you, your staff, and an applicable Ethics Committee or Institutional Review Board. It is understood that this information will not be disclosed to others without written authorization from Genentech except to the extent necessary to obtain informed consent from persons to whom the drug may be administered.

"Use of this document is governed by the terms of use on the first page of this document."

PROTOCOL AMENDMENT, VERSION 5: RATIONALE

The protocol has been amended to allow patients who remain on the study and elect to rollover into the optional long-term follow-up (LTFU) portion of the study to be followed for survival data, and patients who continue to receive study drug will also be monitored for safety events. Patients on study drug will continue therapy until they have received a total of 2 years of atezolizumab treatment (32 cycles). Thereafter patients will continue to be contacted for overall survival follow-up every 3 months for the first year, then every 6 months thereafter for up to 5 years from enrollment of the first patient into the study, until the last patient dies, withdraws consent, or is lost to follow-up, whichever occurs first.

The following additional changes have been made:

- Section 1.2 has been updated with the most current FDA-approved atezolizumab prescribing information.
- Section 2 (Table 3) has been updated to remove independent review facility (IRF) assessments from study endpoints.
- In Section 3.1 language regarding using public resources to obtain survival data and details on the handling of that data was removed. Also, language was added that allows patients to receive up to a total of 2 years of atezolizumab treatment (32 cycles).
- In Section 3.2 it was clarified that collection of LTFU for patients is optional. Also, language was added to define the length of the LTFU.
- In Section 4.5.9, language regarding using public resources to obtain survival data and details on the handling of that data was removed. Also the LTFU email address was removed.
- In Section 5.3.1, adverse event reporting for patients who receive atezolizumab and continue in the LFTU portion of the study has been added. All adverse events should be reported until 30 days after the last dose of atezolizumab during this period of the study. Additionally, serious adverse events and non-serious adverse events of special interest will be reported until 90 days after the last dose of atezolizumab for these patients.
- In Section 6, language was added to clarify that the overall survival analysis will be updated once the LTFU data is available, and will be summarized in a separate report.
- In Section 6.4.2 and 6.6.2, language regarding IRF assessments have been removed.
- Appendix 1 was renamed to Appendix 1a (Schedule of Activities) and footnote “e” was revised to refer to Appendix 1b for assessments related to the LTFU.
- Appendix 1b was created to outline the assessments related to the LTFU.

"Use of this document is governed by the terms of use on the first page of this document."

Additional minor changes have been made to improve clarity and consistency, and to adhere to current protocol template standards. Substantive new information appears in italics. This amendment represents cumulative changes to the original protocol.

"Use of this document is governed by the terms of use on the first page of this document."

TABLE OF CONTENTS

PROTOCOL AMENDMENT ACCEPTANCE FORM 10

PROTOCOL SYNOPSIS 11

1. BACKGROUND 22

1.1 Background on Non–Small Cell Lung Cancer 22

1.1.1 First-Line Treatment for NSCLC without an Epidermal Growth Factor Receptor Mutation or Anaplastic Lymphoma Kinase Rearrangement...................... 22

1.1.2 Cancer Immunotherapy: Programmed Death-Ligand 1/Programmed Death-1 Inhibitors and Biomarker Selection 23

1.1.3 Diagnostic Testing for First-Line Treatment of Non–Small Cell Lung Cancer 25

1.2 Background on Atezolizumab 25

1.2.1 Summary of Nonclinical Studies 25

1.2.2 Clinical Experience with Atezolizumab 26

1.2.2.1 Clinical Safety 27

1.2.2.2 Clinical Activity 30

1.2.3 Clinical Pharmacokinetics and Immunogenicity..................... 31

1.2.4 Rationale for Atezolizumab Dosage 32

1.3 Study Rationale and Benefit–Risk Assessment..................... 33

2. OBJECTIVES AND ENDPOINTS 35

3. STUDY DESIGN 36

3.1 Description of the Study... 36

3.2 End of Study and Length of Study 39

3.3 Rationale for Study Design 39

3.3.1 Rationale for Atezolizumab Dose and Schedule.................... 39

3.3.2 Rationale for Patient Population 40

3.3.3 Rationale for Allowing Patients to Continue Atezolizumab Treatment until Loss of Clinical Benefit 41

3.3.4 Rationale for Collection of Blood Specimens......................... 41

"Use of this document is governed by the terms of use on the first page of this document."

3.3.5 Rationale for Collection of Archival and/or Fresh Tumor Specimens 42
3.3.6 Rationale for Biomarker Assessments 42

4. MATERIALS AND METHODS 43
4.1 Patients 43
4.1.1 Inclusion Criteria 43
4.1.2 Exclusion Criteria 44
4.2 Method of Treatment Assignment and Blinding 48
4.3 Study Treatment 48
4.3.1 Formulation, Packaging, and Handling 49
4.3.2 Dosage, Administration, and Compliance 49
4.3.3 Investigational Medicinal Product Accountability 51
4.3.4 Post-Trial Access to Atezolizumab 51
4.4 Concomitant Therapy 51
4.4.1 Permitted Therapy 51
4.4.2 Cautionary Therapy for Atezolizumab-Treated Patients 52
4.4.3 Prohibited Therapy 53
4.5 Study Assessments 53
4.5.1 Informed Consent Forms and Screening Log 53
4.5.2 Medical History and Demographic Data 54
4.5.3 Physical Examinations 54
4.5.4 Vital Signs 54
4.5.5 Tumor and Response Evaluations 54
4.5.6 Laboratory, Biomarker, and Other Biological Samples 55
4.5.6.1 Samples for Safety Laboratory Tests 55
4.5.6.2 Biomarker Samples 56
4.5.7 Optional Samples for Research Biosample Repository 58
4.5.7.1 Overview of the Research Biosample Repository 58
4.5.7.2 Approval by the Institutional Review Board or Ethics Committee 58
4.5.7.3 Sample Collection 58

"Use of this document is governed by the terms of use on the first page of this document."

4.5.7.4 Confidentiality ... 59
4.5.7.5 Consent to Participate in the Research Biosample Repository ... 60
4.5.7.6 Withdrawal from the Research Biosample Repository ... 60
4.5.7.7 Monitoring and Oversight ... 60
4.5.8 On-Study Survival Follow-Up ... 61
4.5.9 Optional Long-Term Follow-Up ... 61
4.5.10 ECOG Performance Status ... 61
4.6 Patient, Treatment, Study, and Site Discontinuation ... 61
4.6.1 Patient Discontinuation ... 61
4.6.2 Study Treatment Discontinuation ... 62
4.6.3 Study and Site Discontinuation ... 62
5. ASSESSMENT OF SAFETY ... 63
5.1 Safety Plan ... 63
5.1.1 Risks Associated with Atezolizumab ... 63
5.1.2 Management of Patients Who Experience Specific Adverse Events ... 64
5.2 Safety Parameters and Definitions ... 65
5.2.1 Adverse Events ... 65
5.2.2 Serious Adverse Events (Immediately Reportable to the Sponsor) ... 65
5.2.3 Adverse Events of Special Interest (Immediately Reportable to the Sponsor) ... 66
5.3 Methods and Timing for Capturing and Assessing Safety Parameters ... 67
5.3.1 Adverse Event Reporting Period ... 67
5.3.2 Eliciting Adverse Event Information ... 68
5.3.3 Assessment of Severity of Adverse Events ... 68
5.3.4 Assessment of Causality of Adverse Events ... 69
5.3.5 Procedures for Recording Adverse Events ... 70
5.3.5.1 Infusion-Related Reactions ... 70
5.3.5.2 Diagnosis versus Signs and Symptoms ... 70

"Use of this document is governed by the terms of use on the first page of this document."

5.3.5.3	Adverse Events That Are Secondary to Other Events	71
5.3.5.4	Persistent or Recurrent Adverse Events	71
5.3.5.5	Abnormal Laboratory Values	72
5.3.5.6	Abnormal Vital Sign Values	72
5.3.5.7	Abnormal Liver Function Tests	73
5.3.5.8	Deaths	73
5.3.5.9	Preexisting Medical Conditions	74
5.3.5.10	Lack of Efficacy or Worsening of NSCLC	74
5.3.5.11	Hospitalization or Prolonged Hospitalization	74
5.3.5.12	Adverse Events Associated with an Overdose or Error in Drug Administration	75
5.4	Immediate Reporting Requirements from Investigator to Sponsor	75
5.4.1	Emergency Medical Contacts	76
5.4.2	Reporting Requirements for Serious Adverse Events and Adverse Events of Special Interest	76
5.4.2.1	Events That Occur prior to Study Drug Initiation	76
5.4.2.2	Events That Occur after Study Drug Initiation	76
5.4.3	Reporting Requirements for Pregnancies	77
5.4.3.1	Pregnancies in Female Patients	77
5.4.3.2	Congenital Anomalies/Birth Defects and Abortions	77
5.4.4	Reporting Requirements for Cases of Atezolizumab Accidental Overdose or Medication Error	77
5.5	Follow-Up of Patients after Adverse Events	78
5.5.1	Investigator Follow-Up	78
5.5.2	Sponsor Follow-Up	79
5.6	Adverse Events That Occur after the Adverse Event Reporting Period	79
5.7	Expedited Reporting to Health Authorities, Investigators, Institutional Review Boards, and Ethics Committees	79

"Use of this document is governed by the terms of use on the first page of this document."

6. STATISTICAL CONSIDERATIONS AND ANALYSIS PLAN 80
6.1 Determination of Sample Size 80
6.2 Summaries of Conduct of Study 80
6.3 Summaries of Demographic and Baseline Characteristics 81
6.4 Efficacy Analyses 81
6.4.1 Primary Efficacy Endpoint.. 81
6.4.2 Secondary Efficacy Endpoints 81
6.4.3 Exploratory Efficacy Endpoints 82
6.5 Safety Analyses 82
6.6 Biomarker Analyses 82
6.6.1 Primary Biomarker Analyses 82
6.6.2 Secondary Biomarker Analyses 82
6.6.3 Exploratory Biomarker Analyses... 83
6.7 Interim Analyses 83
6.7.1 Planned Interim Analysis 83
6.7.2 Optional Interim Analyses 83
7. DATA COLLECTION AND MANAGEMENT 83
7.1 Data Quality Assurance 83
7.2 Electronic Case Report Forms... 84
7.3 Source Data Documentation.. 84
7.4 Use of Computerized Systems 85
7.5 Retention of Records 85
8. ETHICAL CONSIDERATIONS... 85
8.1 Compliance with Laws and Regulations 85
8.2 Informed Consent 86
8.3 Institutional Review Board or Ethics Committee 87
8.4 Confidentiality 87
8.5 Financial Disclosure 88
9. STUDY DOCUMENTATION, MONITORING, AND ADMINISTRATION 88
9.1 Study Documentation 88
9.2 Protocol Deviations.. 88

"Use of this document is governed by the terms of use on the first page of this document."

9.3 Site Inspections 88
9.4 Administrative Structure 88
9.5 Publication of Data and Protection of Trade Secrets 89
9.6 Protocol Amendments 90

10. REFERENCES 91

LIST OF TABLES

Table 1 Adverse Events Reported in ≥ 10% of Patients in Study PCD4989g 28
Table 2 Treatment-Related Adverse Events Reported in at Least 10% of Patients in Either Treatment Arm in Study GO28753 (POPLAR) 29
Table 3 Objectives and Corresponding Endpoints 35
Table 4 Administration of First and Subsequent Infusions of Atezolizumab 50
Table 5 Adverse Event Severity Grading Scale for Events Not Specifically Listed in the NCI CTCAE 69
Table 6 Causal Attribution Guidance 70

LIST OF FIGURES

Figure 1 Study Schema 37

LIST OF APPENDICES

Appendix 1*a* Schedule of Activities 94
Appendix 1b *Optional Long-Term Follow-Up Schedule of Assessments* 99
Appendix 2 Schedule of Biomarker Samples 100
Appendix 3 IASLC Lung Cancer Staging Project Proposed for the Eighth Edition of the American Joint Committee on Cancer Non–Small Cell Lung Cancer Staging System[1] 101
Appendix 4 Response Evaluation Criteria in Solid Tumors (RECIST): Excerpt from Original Publication 103
Appendix 5 Anaphylaxis Precautions 115
Appendix 6 Eastern Cooperative Oncology Group Performance Status Scale 116
Appendix 7 Preexisting Autoimmune Diseases 117
Appendix 8 Management of Atezolizumab-Specific Adverse Events 118

"Use of this document is governed by the terms of use on the first page of this document."

PROTOCOL AMENDMENT ACCEPTANCE FORM

**TITLE:**	**A PHASE II SINGLE-ARM STUDY OF ATEZOLIZUMAB MONOTHERAPY IN LOCALLY ADVANCED OR METASTATIC NON–SMALL CELL LUNG CANCER: CLINICAL EVALUATION OF NOVEL BLOOD-BASED DIAGNOSTICS**
**PROTOCOL NUMBER:**	ML39237
**VERSION NUMBER:**	5
**EUDRACT NUMBER:**	Not applicable
**IND NUMBER:**	117296
**TEST PRODUCT:**	Atezolizumab (RO5541267)
**MEDICAL MONITOR:**	[redacted], Pharm.D.
**SPONSOR:**	Genentech, Inc.

I agree to conduct the study in accordance with the current protocol.

____

Principal Investigator's Name (print)

____ ________________

Principal Investigator's Signature Date

Please retain the signed original of this form for your study files. Please return a copy as instructed by your local study monitor.

"Use of this document is governed by the terms of use on the first page of this document."

PROTOCOL SYNOPSIS

**TITLE:**	**A PHASE II SINGLE-ARM STUDY OF ATEZOLIZUMAB MONOTHERAPY IN LOCALLY ADVANCED OR METASTATIC NON–SMALL CELL LUNG CANCER: CLINICAL EVALUATION OF NOVEL BLOOD-BASED DIAGNOSTICS**
**PROTOCOL NUMBER:**	ML39237
**VERSION NUMBER:**	5
**EUDRACT NUMBER:**	Not applicable
**IND NUMBER:**	117296
**TEST PRODUCT:**	Atezolizumab (RO5541267)
**PHASE:**	II
**INDICATION:**	Non–Small Cell Lung Cancer
**SPONSOR:**	Genentech, Inc.

Objectives and Endpoints

The aim of this study is to evaluate the clinical efficacy of atezolizumab as a single-agent, first-line therapy in patients with immunotherapy–naive, Stage IIIB–IVB NSCLC. Diagnostic selection of patients will not be a criterion for enrollment.

The primary biomarker objective is to measure tumor mutational burden from blood (bTMB) and to evaluate whether it can predict for improved clinical outcomes with atezolizumab. Clinical outcome, as measured by progression-free survival (PFS), will be correlated to bTMB retrospectively to define the threshold for positivity. Specific objectives and corresponding endpoints for the study are outlined below.

Objectives	Corresponding Endpoints
**Primary Efficacy Objective:**	
• To evaluate the clinical efficacy of atezolizumab	• INV-assessed ORR by RECIST v1.1
**Secondary Efficacy Objective:**	
• To evaluate the efficacy of atezolizumab	• *INV*-assessed ORR by RECIST v1.1<br>• INV-assessed PFS by RECIST v1.1<br>• INV-assessed duration of response by RECIST v1.1<br>• Overall survival
**Safety Objective:**	
• To evaluate the safety and tolerability of atezolizumab	• Incidence of adverse events, with severity determined through use of NCI CTCAE v4.0
**Primary Biomarker Objective:**	
• To evaluate whether "positive vs. negative bTMB" defined at various bTMB quantiles	• Relationship between INV-assessed PFS and various bTMB quantiles

"Use of this document is governed by the terms of use on the first page of this document."

Objectives	Corresponding Endpoints
can predict for improved PFS with atezolizumab	
**Secondary Biomarker Objective:**	
• To evaluate the correlation between clinical outcomes including but not limited to PFS, PFS rate at 6, 9, and 12 months, ORR, and various definitions of positive bTMB	• Relationship between efficacy endpoints including INV-assessed PFS rate at 6, 9, and 12 months, ORR and various bTMB quantiles and efficacy
**Exploratory Biomarker Objectives in Blood:**	
• To evaluate whether higher expression of an immune-related gene signature in blood PBMCs predicts for improved PFS with atezolizumab	• Relationship between PFS and expression of an immune gene signature in PBMCs
• To assess whether bTMB is altered as a result of treatment with immunotherapy	• Relationship between bTMB and treatment with immunotherapy
• To assess the status of additional circulating biomarkers related to immunotherapy and NSCLC and outcomes with atezolizumab	• Relationship between circulating biomarkers related to immunotherapy and NSCLC and efficacy
**Exploratory Biomarker Objective in Tissue:**	
• To perform retrospective tTMB analysis and NGS-based mutation testing on tissue biopsies for patients providing specific consent and viable samples	• Relationship between somatic mutations and tTMB on efficacy. All mutations will be identified through NGS performed on DNA extracted from tumor tissue.

bTMB = blood tumor mutational burden; INV = investigator; NGS = next-generation sequencing; NCI CTCAE = National Cancer Institute Common Terminology Criteria for Adverse Events; NSCLC = non–small cell lung cancer; ORR = objective response rate; PBMC = peripheral blood mononuclear cell; PFS = progression-free survival; RECIST = Response Evaluation Criteria in Solid Tumors; tTMB = tissue tumor mutational burden.

Study Design

Description of Study

This is a Phase II, open-label, prospective, multicenter study designed to evaluate the efficacy and safety of single-agent atezolizumab as a first-line therapy in patients with locally advanced or metastatic NSCLC. In addition, the primary biomarker objective is to measure bTMB and evaluate whether it can predict for improved clinical outcome with atezolizumab.

There will be approximately 150 patients with Stage IIIB–IVB, locally advanced or metastatic, EGFR/ALK–negative NSCLC who are immunotherapy-naive enrolled at approximately 25–30 study sites in the United States.

Atezolizumab will be administered on Day 1 of each 21-day cycle. Patients who meet the Response Evaluation Criteria in Solid Tumors (RECIST) v1.1 criteria for progressive disease at any timepoint during treatment will be permitted to continue atezolizumab treatment if there is evidence of clinical benefit, defined as meeting all of the following criteria:

- Evidence of clinical benefit as assessed by the investigator
- Absence of symptoms and signs (including worsening of laboratory values [e.g., new or worsening hypercalcemia]) indicating unequivocal progression of disease
- No decline in Eastern Cooperative Oncology Group (ECOG) performance status that can be attributed to disease progression
- Absence of tumor progression at critical anatomical sites (e.g., leptomeningeal disease) that cannot be managed by protocol-allowed medical interventions

"Use of this document is governed by the terms of use on the first page of this document."

Patients for whom approved therapies exist must provide written consent to acknowledge deferring these treatment options in favor of continuing study drug at the time of initial progression.

Screening tests and evaluations will be performed within 28 days prior to treatment initiation.

Blood samples will be collected at screening, baseline, during therapy, and at first evidence of radiographic disease progression or loss of clinical benefit. Data from the latter blood draws will be used to explore whether the radiographic findings are consistent with changes in bTMB as measured by the blood-based assays and to explore possible mutational mechanisms of resistance. Additional blood samples may be taken at each scan as defined in the schedule of activities.

Tissue biopsies (tissue blocks preferred) may be submitted at any time during the study, with biopsies from diagnosis (archival or fresh) and at progression preferred. For patients who provide tissue samples, next generation sequencing (NGS) may be performed by Foundation Medicine. If performed by Foundation Medicine, the investigator can obtain results from these analyses in the form of an NGS report, which is available upon request directly from Foundation Medicine. The investigator may share and discuss the results with the patient, unless the patient chooses otherwise. The Foundation Medicine NGS assay, FoundationOne®, has not been cleared or approved by health authorities. The NGS report is generated for research purposes and is not provided for the purpose of guiding future treatment decisions. Results may not be available for samples that do not meet testing criteria. Patients who are unable to undergo biopsy sample collection but otherwise meet the eligibility criteria may still be enrolled to receive atezolizumab.

All patients will undergo tumor assessment at baseline and every 6 weeks (± 7 days) thereafter regardless of dose delays for the first 12 months following Cycle 1, Day 1. After 12 months, tumor assessment will be required every 9 weeks (± 7 days); tumor assessments will continue until disease progression per RECIST v1.1 or loss of clinical benefit, consent withdrawal, study discontinuation, study completion or death, whichever occurs first. Patients who discontinue treatment for reasons other than disease progression (e.g., toxicity) will continue scheduled tumor assessments until disease progression, withdrawal of consent, study discontinuation, study completion (i.e., end of study), or death, whichever occurs first. In the absence of disease progression, tumor assessments should continue regardless of whether patients start a new anti-cancer therapy, unless consent is withdrawn.

All patients will be followed for survival and anti-cancer therapy while in the study and will be given the option of participating in a long-term follow-up *which will include collection of long-term survival data beyond the end of the study*. The optional long-term follow-up will begin following the end of the study. Patients must provide additional consent to participate in this optional long-term follow-up. *Patients on study drug will continue therapy until they have received a total of 2 years of atezolizumab treatment (32 cycles). Thereafter, patients will continue overall survival follow-up every 3 months for the first year, then every 6 months thereafter, for up to 5 years from enrollment of the first patient into the study, until the last patient dies, withdraws consent, or is lost to follow-up, whichever occurs first*.

Safety will be assessed on the basis of vital sign measurements, ECOG performance status scores, physical examination findings, clinical laboratory test results, and the frequency, severity, and relationship to atezolizumab of adverse events.

When a patient discontinues study drug, regardless of the reason for discontinuation, the patient will be asked to return to the clinic within 30 days after the last dose for a study drug discontinuation visit. Patients who discontinue will not be replaced.

Number of Patients

There will be approximately 150 patients with Stage IIIB–IVB, locally advanced or metastatic, epidermal growth factor receptor (EGFR)/anaplastic lymphoma kinase (ALK)–negative NSCLC who are immunotherapy-naive enrolled at approximately 25-30 study sites in the United States.

Target Population

Inclusion Criteria

Patients must meet the following criteria for study entry:

- Signed Informed Consent Form

"Use of this document is governed by the terms of use on the first page of this document."

- Age ≥18 years
- ECOG performance status of 0 or 1
- Histologically or cytologically confirmed Stage IIIB–IVB NSCLC (based on the IASLC Lung Cancer Staging Project proposed for the eighth edition of the AJCC NSCLC staging system)
    - Patients with histologically or cytologically confirmed Stage IIIB-IV NSCLC based on the seventh edition of the AJCC NSCLC staging system are also eligible for study entry.
- For patients who have received prior neo-adjuvant/adjuvant chemotherapy or chemoradiotherapy with curative intent for non-metastatic disease: a treatment-free interval of at least 6 months prior to enrollment.
- Patients with any PD-L1 test result by IHC are eligible for the study.
- Patients without a PD-L1 test result are eligible for the study.
- Measurable disease, as defined by RECIST v1.1, based on protocol-specified tumor assessments.
    - Previously irradiated lesions can only be considered measurable disease if disease progression has been unequivocally documented at that site since radiation and the previously irradiated lesion is not the only site of measureable disease
- Adequate hematologic and end-organ function, defined by the following laboratory test results obtained within 14 days prior to the first dose of study drug:
    - ANC ≥1500 cells/μL without granulocyte colony-stimulating factor support
    - Lymphocyte count ≥ 500 cells/μL
    - Platelet count ≥ 100,000 cells/μL without transfusion
    - Hemoglobin ≥ 9.0 g/dL

      Patients may be transfused to meet this criterion
    - INR or activated partial thromboplastin time (aPTT) ≤ 1.5 × the upper limit of normal (ULN)

      This applies only to patients who are not receiving therapeutic anticoagulation; patients receiving therapeutic anticoagulation must have an INR or aPTT within therapeutic limits for at least 1 week prior to enrollment.
    - AST, ALT, and alkaline phosphatase ≤2.5 × ULN with the following exceptions:

      Patients with documented liver metastases: AST and/or ALT ≤ 5 × ULN

      Patients with documented liver or bone metastases: alkaline phosphatase ≤ 5 × ULN
    - Serum bilirubin ≤1.5 × ULN

      Patients with known Gilbert disease who have a serum bilirubin level ≤ 3 × ULN may be enrolled.
    - Calculated creatinine clearance ≥ 30 mL/min
- For women of childbearing potential: agreement to remain abstinent (refrain from heterosexual intercourse) or use a contraceptive method with a failure rate of <1% per year during the treatment period and for at least 5 months after the last dose of atezolizumab.
    - A woman is considered to be of childbearing potential if she is postmenarcheal, has not reached a postmenopausal state (≥ 12 continuous months of amenorrhea with no identified cause other than menopause), and has not undergone surgical sterilization (removal of ovaries and/or uterus).
    - Examples of contraceptive methods with a failure rate of < 1% per year include bilateral tubal ligation, male sterilization, hormonal contraceptives that inhibit ovulation, hormone-releasing intrauterine devices, and copper intrauterine devices.
    - The reliability of sexual abstinence should be evaluated in relation to the duration of the clinical study and the preferred and usual lifestyle of the patient. Periodic abstinence (e.g., calendar, ovulation, symptothermal, or postovulation methods) and withdrawal are not acceptable methods of contraception.

"Use of this document is governed by the terms of use on the first page of this document."

Exclusion Criteria

Patients who meet any of the following criteria will be excluded from study entry:

- Prior treatment with immunotherapy for any stage NSCLC, including early-stage (neoadjuvant or adjuvant) disease.
- Patients with EGFR-sensitizing mutations and ALK rearrangements are excluded from the study and should be treated according to standard clinical guidelines. Refer to National Comprehensive Cancer Network (NCCN) guidelines for recommendations on molecular testing (NCCN Guidelines 2016).
- Active CNS metastases requiring treatment as determined by computed tomography (CT) or magnetic resonance imaging (MRI) evaluation during screening and prior radiographic assessments
  - Patients with a history of treated asymptomatic CNS metastases are eligible, provided they meet all of the following criteria:

    Only supratentorial and cerebellar metastases allowed (i.e., no metastases to midbrain, pons, medulla, or spinal cord)

    No ongoing requirement for corticosteroids as therapy for CNS disease

    No stereotactic radiation within 7 days or whole-brain radiation within 14 days prior to enrollment

    No evidence of interim progression between the completion of CNS-directed therapy and the screening radiographic study
  - Patients with new asymptomatic CNS metastases detected at the screening scan must receive radiation therapy and/or surgery for CNS metastases. Following treatment, these patients may then be eligible without the need for an additional brain scan prior to enrollment, if all other criteria are met.
- Spinal cord compression not definitively treated with surgery and/or radiation, or previously diagnosed and treated spinal cord compression without evidence that disease has been clinically stable for ≥ 2 weeks prior to enrollment
- Leptomeningeal disease
- Uncontrolled tumor-related pain
  - Patients requiring pain medication must be on a stable regimen at study entry.
  - Symptomatic lesions amenable to palliative radiotherapy (e.g., bone metastases or metastases causing nerve impingement) should be treated prior to enrollment. Patients should be recovered from the effects of radiation. There is no required minimum recovery period.
  - Asymptomatic metastatic lesions whose further growth would likely cause functional deficits or intractable pain (e.g., epidural metastasis that is not currently associated with spinal cord compression) should be considered for loco-regional therapy if appropriate prior to enrollment.
- Uncontrolled pleural effusion, pericardial effusion, or ascites requiring recurrent drainage procedures (once monthly or more frequently)
  - Patients with indwelling catheters (e.g., PleurX®) are allowed.
- Uncontrolled or symptomatic hypercalcemia ($>$1.5 mmol/L ionized calcium or calcium $>$12 mg/dL or corrected serum calcium $>$ULN)
  - Patients who are receiving denosumab prior to enrollment must be willing and eligible to discontinue its use and replace it with a bisphosphonate instead while in the study.
- Malignancies other than NSCLC within 5 years prior to enrollment, with the exception of those with a negligible risk of metastasis or death (e.g., expected 5-year overall survival [OS] $>$ 90%) treated with an expected curative outcome (such as adequately treated carcinoma in situ of the cervix, basal- or squamous-cell skin cancer, localized prostate cancer treated surgically with curative intent, or ductal carcinoma in situ treated surgically with curative intent)
- Women who are pregnant, lactating, or intending to become pregnant during the study

"Use of this document is governed by the terms of use on the first page of this document."

- Women who are not postmenopausal (≥ 12 months of non–therapy-induced amenorrhea) or surgically sterile must have a negative serum pregnancy test result within 14 days prior to initiation of study drug.

- History of severe allergic, anaphylactic, or other hypersensitivity reactions to chimeric or humanized antibodies or fusion proteins
- Known hypersensitivity to biopharmaceuticals produced in Chinese hamster ovary cells or any component of the atezolizumab formulation
- History of autoimmune disease, including but not limited to myasthenia gravis, myositis, autoimmune hepatitis, systemic lupus erythematosus, rheumatoid arthritis, inflammatory bowel disease, vascular thrombosis associated with antiphospholipid syndrome, Wegener's granulomatosis, Sjögren's syndrome, Guillain-Barré syndrome, multiple sclerosis, vasculitis, or glomerulonephritis
  - Patients with a history of autoimmune-related hypothyroidism on a stable dose of thyroid-replacement hormone may be eligible for this study.
  - Patients with controlled Type 1 diabetes mellitus on a stable insulin regimen are eligible for this study.
  - Patients with eczema, psoriasis, lichen simplex chronicus, or vitiligo with dermatologic manifestations only (e.g., patients with psoriatic arthritis would be excluded) are permitted provided that they meet the following conditions:
    - Rash must cover <10% of body surface area
    - Disease is well controlled at baseline and only requiring low-potency topical steroids
    - No acute exacerbations of underlying condition within the last 12 months requiring treatment with either psoralen plus ultraviolet A radiation, methotrexate, retinoids, biologic agents, oral calcineurin inhibitors, or high-potency or oral steroids.
- History of idiopathic pulmonary fibrosis, organizing pneumonia (e.g., bronchiolitis obliterans), drug-induced pneumonitis, idiopathic pneumonitis, or evidence of active pneumonitis on screening chest CT scan
  - History of radiation pneumonitis in the radiation field (fibrosis) is permitted.
- Positive HIV test
- Active hepatitis B (HBV) infection (chronic or acute), defined as having a positive hepatitis B surface antigen (HBsAg) test at screening
  - Patients with past or resolved HBV infection, defined as having a negative HBsAg test and a positive total hepatitis B core antibody (HBcAb) test, are eligible for the study. An HBV DNA test must be performed in these patients prior to enrollment.
- Active hepatitis C virus (HCV) infection, defined as having a positive HCV antibody test and a positive HCV RNA test at screening
- Active tuberculosis
- Severe infections within 4 weeks prior to enrollment, including but not limited to hospitalization for complications of infection, bacteremia, or severe pneumonia
- Significant cardiovascular disease, such as New York Heart Association cardiac disease (Class II or greater), myocardial infarction within 3 months prior to enrollment, unstable arrhythmias, or unstable angina
  - Patients with known coronary artery disease, congestive heart failure not meeting the above criteria, or left ventricular ejection fraction <50% must be on a stable medical regimen that is optimized in the opinion of the treating physician, in consultation with a cardiologist if appropriate.
- Major surgical procedure other than for diagnosis within 28 days prior to enrollment or anticipation of need for a major surgical procedure during the course of the study
- Prior allogeneic bone marrow transplantation or solid organ transplant

"Use of this document is governed by the terms of use on the first page of this document."

- Any other diseases, metabolic dysfunction, physical examination finding, or clinical laboratory finding giving reasonable suspicion of a disease or condition that contraindicates the use of an investigational drug or that may affect the interpretation of the results or render the patient at high risk from treatment complications
- Prior treatment with CD137 agonists or immune checkpoint blockade therapies, anti−PD-1, and anti−PD-L1 therapeutic antibodies
- Treatment with systemic immunostimulatory agents (including but not limited to interferons or interleukin 2) within 4 weeks or 5 half-lives of the drug, whichever is longer, prior to enrollment
- Any approved anti-cancer therapy or hormonal therapy within 3 weeks prior to initiation of study drug; the following exceptions are allowed:
  - Hormone-replacement therapy or oral contraceptives
  - Tyrosine kinase inhibitors (TKIs) approved for treatment of NSCLC discontinued ≥7 days prior to enrollment; the baseline scan must be obtained after discontinuation of prior TKIs.
- Treatment with any other investigational agent or participation in another clinical study with therapeutic intent within 28 days prior to enrollment
- Received therapeutic oral or intravenous (IV) antibiotics within 2 weeks prior to enrollment
  - Patients receiving prophylactic antibiotics (e.g., for prevention of a urinary tract infection or to prevent chronic obstructive pulmonary disease exacerbation) are eligible.
- Administration of a live, attenuated vaccine within 4 weeks before Cycle 1, Day 1 or anticipation that such a live attenuated vaccine will be required during the study or for 5 months after the last dose of atezolizumab
  - Influenza vaccination should be given during influenza season only (approximately October to March).
  - Patients must not receive live, attenuated influenza vaccine (e.g., FluMist®) within 4 weeks prior to Cycle 1, Day 1, at any time during the study, or for 5 months after the last dose of atezolizumab.
- Treatment with systemic corticosteroids or other systemic immunosuppressive medications (including but not limited to prednisone, dexamethasone, cyclophosphamide, azathioprine, methotrexate, thalidomide, and anti−tumor necrosis factor [TNF] agents) within 2 weeks prior to enrollment
  - Patients who have received acute, low-dose, systemic immunosuppressant medications (e.g., a one-time dose of dexamethasone for nausea) may be enrolled in the study after discussion with and approval by the Medical Monitor.
  - The use of inhaled corticosteroids for chronic obstructive pulmonary disease, mineralocorticoids (e.g., fludrocortisone) for patients with orthostatic hypotension, and low-dose supplemental corticosteroids for adrenocortical insufficiency are allowed.

End of Study

The end of this study is defined as the date after which all enrolled patients who remain alive have been followed for at least 18 months from study-drug initiation. Thereafter, patients *who discontinue study drug* will be followed for *optional* long-term survival.

Patients who remain on study drug will continue therapy until they have received a total of 2 years of atezolizumab treatment (32 cycles), and will be followed for safety. Survival data will be collected during the optional long-term follow up period every 3 months for the first year, then every 6 months thereafter, for up to 5 years from enrollment of the first patient into the study until the last patient dies, withdraws consent, or is lost to follow-up, whichever occurs first.

Length of Study

The total length of the study, from screening of the first patient to the end of the study, is expected to be approximately 3 years. *In addition, the Sponsor may decide to terminate the study at any time.*

"Use of this document is governed by the terms of use on the first page of this document."

Investigational Medicinal Products

The investigational medicinal product for this study is atezolizumab.

Test Product (Investigational Drug)

Patients will receive 1200 mg of atezolizumab (equivalent to an average body weight–based dose of 15 mg/kg) administered by IV infusion every 21 ($\pm$3) days. Administration of atezolizumab will be performed in a setting with emergency medical facilities and staff who are trained to monitor for and respond to medical emergencies.

Non-Investigational Medicinal Products

Premedication with antihistamines may be administered for any atezolizumab infusions after Cycle 1. Patients who use oral contraceptives, hormone-replacement therapy, or other maintenance therapy should continue their use. In general, investigators should manage a patient's care with supportive therapies as clinically indicated per local standards.

Systemic corticosteroids and TNF-α inhibitors may attenuate potential beneficial immunologic effects of treatment with atezolizumab. Therefore, in situations where systemic corticosteroids or TNF-α inhibitors would be routinely administered, alternatives, including antihistamines, should be considered first by the treating physician. If the alternatives are not feasible, systemic corticosteroids and TNF-α inhibitors may be administered at the discretion of the treating physician except in the case of patients for whom CT scans with contrast are contraindicated (i.e., patients with contrast allergy or impaired renal clearance).

Systemic corticosteroids are recommended, with caution at the discretion of the treating physician, for the treatment of specific adverse events when associated with atezolizumab therapy.

Statistical Methods

Primary Efficacy Analyses

The efficacy analysis population will be based on all patients who have received at least one dose of study drug. The primary efficacy endpoint of this study is investigator-assessed objective response rate (ORR), defined as the proportion of patients whose confirmed best overall response is either a partial response (PR) or complete response (CR) per RECIST v1.1. For this analysis, patients not meeting these criteria, including patients without at least one post-baseline response assessment, will be considered non-responders. An estimate of the ORR from all patients who received study drug and the 95% confidence interval (CI) will be calculated by using the Blaker method.

Primary Biomarker Analyses

Biomarker analysis population will be based on all patients who have received at least one dose of study drug and have baseline biomarker assessment. Kaplan-Meier (K-M) curves and a log-rank test will be used to evaluate the differences in investigator-assessed PFS between mutation positive versus negative groups at various cutoff points. Tests will be two-sided at a significance level of 0.10.

Determination of Sample Size

The sample size for this study is based on the primary efficacy objective and primary biomarker objective. Based on 150 patients, the maximum half width of the 2-sided 95% CI of the estimated ORR will be within 8%. Additionally, based on 120 biomarker-evaluable patients (80% of the study population), with a minimum follow-up of 6 months during the primary analysis, and with 28 patients in the smaller biomarker positive or negative group (19% of the study population), the study primary biomarker analysis will have 89% and 79% power to detect PFS difference if the hazard ratios of PFS between the biomarker positive vs. negative group is 0.50 or 0.55, respectively. The above-mentioned power is for a 2-sided test with a significance level of 0.1, assuming a median PFS of 4 months for the subgroup with a shorter median PFS.

Interim Analyses

One interim analysis is planned 6 months after approximately one-half of the patients have been enrolled. The purpose of the interim analysis is to provide preliminary results in evaluating the biomarker cutoff and to perform a futility analysis. No conclusions or study alterations/terminations are to be made based on the biomarker interim analysis results. No type-1 error adjustments will be made for the interim analysis.

"Use of this document is governed by the terms of use on the first page of this document."

Given the hypothesis-generating nature of this study, the Sponsor may choose to conduct additional interim analyses. The decision to conduct an optional interim analysis and the timing of the analysis will be documented in the Sponsor's trial master file prior to the conduct of the interim analysis.

"Use of this document is governed by the terms of use on the first page of this document."

LIST OF ABBREVIATIONS AND DEFINITIONS OF TERMS

Abbreviation	Definition
AJCC	American Joint Committee on Cancer
ALK	anaplastic lymphoma kinase
aPTT	activated partial thromboplastin time
ATA	anti-therapeutic antibody
BSC	best supportive care
bTMB	blood tumor mutational burden
CR	complete response
CRO	contract research organization
CT	computed tomography
CTCAE	Common Terminology Criteria for Adverse Events
ctDNA	circulating tumor DNA
C_{trough}	trough concentration
DOR	duration of response
EC	ethics committee
ECOG	Eastern Cooperative Oncology Group
EGFR	epidermal growth factor receptor
eCRF	electronic Case Report Form
EDC	electronic data capture
FDA	U.S. Food and Drug Administration
HBcAb	hepatitis B core antibody
HBsAg	hepatitis B surface antigen
HBV	hepatitis B virus
HCV	hepatitis C virus
HIPAA	Health Insurance Portability and Accountability Act
HR	hazard ratio
IASLC	International Association for the Study of Lung Cancer
ICH	International Conference on Harmonisation
IgG1	immunoglobulin G subclass 1
IMP	investigational medicinal product
IND	Investigational New Drug (application)
INR	International normalized ratio
ITT	intent-to-treat
IRB	Institutional Review Board
IV	intravenous
IxRS	interactive voice/Web response system

"Use of this document is governed by the terms of use on the first page of this document."

Abbreviation	Definition
K-M	Kaplan-Meier
KRAS	Kirsten rat sarcoma
MRI	magnetic resonance imaging
MTD	maximum tolerated dose
NaF-PET	sodium fluoride positron emission tomography
NCCN	National Comprehensive Cancer Network
NCI	National Cancer Institute
NGS	next-generation sequencing
NSCLC	non–small cell lung cancer
ORR	objective response rate
OS	overall survival
PBMC	peripheral blood mononuclear cell
PCR	polymerase chain reaction
PD	progressive disease
PD-1	programmed death-1
PD-L1	programmed death-ligand 1
PFS	progression-free survival
PK	pharmacokinetic
PR	partial response
q21d	every 21 days
RBR	Research Biosample Repository
RCC	renal cell carcinoma
RECIST	Response Evaluation Criteria in Solid Tumors
TKI	tyrosine kinase inhibitor
TNF	tumor necrosis factor
tTMB	tissue tumor mutational burden
UC	urothelial cancer
ULN	upper limit of normal
WES	whole exome sequencing
WGS	whole genome sequencing

"Use of this document is governed by the terms of use on the first page of this document."

1. BACKGROUND

1.1 BACKGROUND ON NON–SMALL CELL LUNG CANCER

Lung cancer remains the leading cause of cancer deaths worldwide and is the most common cancer in both men and women, accounting for approximately 224,390 of all new cases and nearly 160,000 deaths in 2016 in the United States (Miller et al. 2016; SEER Stat Fact Sheet 2016). Non–small cell lung cancer (NSCLC) is the predominant subtype of lung cancer, accounting for approximately 85% of all cases (Molina et al. 2008; Howlader et al. 2014). NSCLC can be divided into two major histologic types: adenocarcinoma and squamous cell carcinoma (Travis et al. 2011). Adenocarcinoma histology accounts for more than one-half of all NSCLC, whereas squamous cell histology accounts for approximately 25% (Langer et al. 2010) of NSCLC. The remaining cases of NSCLC are represented by large cell carcinoma, neuroendocrine tumors, sarcomatoid carcinoma, and poorly differentiated histology.

The overall 5-year survival rate for advanced disease is 2%–4%, depending on geographic location (Cetin et al. 2011). Poor prognostic factors for survival in patients with NSCLC include advanced stage of disease, poor performance status, a history of smoking, and a history of unintentional weight loss at the time of initial diagnosis. More than one-half of the patients with NSCLC are diagnosed with distant metastases, which directly contribute to poor survival outcomes.

1.1.1 First-Line Treatment for NSCLC without an Epidermal Growth Factor Receptor Mutation or Anaplastic Lymphoma Kinase Rearrangement

Genomic profiling of tumors has enabled a deeper understanding of the underlying biology and molecular mechanisms of lung cancer. These efforts have revolutionized first-line treatment of NSCLC, specifically in patients with actionable driver mutations such as epidermal growth factor receptor (EGFR) mutations and gene rearrangements in anaplastic lymphoma kinase (ALK) (Tsui et al. 2016). Targeted treatment of these “driver” mutations includes the use of tyrosine kinase inhibitors (TKIs) such as erlotinib and alectinib, which confer objective response rates (ORRs) upwards of 60%–70% (Thomas et al. 2015). Although these treatments have become the mainstay of first-line treatment in patients with these molecular subsets, the frequency of these mutations (approximately 25%) (Shtivelman et al. 2014), and thus the proportion of patients benefiting from such therapy, remains modest.

For patients with locally advanced and metastatic NSCLC not harboring an activating EGFR mutation or ALK gene rearrangement, platinum-based doublet chemotherapy remains as a first-line therapy and standard of care for NSCLC. A number of clinical trials have attempted to identify the optimal doublet for advanced NSCLC. In 2002, results from a Phase III randomized trial comparing four different platinum-based doublets were published (Schiller et al, 2002). In this study of 1155 patients with advanced NSCLC, no differences in overall survival (OS) were observed among those

"Use of this document is governed by the terms of use on the first page of this document."

receiving cisplatin/paclitaxel, cisplatin/gemcitabine, cisplatin/docetaxel or carboplatin/paclitaxel. Each doublet produced response rates of nearly 20% and a median survival of approximately 8 months.

More recently, several randomized Phase III trials have since highlighted the importance of tumor histology as an important determinant in treatment decisions. In a recent Phase III non-inferiority trial comparing cisplatin/gemcitabine versus cisplatin/pemetrexed in 1725 previously untreated patients, there was no difference in OS between the two arms (Scagliotti et al. 2008). In preplanned subgroup analysis, however, patients with adenocarcinoma histology treated with cisplatin/pemetrexed had a significant improvement in median OS compared to those treated with cisplatin/gemcitabine (12.6 versus 10.9 months). Conversely, in patients with squamous cell histology, cisplatin/gemcitabine produced superior survival compared to cisplatin/pemetrexed (median OS 10.8 versus 9.4 months). Thus, pemetrexed is now approved as part of first-line treatment in advanced, non-squamous NSCLC.

In a separate study, the addition of bevacizumab to carboplatin and paclitaxel resulted in an increase in response rate from 15% to 35% and an increase in median OS from 10 to 12 months (Sandler et al. 2006).

Given the limited survival benefit conferred by cytotoxic chemotherapy, the substantial toxicities associated with therapy (such as febrile neutropenia, myelosuppression, nausea, alopecia, nephropathy, and neuropathy) and the generally poorly tolerated regimens by elderly and poor-performance-status patients, novel therapies that deliver an improved therapeutic index are urgently needed for NSCLC.

1.1.2 Cancer Immunotherapy: Programmed Death-Ligand 1/Programmed Death-1 Inhibitors and Biomarker Selection

Immune checkpoint inhibitors, which target the programmed death-ligand 1 (PD-L1) and programmed death 1 (PD-1) axis, have emerged as a new standard of care for lung cancer and other solid tumors. Such immunotherapy agents harness the patient's own T cells to kill tumor cells and have resulted in encouraging results from Phase II/III clinical studies for recurrent 2L+ NSCLC. However, only a proportion of patients, roughly 20%–30%, have durable long-term benefit (Barlesi et al.2016; Borghaei et al. 2015; Brahmer et al. 2015; Vansteenkiste et al. 2015; Herbst et al. 2016). Additionally, subgroup analyses across these trials suggest that patients with activating EGFR mutations do not have improved outcomes on immunotherapy compared with treatment on standard chemotherapy, suggesting that these patients should receive appropriate targeted therapies before being offered immunotherapy. It remains unknown, however, if checkpoint inhibitors have less clinical efficacy compared to standard treatments in 1L patients who have EGFR activating mutations.

Pembrolizumab, a PD-1 monoclonal antibody, is now approved by the Food and Drug Administration (FDA) for first-line treatment of NSCLC in patients with PD-L1 expression

"Use of this document is governed by the terms of use on the first page of this document."

on $\geq$ 50% of tumor cells. In the Phase III label–enabling trial, PD-L1 expression was determined using tissue-based immunohistochemistry (IHC). Treatment with pembrolizumab resulted in an OS hazard ratio (HR) of 0.6 (median OS not reached for either treatment arm) and progression-free survival (PFS) HR of 0.5 (median PFS 10.3 vs. 6.0 months) in favor of pembrolizumab over platinum-based chemotherapy (Reck et al. 2016). These impressive results are changing the current treatment paradigm for first-line therapy and have encouraged upfront tissue testing for PD-L1 to identify patients with high PD-L1 tumor expression ($\geq$ 50% PD-L1, approximately 30% prevalence). These data also provide further evidence to support the use of atezolizumab in a similarly PD-L1 enriched population as in the current clinical trial.

A separate first-line Phase III trial of nivolumab, another PD-1 monoclonal antibody, failed to show a statistically meaningful difference in PFS between nivolumab and platinum-based chemotherapy for patients with PD-L1 expression on $\geq$ 5% of tumor cells (median PFS 4.2 vs 5.9; HR = 1.15; p = 0.2511; Socinski et al. 2016). Overall survival outcomes were equally disappointing (median OS 14.4 vs 13.2 months; HR = 1.02). The prevalence of patients with $\geq$ 5% PD-L1 was roughly 75%. The stark contrast in results from the pembrolizumab and nivolumab trials calls into question the reliability of using PD-L1 measured by IHC as a predictive biomarker and underscores the importance of identifying alternative methods of diagnostic selection for immunotherapy.

Emerging data in the field of cancer immunotherapy suggest that mutational load (or mutational burden) may be a better diagnostic for the selection of patients for PD-1/PD-L1 inhibitors. This hypothesis is supported by the notion that tumors with higher rates of somatic mutations, such as in NSCLC, may have increased immunogenicity and are therefore more susceptible to checkpoint inhibition (Chen et al. 2012). In a recent study conducted by Rizvi et al. (2015), investigators reported that nonsynonymous mutation burden, as determined by whole-exome sequencing of NSCLC patients treated with pembrolizumab, was associated with improved outcomes such as ORRs, durable clinical benefit, and progression-free survival (PFS).

Additional considerations in the diagnostic selection of patients include whether an appropriate quality and quantity of tissue samples can be collected to test for the increasing array of oncogenic driver mutations. Advances in diagnostic technologies are enabling newer less-invasive genomic profiling in the blood via detection of circulating tumor DNA (ctDNA) (Tsui et al. 2016). Currently, commercial tests of ctDNA are available to guide treatment for targeted therapies. The next advance in this field will be to develop a ctDNA-based diagnostic to guide treatment for immunotherapies through detection of mutational burden in the blood.

"Use of this document is governed by the terms of use on the first page of this document."

1.1.3 Diagnostic Testing for First-Line Treatment of Non–Small Cell Lung Cancer

The National Comprehensive Cancer Network (NCCN) treatment guidelines describe PD-L1 testing by IHC as part of the molecular diagnostic studies to be performed in lung cancer (NCCN Guidelines 2016). Additionally, NCCN recommends upfront testing for EGFR and ALK to determine appropriate first-line therapy in patient subsets. Of note, in patients with squamous cell carcinoma, the observed incidence of EGFR mutations is 2.7%. This frequency of EGFR mutations does not justify routine testing of all tumor specimens (NCCN Guidelines 2017). Similarly, EGFR and KRAS mutations are mutually exclusive, with overlapping mutations occurring in < 1% of patients with lung cancer (Riely et al. 2006). Therefore, patients with confirmed KRAS mutations do not require further EGFR testing for eligibility for the trial (see Section 4.1.2). See Study Rationale and Benefit–Risk Assessment section for additional details (Section 1.3).

1.2 BACKGROUND ON ATEZOLIZUMAB

Atezolizumab (Tecentriq®) is a humanized immunoglobulin G subclass 1 (IgG1) monoclonal antibody consisting of two heavy chains (448 amino acids) and two light chains (214 amino acids) and is produced in Chinese hamster ovary cells. Atezolizumab was engineered to eliminate Fc-effector function via a single amino acid substitution at position 298 on the heavy chain, which results in a non-glycosylated antibody that has minimal binding to Fc receptors and prevents Fc-effector function at expected concentrations in humans. Atezolizumab targets human PD-L1 and inhibits its interaction with its receptors, PD-1 and B7.1 (CD80, B7-1). Both of these interactions are reported to provide inhibitory signals to T cells.

Atezolizumab (Tecentriq®) is approved in the United States for the treatment of locally advanced or metastatic urothelial cancer (UC), *in combination with paclitaxel for adult patients with locally advanced metastatic triple negative breast cancer whose tumors express PD-L1, in combination with bevacizumab and chemotherapy for first- line NSCLC, first-line extensive-stage small cell lung cancer,* and metastatic NSCLC that has progressed during or following treatment with a platinum-containing regimen. Atezolizumab is also being investigated as a potential therapy against solid tumors and hematologic malignancies in humans.

Refer to the Atezolizumab Investigator's Brochure for details on nonclinical and clinical studies.

1.2.1 Summary of Nonclinical Studies

The nonclinical strategy of the atezolizumab program was to demonstrate in vitro and in vivo activity, to determine in vivo pharmacokinetic (PK) behavior, to demonstrate an acceptable safety profile, and to identify a Phase I starting dose. Comprehensive pharmacology, PK, and toxicology evaluations were thus undertaken with atezolizumab.

"Use of this document is governed by the terms of use on the first page of this document."

The safety, pharmacokinetics, and toxicokinetics of atezolizumab were investigated in mice and cynomolgus monkeys to support intravenous (IV) administration and to aid in projecting the appropriate starting dose in humans. Given the similar binding of atezolizumab for cynomolgus monkey and human PD-L1, the cynomolgus monkey was selected as the primary and relevant nonclinical model for understanding the safety, pharmacokinetics, and toxicokinetics of atezolizumab.

Overall, the nonclinical pharmacokinetics and toxicokinetics observed for atezolizumab supported entry into clinical studies, including providing adequate safety factors for the proposed Phase I starting doses. The results of the toxicology program were consistent with the anticipated pharmacologic activity of downmodulating the PD-L1/PD-1 pathway and supported entry into clinical studies in patients.

Refer to the Atezolizumab Investigator's Brochure for details on the nonclinical studies.

1.2.2 Clinical Experience with Atezolizumab

Atezolizumab clinical data are available from multiple Phase I, II, and III studies, both as monotherapy and in combination with several anti-cancer therapies (see the Atezolizumab Investigator's Brochure for study descriptions).

Single-agent safety and efficacy data in patients with lung cancer are presented below from the following studies:

- Study PCD4989g: A Phase Ia, multicenter, first-in-human, open-label, dose-escalation study evaluating the safety, tolerability, immunogenicity, pharmacokinetics, exploratory pharmacodynamics, and preliminary evidence of biological activity of atezolizumab administered as a single agent by IV infusion given every 21 days (q21d) to patients with locally advanced or metastatic solid malignancies or hematologic malignancies.
- Study GO28753 (POPLAR): A randomized, Phase II, open-label study assessing the clinical benefit of atezolizumab as a single agent versus docetaxel in PD-L1–unselected patients with locally advanced or metastatic NSCLC that has progressed during or following treatment with a platinum-containing regimen.
- Study GO28754 (BIRCH): A Phase II, multicenter, single-arm study assessing the clinical benefit of atezolizumab as a single agent in PD-L1–selected patients with locally advanced or metastatic NSCLC.
- Study GO28915 (OAK): A randomized, Phase III, open-label study assessing the clinical benefit of atezolizumab as a single agent versus docetaxel in PD-L1-unselected patients with locally advanced or metastatic NSCLC that has progressed during or following treatment with a platinum-containing regimen.

"Use of this document is governed by the terms of use on the first page of this document."

1.2.2.1 Clinical Safety

Single-Agent Clinical Safety in Patients with Non–Small Cell Lung Cancer in Study PCD4989g

Study PCD4989g is a Phase Ia dose escalation and expansion study in which atezolizumab is being used as a single agent in patients with locally advanced or metastatic solid tumors or hematologic malignancies. It provides significant data (with 629 safety-evaluable patients across all cancer types as of the data cutoff date of 15 December 2015) for the safety profile of atezolizumab as monotherapy.

Currently, no maximum tolerated dose (MTD), no dose-limiting toxicities (DLTs), and no clear dose-related trends in the incidence of adverse events have been determined.

The safety profile of atezolizumab as a single agent is observed to be consistent across different indications, including small cell lung cancer, NSCLC, UC, renal cell carcinoma (RCC), melanoma, gastric cancer, colorectal cancer, head and neck cancer, breast cancer, and sarcoma.

Of the 629 patients across all cancer types in Study PCD4989g, 619 patients (98.4%) experienced at least one adverse event, including 444 patients (70.6%) who experienced one treatment-related adverse event. Commonly reported events (reported in $\geq$10% of all patients) included fatigue, nausea, decreased appetite, diarrhea, constipation, dyspnea, pyrexia, and cough (see Table 1).

A total of 89 safety-evaluable patients with NSCLC received atezolizumab in Study PCD4989g. A total of 88 patients (98.9%) experienced at least one adverse event, including 67 patients (75.3%) with treatment-related adverse events, 35 (39.3%) patients with Grade 3–4 adverse events, 36 patients (40.4%) with serious adverse events, 5 patients (5.6%) who discontinued study drug due to an adverse event, and 1 death (1.1%).

The safety profile of the NSCLC cohort was consistent with the overall safety profile of all safety-evaluable patients in Study PCD4989g, as well as with the safety-evaluable patients with NSCLC who received atezolizumab monotherapy in other studies.

"Use of this document is governed by the terms of use on the first page of this document."

Table 1 Adverse Events Reported in ≥10% of Patients in Study PCD4989g

Preferred Term	All Grades n (%)
Any adverse event (≥10% incidence)	592 (94.1%)
Fatigue	248 (39.4%)
Nausea	175 (27.8%)
Decreased appetite	166 (26.4%)
Diarrhea	141 (22.4%)
Constipation	136 (21.6%)
Dyspnea	135 (21.5%)
Pyrexia	134 (21.3%)
Cough	127 (20.2%)
Vomiting	124 (19.7%)
Anemia	121 (19.2%)
Back Pain	111 (17.6%)
Headache	104 (16.5%)
Asthenia	101 (16.1%)
Arthralgia	95 (15.1%)
Pruritus	89 (14.1%)
Rash	82 (13.0%)
Abdominal pain	77 (12.2%)
Edema peripheral	72 (11.4%)
Urinary tract infection	67 (10.7%)
Insomnia	66 (10.5%)
Dizziness	63 (10.0%)

Single-Agent Clinical Safety in Patients with Non–Small Cell Lung Cancer in Study GO28753 (POPLAR)

As of the 1 December 2015 data cutoff date, 142 patients with NSCLC were treated with atezolizumab as a fixed dose of 1200 mg IV every 3 weeks and 135 patients were treated with docetaxel 75 mg/m^2 IV q21d in Study GO28753. The frequency of patients with any reported adverse event regardless of attribution was 96% in both arms. Fewer patients in the atezolizumab arm (41%) experienced Grade 3–4 adverse events compared with the docetaxel arm (53%). For Grade 3–4 adverse events that were assessed as treatment-related, the difference was greater between the two arms (12% vs. 39%, respectively). The most common atezolizumab-related Grade 3 adverse events were pneumonia (2%) and increased aspartate aminotransferase (2%). No

"Use of this document is governed by the terms of use on the first page of this document."

atezolizumab-related Grade 4 events have been reported. Treatment-related adverse events reported in at least 10% of patients in either treatment arm are listed in Table 2.

Table 2 Treatment-Related Adverse Events Reported in at Least 10% of Patients in Either Treatment Arm in Study GO28753 (POPLAR)

MedDRA Preferred Term	Atezolizumab (n = 142) No. (%)	Docetaxel (n = 135) No. (%)
Fatigue	55 (38.7%)	54 (40.0 %)
Decreased appetite	49 (34.5%)	28 (20.7%)
Nausea	32 (22.5%)	45 (33.3%)
Cough	40 (28.2%)	33 (24.4%)
Dyspnoea	39 (27.5%)	27 (20.0%)
Constipation	31 (21.8%)	32 (23.7%)
Diarrhoea	25 (17.6%)	38 (28.1%)
Alopecia	3 (2.1%)	52 (38.5%)
Anaemia	25 (17.6%)	27 (20.0%)
Pyrexia	24 (16.9%)	16 (11.9%)
Vomiting	20 (14.1%)	18 (13.3%)
Asthenia	15 (10.6%)	22 (16.3%)
Arthralgia	22 (15.5%)	12 (8.9%)
Insomnia	22 (15.5%)	11 (8.1%)
Rash	16 (11.3%)	16 (11.9%)
Back pain	16 (11.3%)	11 (8.1%)
Myalgia	9 (6.3%)	18 (13.3%)
Musculoskeletal pain	19 (13.4%)	7 (5.2%)
Weight decreased	16 (11.3%)	9 (6.7%)
Haemoptysis	15 (10.6%)	8 (5.9%)
Pneumonia	17 (12.0%)	4 (3.0%)
Neuropathy peripheral	3 (2.1%)	16 (11.9%)
Neutropenia	2(1.4%)	17 (12.6%)

Single-Agent Clinical Safety in Patients with Non–Small Cell Lung Cancer in Study GO28754 (BIRCH)

As of the 1 December 2015 data cutoff date, 659 patients with NSCLC have been treated with atezolizumab as a fixed dose of 1200 mg IV q21d. In Study GO28754, 93.8% of patients experienced at least one adverse event, 65% of patients experienced one treatment-related adverse event, and 12% of patients experienced a Grade ≥ 3 treatment-related adverse event.

"Use of this document is governed by the terms of use on the first page of this document."

Single-Agent Clinical Safety in Patients with Non-Small Cell Lung Cancer in Study GO28915 (OAK)

As of the July 2016 data cutoff date, 609 patients with NSCLC were treated with atezolizumab as a fixed dose of 1200 mg IV q3w and 578 patients were treated with docetaxel 75 mg/m^2 IV q3w in Study GO28915. Fewer patients in the atezolizumab arm (37%) experienced Grade 3–4 adverse events compared with the docetaxel arm (54%). For Grade 3–4 adverse events that were assessed as treatment-related, the difference was greater between the two arms (15% vs. 43%, respectively). Rates of immune-mediated adverse events were low in patients treated with atezolizumab—pneumonitis (1%), hepatitis (0.3%), and colitis (0.3%). Adverse event-related discontinuation rates were 8% with atezolizumab arm vs. 19% for docetaxel arm.

For additional information, refer to the Atezolizumab Investigator's Brochure.

1.2.2.2 Clinical Activity

Anti-tumor activity, including Response Evaluation Criteria in Solid Tumors (RECIST) version 1.1–based responses, have been observed in patients with different tumor types (including NSCLC, RCC, melanoma, gastric cancer, UC, colorectal cancer, head and neck cancer, breast cancer, and sarcoma) treated with atezolizumab in Study PCD4989g.

Refer to the Atezolizumab Investigator's Brochure for details on clinical activity in all patients treated to date, regardless of tumor type.

Single-agent data from Studies PCD4989g and GO28754 (BIRCH) and data from the randomized Study GO28753 (POPLAR) in patients with advanced NSCLC are summarized below.

Single-Agent Clinical Activity in Patients with Non–Small Cell Lung Cancer in Study PCD4989g

As of the 2 December 2014 cutoff date, 88 patients with NSCLC in Study PCD4989g who received their first dose of atezolizumab by 21 October 2013 were evaluable for efficacy. The median age was 60.5 years; the group represented a heavily pretreated patient population. RECIST responses (confirmed) were observed in 20 of 88 (22.7%) patients, inclusive of squamous and non-squamous histologies and across all treatment cohorts (treatment dose levels: 1–20 mg/kg). A total of 8 of the 20 responding patients have continued to respond at the time of the clinical data cutoff.

Single-Agent Clinical Activity in Patients with Non–Small Cell Lung Cancer in Study GO28753 (POPLAR)

In Study GO28753 (POPLAR), demographic characteristics were comparable between the atezolizumab and docetaxel treatment arms in the intent-to-treat (ITT) population. The median age was 62 years in both treatment arms, and the majority of patients had one prior therapy (65% for atezolizumab and 67% for docetaxel), non-squamous

"Use of this document is governed by the terms of use on the first page of this document."

histology (66% for atezolizumab and 66% for docetaxel), and an Eastern Cooperative Oncology Group (ECOG) performance status of 1 (68% for atezolizumab and 68% for docetaxel). More female patients were enrolled in the docetaxel arm (35% vs. 47%).

At the time of primary analysis on 8 May 2015, there were 287 efficacy-evaluable patients (ITT population), 143 in the docetaxel arm and 144 in the atezolizumab arm. Median OS was 12.6 months for atezolizumab compared with 9.7 months for docetaxel (hazard ratio [HR] 0.73; 95% CI: 0.53, 0.99). Increasing improvement in OS was associated with increasing PD-L1 expression. Survival was similar to docetaxel in patients lacking PD-L1 expression and improved with atezolizumab in both responders and non-responders. PFS and ORR were similar between the two treatment arms in the ITT population (Fehrenbacher et al. 2016).

Single-Agent Clinical Activity in Patients with Non–Small Cell Lung Cancer in Study GO28754 (BIRCH)

In the BIRCH study, 659 PD-L1–selected patients with advanced NSCLC were treated with atezolizumab; 139 patients were naive to prior chemotherapy (Cohort 1) and 520 patients had received at least one prior platinum-based chemotherapy regimen (Cohorts 2 and 3). ORR was 19% in Cohort 1 and 17% in the other two cohorts. The majority of responses were ongoing and duration of response and OS were not yet reached (Besse et al. 2015).

Single-Agent Clinical Activity in Patients with Non-Small Cell Lung Cancer in Study GO28915 (OAK)

In the OAK study, demographic characteristics were comparable between the atezolizumab and docetaxel treatment arms in the ITT population. The majority of patients had one prior therapy (75% in both arms), non-squamous histology (74% in both arms), history of tobacco use (80% for atezolizumab and 83% for docetaxel), and ECOG Performance Status of 1 (64% for atezolizumab and 62% for docetaxel).

At the primary analysis, there were 850 efficacy-evaluable patients (ITT population): 425 in the docetaxel arm and 425 in the atezolizumab arm. Median OS in the ITT population was 13.8 months for atezolizumab compared with 9.6 months for docetaxel (HR of 0.73; 95% CI: 0.62, 0.87, $p = 0.0003$). OS benefit was seen regardless of PD-L1 expression (HR of 0.75 in $<1\%$ PD-L1 expression population; 0.41 in $\geq 50\%$ tumor cell or $\geq 10\%$ immune cell expression population) and was consistent across subgroups, including histology (HR of 0.73 for both), in patients with asymptomatic central nervous system metastases (HR of 0.54) and never smokers (HR of 0.71). PFS and ORR were similar between the two treatment arms in the ITT population (Rittmeyer et al. 2017).

1.2.3 Clinical Pharmacokinetics and Immunogenicity

On the basis of available preliminary PK data (0.03–20 mg/kg), atezolizumab appeared to show linear pharmacokinetics at doses ≥ 1 mg/kg. For the 1-mg/kg and 20-mg/kg

"Use of this document is governed by the terms of use on the first page of this document."

dose groups, the mean apparent clearance and the mean volume of distribution at steady state had a range of 3.20 –4.44 mL/kg and 48.1–65.7mL/kg, respectively, which is consistent with the expected profile of an IgG1 antibody in humans.

Development of anti-therapeutic antibodies (ATAs) has been observed in patients in all cohorts in Study PCD4989g and was associated with changes in PK (namely, a reduction of atezolizumab C_{min} to below the PK assay lower limit of quantification), in some patients in the lower dose cohorts (0.3, 1, and 3 mg/kg). The development of detectable ATAs has not had a significant impact on pharmacokinetics for doses from 10 to 20 mg/kg. Patients dosed at the 10-, 15-, and 20-mg/kg dose levels have maintained the expected target trough levels of drug despite the detection of ATAs. To date, no clear relationship between the detection of ATAs and adverse events, infusion reactions, or efficacy has been observed.

1.2.4 Rationale for Atezolizumab Dosage

The fixed dose of 1200 mg (equivalent to an average body weight–based dose of 15 mg/kg) was selected on the basis of both nonclinical studies and available clinical data from Study PCD4989g as described below.

The target exposure for atezolizumab was projected on the basis of nonclinical tissue distribution data in tumor-bearing mice, target-receptor occupancy in the tumor, the observed atezolizumab interim pharmacokinetics in humans, and other factors. The target trough concentration (C_{trough}) was projected to be 6 mg/mL on the basis of several assumptions, including the following: 1) 95% tumor receptor saturation is needed for efficacy, and 2) the tumor interstitial concentration–to-plasma ratio is 0.30 based on tissue distribution data in tumor-bearing mice.

The atezolizumab dose is also informed by available clinical activity, safety, pharmacokinetics, and immunogenicity data. Anti-tumor activity has been observed across doses from 1 mg/kg to 20 mg/kg. The MTD of atezolizumab was not reached, and no DLTs have been observed at any dose in Study PCD4989g. Currently available PK and anti-therapeutic antibody (ATA) data suggest that the 15-mg/kg atezolizumab q21d regimen (or fixed-dose equivalent) for Phase II and Phase III studies would be sufficient to both maintain a $C_{trough} \geq 6\ \mu g/mL$ and further safeguard against both interpatient variability and the potential effect of ATAs that could lead to subtherapeutic levels of atezolizumab relative to the 10-mg/kg atezolizumab q21d regimen (or fixed-dose equivalent). From inspection of available observed C_{trough} data, moving further to the 20-mg/kg atezolizumab q21d regimen does not appear to be warranted to maintain targeted C_{trough} levels relative to the proposed 15-mg/kg atezolizumab q21d level.

Simulations (Bai et al. 2012) do not suggest any clinically meaningful differences in exposure following a fixed dose or a dose adjusted for weight. Therefore, a fixed dose of 1200 mg has been selected (equivalent to an average body weight–based dose of

"Use of this document is governed by the terms of use on the first page of this document."

15 mg/kg). Selection of a q21d dosing interval is supported by this preliminary pharmacokinetics evaluation.

See the Atezolizumab Investigator's Brochure for details regarding nonclinical and clinical pharmacology of atezolizumab.

1.3 STUDY RATIONALE AND BENEFIT–RISK ASSESSMENT

Encouraging clinical data emerging in the field of tumor immunotherapy have demonstrated that therapies focused on enhancing T cell responses against cancer can result in a significant survival benefit in patients with Stage IV cancer (Hodi et al. 2010; Kantoff et al. 2010; Chen et al. 2012).

Clinical studies in NSCLC to date have focused on biomarker enrichment strategies through selection of patients whose tumors overexpress PD-L1. In our Phase II POPLAR study of atezolizumab versus docetaxel in the refractory 2L/3L setting, PD-L1 expression on both tumor cells and immune cells was found to be an independent predictor of improved survival (Fehrenbacher et al. 2016). However, response rates were also noted in patients not expressing PD-L1 (8% in patients with no PD-L1 expression), bringing to light the question of whether PD-L1 is the optimal predictive biomarker for agents such as atezolizumab.

Continued research is underway to further characterize the molecular and genomic determinants of response for patients treated with immunotherapy. Lawrence et al. (2013) suggest that tumors such as lung cancer have a higher rate of somatic mutations and that mutations provide the potential for neoantigens that can be recognized as foreign by T cells. This hypothesis will be tested in this study by treating patients with Stage IIIB–IVB NSCLC (based on the International Association for the Study of Lung Cancer [IASLC] Lung Cancer Staging Project proposed for the eighth edition of the American Joint Committee on Cancer [AJCC] NSCLC staging system [Goldstraw et al. 2015]; see Appendix 3) with atezolizumab monotherapy and testing blood samples collected at baseline and at progression to determine relative changes in tumor mutational burden in blood (bTMB) as a predictive biomarker for efficacy.

Because the response rate of single-agent atezolizumab in first-line treatment is unknown, we plan an interim futility analysis when 50% of enrolled patients have had at least 6 months of follow-up. At this interim analysis, the study will be stopped if ORR is likely to be 5% or less compared with an ORR of 15% or more. This reference endpoint is derived from preliminary evidence with nivolumab as first-line monotherapy in NSCLC in which ORR ranged from 15% to 26% depending on histology (Gettinger et al. 2015). Additionally, historical response rates for standard-of-care platinum-doublet chemotherapy are roughly 15% to 20% (Schiller et al. 2002).

Study ML39237 will enroll patients who are naive to immunotherapy and for whom atezolizumab can represent a reasonable benefit–risk option. Patients whose tumors

"Use of this document is governed by the terms of use on the first page of this document."

are known to harbor sensitizing EGFR mutations or ALK rearrangements should be treated with appropriate targeted agents such as an EGFR tyrosine kinase or ALK inhibitor, respectively (see specific exclusion criteria in Section 4.1.2), and should not be enrolled in the study. Patients are eligible for study regardless of PD-L1 status, even if the status is unknown (see specific inclusion criteria in Section 4.1.1).

In order to account for the possibility of pseudoprogression/tumor-immune infiltration (i.e., a radiographic increase in tumor volume caused by the influx of immune cells) (Hales et al. 2010) and the potential for delayed anti-tumor activity, this study will allow patients treated with atezolizumab to receive treatment beyond the initial apparent radiographic progression (see Section 3.3.3) to evaluate clinical benefit. Although the risk of pseudoprogression in NSCLC is deemed to be relatively low compared with melanoma (Chiou and Burotto 2015), it is not yet possible to reliably differentiate pseudoprogression/tumor-immune infiltration from true tumor progression by using standard radiographic assessments. Therefore, the risk does exist that some patients who are not responding to treatment but are continuing to receive atezolizumab may experience further progression of NSCLC and may have a delay in treatment with subsequent therapies for which they are eligible. Investigators should make every effort to fully inform patients of this risk. Investigators should make a careful assessment of the potential benefit of continuing treatment with atezolizumab, taking into consideration radiographic data and the clinical status of the patient. If, after an integrated assessment of radiographic data and clinical status, the decision is made to continue treatment with atezolizumab following apparent radiographic progression, patients for whom alternative approved anti-cancer therapies exist must provide written consent at that time to acknowledge deferring these treatment options in favor of continuing study drug.

Atezolizumab has been generally well tolerated (see Section 1.2.2.2). Adverse events with potentially immune-mediated causes consistent with an immunotherapeutic agent, including rash, hypothyroidism, hepatitis/transaminitis, colitis, and myasthenia gravis, have been observed in Study PCD4989g. To date, these events have been manageable with treatment.

In summary, treatment with atezolizumab as first-line therapy offers the potential for clinical benefit in patients with NSCLC. Because most atezolizumab-related toxicities observed to date have been mild and transient in nature and are different from the adverse events observed from chemotherapy, the benefit–risk to patients is favorable for atezolizumab, especially when compared with the benefit–risk for conventional chemotherapy. Because of the phenomenon of pseudoprogression, patients and investigators can elect to continue therapy beyond radiographic progression. Patients should be fully informed of the risk of continuing study drug in spite of apparent radiographic progression, and investigators should make a careful assessment of the potential benefit of doing so, taking into consideration radiographic data, biopsy results (if available), and the clinical status of the patient.

"Use of this document is governed by the terms of use on the first page of this document."

2. OBJECTIVES AND ENDPOINTS

The aim of this study is to evaluate the clinical efficacy of atezolizumab as a single-agent, first-line therapy in patients with immunotherapy–naive, Stage IIIB–IVB NSCLC. Diagnostic selection of patients will not be a criterion for enrollment.

The primary biomarker objective is to measure tumor mutational burden from blood (bTMB) and to evaluate whether it can predict for improved clinical outcomes with atezolizumab. Clinical outcome, as measured by PFS, will be correlated to bTMB retrospectively to define the threshold for positivity. Specific objectives and corresponding endpoints for the study are outlined in Table 3.

Table 3 Objectives and Corresponding Endpoints

Objectives	Corresponding Endpoints
**Primary Efficacy Objective:**	
• To evaluate the clinical efficacy of atezolizumab	• INV-assessed ORR by RECIST v1.1
**Secondary Efficacy Objective:**	
• To evaluate the efficacy of atezolizumab	• *INV*-assessed ORR by RECIST v1.1<br>• INV-assessed PFS by RECIST v1.1<br>• INV-assessed duration of response by RECIST v1.1<br>• Overall survival
**Safety Objective:**	
• To evaluate the safety and tolerability of atezolizumab	• Incidence of adverse events, with severity determined through use of NCI CTCAE v4.0
**Primary Biomarker Objective:**	
• To evaluate whether "positive vs. negative bTMB" defined at various bTMB quantiles can predict for improved PFS with atezolizumab	• Relationship between INV-assessed PFS and various bTMB quantiles
**Secondary Biomarker Objectives**	
• To evaluate the correlation between clinical outcomes including but not limited to PFS, PFS rate at 6, 9, and 12 months, ORR, and various definitions of positive bTMB	• Relationship between efficacy endpoints including INV-assessed PFS rate at 6, 9, and 12 months, ORR and various bTMB quantiles and efficacy
**Exploratory Biomarker Objectives in Blood:**	
• To evaluate whether higher expression of an immune-related gene signature in blood PBMCs predicts for improved PFS with atezolizumab	• Relationship between PFS and expression of an immune gene signature in PBMCs
• To assess whether bTMB is altered as a result of treatment with immunotherapy	• Relationship between bTMB and treatment with immunotherapy
• To assess the status of additional circulating	• Relationship between circulating biomarkers

"Use of this document is governed by the terms of use on the first page of this document."

Objectives	Corresponding Endpoints
biomarkers related to immunotherapy and NSCLC and outcomes with atezolizumab	related to immunotherapy and NSCLC and efficacy
**Exploratory Biomarker Objectives in Tissue:**	
• To perform retrospective tTMB analysis and NGS-based mutation testing on tissue biopsies for patients providing specific consent and viable samples	• Relationship between somatic mutations and tTMB on efficacy. All mutations will be identified through NGS performed on DNA extracted from tumor tissue.

bTMB = blood tumor mutational burden; INV = investigator; NGS = next-generation sequencing; NCI CTCAE = National Cancer Institute Common Terminology Criteria for Adverse Events; NSCLC = non–small cell lung cancer; ORR = objective response rate; PBMC = peripheral blood mononuclear cell; PFS = progression-free survival; RECIST = Response Evaluation Criteria in Solid Tumors; tTMB = tissue tumor mutational burden.

3. STUDY DESIGN

3.1 DESCRIPTION OF THE STUDY

This is a Phase II, open-label, prospective, multicenter study designed to evaluate the efficacy and safety of single-agent atezolizumab as a first-line therapy in patients with locally advanced or metastatic NSCLC. In addition, the primary biomarker objective is to measure bTMB and evaluate whether it can predict for improved clinical outcome with atezolizumab.

There will be approximately 150 patients with Stage IIIB–IVB, locally advanced or metastatic, EGFR/ALK–negative NSCLC who are immunotherapy-naive enrolled at approximately 25–30 study sites in the United States.

Figure 1 illustrates the study design.

"Use of this document is governed by the terms of use on the first page of this document."

Figure 1 Study Schema

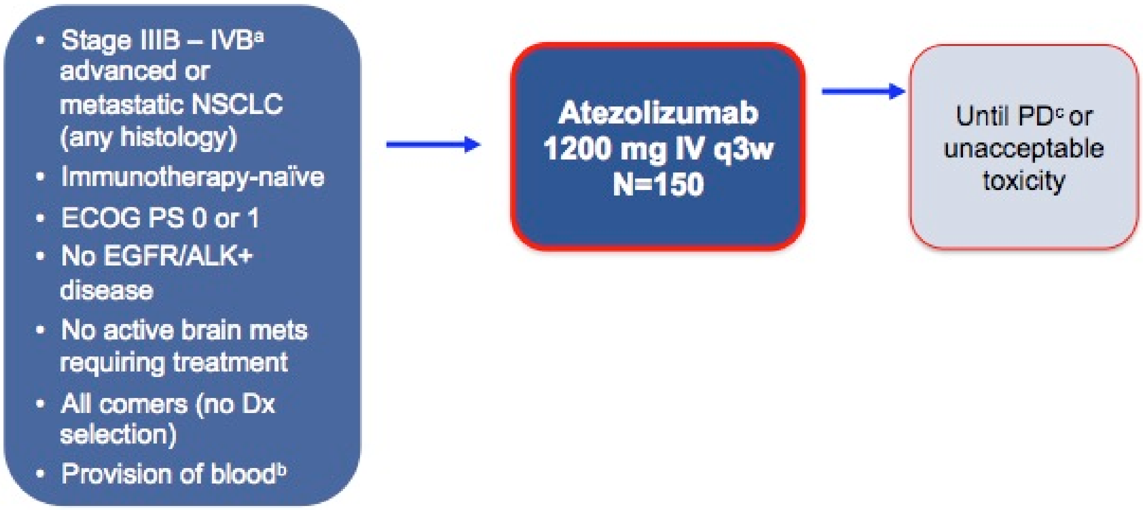

AJCC = American Joint Committee on Cancer; Dx = diagnosis; ECOG = Eastern Cooperative Oncology Group; EGFR/ALK = epidermal growth factor receptor/anaplastic lymphoma kinase; IASLC = International Association for the Study of Lung Cancer; IV = intravenous; mets = metastases; NSCLC = non–small cell lung cancer; PD = progressive disease; PS = performance status; q3w = once every three weeks; RECIST = Response Evaluation Criteria in Solid Tumors.

[a] Staging criteria based on the IASLC Lung Cancer Staging Project proposed for the eighth edition of the AJCC NSCLC staging system (see Appendix 3). Patients with histologically or cytologically confirmed Stage IIIB-IV NSCLC based on the seventh edition of the AJCC NSCLC staging system are also eligible for study entry.

[b] Blood samples will be taken at baseline and at progression (all samples will be tested retrospectively).

[c] Treatment beyond RECIST v1.1 progression may be permitted if the patient continues to derive clinical benefit as determined by the investigator.

Atezolizumab (fixed dose of 1200 mg) will be administered intravenously on Day 1 of each 21-day cycle.

Patients who meet the Response Evaluation Criteria in Solid Tumors (RECIST) v1.1 criteria for progressive disease (PD) at any timepoint during treatment will be permitted to continue atezolizumab treatment if there is evidence of clinical benefit, defined as meeting all of the following criteria:

- Evidence of clinical benefit as assessed by the investigator
- Absence of symptoms and signs (including worsening of laboratory values [e.g., new or worsening hypercalcemia]) indicating unequivocal progression of disease
- No decline in ECOG performance status that can be attributed to disease progression
- Absence of tumor progression at critical anatomical sites (e.g., leptomeningeal disease) that cannot be managed by protocol-allowed medical interventions

"Use of this document is governed by the terms of use on the first page of this document."

Patients for whom approved therapies exist must provide written consent to acknowledge deferring these treatment options in favor of continuing study drug at the time of initial progression.

Screening tests and evaluations will be performed within 28 days prior to treatment initiation.

Blood samples will be collected at screening, baseline, during therapy, and at first evidence of radiographic disease progression or loss of clinical benefit. Data from the latter blood draws will be used to explore whether the radiographic findings are consistent with changes in bTMB as measured by the blood-based diagnostic assays and to explore possible mutational mechanisms of resistance. Additional blood samples may be taken at each scan as defined in Appendix 1.

Tissue biopsies (tissue blocks preferred) may be submitted at any time during the study, with biopsies from diagnosis (archival or fresh) and at progression preferred. For patients who provide tissue samples, next generation sequencing (NGS) may be performed by Foundation Medicine. If performed by Foundation Medicine, the investigator can obtain results from these analyses in the form of an NGS report, which is available upon request directly from Foundation Medicine. The investigator may share and discuss the results with the patient, unless the patient chooses otherwise. The Foundation Medicine NGS assay, FoundationOne®, has not been cleared or approved by health authorities. The NGS report is generated for research purposes and is not provided for the purpose of guiding future treatment decisions (see Section 4.5.7). Results may not be available for samples that do not meet testing criteria. Patients who are unable to undergo biopsy sample collection but otherwise meet the eligibility criteria listed in Section 4.1 may still be enrolled to receive atezolizumab.

All patients will undergo tumor assessment at baseline and every 6 weeks (± 7 days) thereafter regardless of dose delays for the first 12 months following Cycle 1, Day 1. After 12 months, tumor assessment will be required every 9 weeks (± 7 days); tumor assessments will continue until disease progression per RECIST v1.1 or loss of clinical benefit, consent withdrawal, study discontinuation, study completion (i.e., end of study as defined in Section 3.2) or death, whichever occurs first. Patients who discontinue treatment for reasons other than disease progression (e.g., toxicity) will continue scheduled tumor assessments until disease progression, withdrawal of consent, study discontinuation, study completion (i.e., end of study), or death, whichever occurs first. In the absence of disease progression, tumor assessments should continue regardless of whether patients start a new anti-cancer therapy, unless consent is withdrawn.

All patients will be followed for survival and anti-cancer therapy while in the study and will be given the option of participating in a long-term follow-up*, which will include collection of long-term survival data beyond the end of the study.* The optional long-term follow-up will begin following the end of the study (as defined in Section 3.2).

"Use of this document is governed by the terms of use on the first page of this document."

Patients must provide additional consent to participate in this optional long-term follow-up. *Patients on study drug will continue therapy until they have received a total of 2 years of atezolizumab treatment (32 cycles). Thereafter, patients will continue overall survival follow-up every 3 months for the first year, then every 6 months thereafter, for up to 5 years from enrollment of the first patient into the study, until the last patient dies, withdraws consent, or is lost to follow-up, whichever occurs first.*

Safety will be assessed on the basis of vital sign measurements, ECOG performance status scores, physical examination findings, clinical laboratory test results, and the frequency, severity, and relationship to atezolizumab of adverse events.

When a patient discontinues study drug, regardless of the reason for discontinuation, the patient will be asked to return to the clinic within 30 days after the last dose of study drug for a treatment discontinuation visit. Patients who discontinue will not be replaced.

A schedule of activities is provided in Appendix 1.

3.2 END OF STUDY AND LENGTH OF STUDY

The end of this study is defined as the date after which all enrolled patients who remain alive have been followed for at least 18 months from study-drug initiation. Thereafter, patients *who discontinue study drug* will be followed for *optional* long-term survival as described in Section 4.5.8.

Patients who remain on study drug will continue therapy until they have received a total of 2 years of atezolizumab treatment (32 cycles), and will be followed for safety. Survival data will be collected during the optional long-term follow up period every 3-months for the first year, then every 6 months thereafter, for up to 5 years from enrollment of the first patient into the study, until the last patient dies, withdraws consent, or is lost to follow-up, whichever occurs first

The total length of the study, from screening of the first patient to the end of the study, is expected to be approximately 3 years. *In addition, the Sponsor may decide to terminate the study at any time.*

3.3 RATIONALE FOR STUDY DESIGN

3.3.1 Rationale for Atezolizumab Dose and Schedule

The fixed dose of 1200 mg (equivalent to an average body weight–based dose of 15 mg/kg) was selected on the basis of both nonclinical studies and available clinical data from Study PCD4989g as described below. This is the same dose and schedule being evaluated in the Phase II and Phase III program in NSCLC, urothelial carcinoma, and other solid tumors.

The target exposure for atezolizumab was projected on the basis of nonclinical tissue distribution data in tumor-bearing mice, target-receptor occupancy in the tumor, the

"Use of this document is governed by the terms of use on the first page of this document."

observed atezolizumab interim pharmacokinetics in humans, and other factors. The target C_{trough} was projected to be 6 μg/mL on the basis of several assumptions, including the following: 1) 95% tumor receptor saturation is needed for efficacy, and 2) the tumor interstitial concentration–to-plasma ratio is 0.30 based on tissue distribution data in tumor-bearing mice.

The atezolizumab dose is also informed by available clinical activity, safety, PK, and immunogenicity data. Anti-tumor activity has been observed across doses from 1 mg/kg to 20 mg/kg. The MTD of atezolizumab was not reached, and no DLTs have been observed at any dose in Study PCD4989g. Available preliminary PK data (0.03–20 mg/kg) from Study PCD4989g suggest that for doses $\geq$ 1 mg/kg, overall atezolizumab exhibits pharmacokinetics that are both linear and consistent with typical IgG1 antibodies. Detectable ATAs were observed in patients at all dose levels but were associated with changes in pharmacokinetics for some patients in only the lower dose cohorts (0.3, 1, and 3 mg/kg). It is unclear from currently available data in these lower dose cohorts whether the administration of higher doses to patients with both detectable ATAs and reduced exposure would necessarily restore exposure to expected levels. No clear relationship between the development of measurable ATAs and safety or efficacy has been observed. Available data suggest that the development of detectable ATAs does not appear to have a significant impact on the pharmacokinetics for doses from 10 to 20 mg/kg in most patients. Correspondingly, patients dosed at the 10-, 15-, and 20-mg/kg dose levels have maintained target trough levels of drug despite the detection of ATAs. Currently available PK and ATA data suggest that the 15-mg/kg atezolizumab q21d regimen (or fixed-dose equivalent) for Phase II and Phase III studies would be sufficient to both maintain a $C_{trough} \geq 6$ μg/mL and further safeguard against both interpatient variability and the potential effect of ATAs that could lead to subtherapeutic levels of atezolizumab relative to the 10-mg/kg atezolizumab q21d regimen (or fixed-dose equivalent). From inspection of available observed C_{trough} data, moving further to the 20-mg/kg atezolizumab q21d regimen does not appear to be warranted to maintain targeted C_{trough} levels relative to the proposed 15-mg/kg atezolizumab q21d level.

Simulations do not suggest any clinically meaningful differences in exposure following a fixed dose or a dose adjusted for weight. On the basis of this analysis, a fixed dose of 1200 mg has been selected (equivalent to an average body weight–based dose of 15 mg/kg).

Selection of a q21d dosing interval is supported by this preliminary PK evaluation.

3.3.2 Rationale for Patient Population

Despite recent improvements in treatment options, the prognosis for patients with advanced NSCLC remains dismal, with a median OS in first-line therapy of approximately 12.5 months (Sandler et al. 2006). Platinum-based doublet chemotherapy is still the current standard of care, yet response rates have not improved beyond 20% (Schiller et al. 2002). These approved chemotherapies are associated with

"Use of this document is governed by the terms of use on the first page of this document."

significant toxicities (e.g., neuropathy, febrile neutropenia, myelosuppression, and alopecia) that negatively affect quality of life. Therefore, there is a continuing need for more efficacious, better-tolerated treatments.

Inhibition of PD-L1/PD-1 signaling has been shown to produce durable responses in a proportion of patients in several solid tumor types, including NSCLC (Topalian et al. 2012). However, the majority of first-line clinical data in NSCLC are from biomarker-selected populations, or limited to patients who have been treated in refractory second-line disease and beyond. Early data from nivolumab, a PD-1 inhibitor, have been reported in first-line NSCLC with a median OS of 22.6 months (Gettinger et al. 2015) and provide a strong rationale for evaluating atezolizumab as a first-line therapy in patients with Stage IIIB–IVB NSCLC.

3.3.3 Rationale for Allowing Patients to Continue Atezolizumab Treatment until Loss of Clinical Benefit

Conventional response criteria may not adequately assess the activity of immunotherapeutic agents because PD (by initial radiographic evaluation) does not necessarily reflect therapeutic failure (see Section 1.3). Because of the potential for pseudoprogression/tumor immune infiltration, this study will allow patients to continue to receive study drug after apparent radiographic progression, provided the benefit–risk ratio is judged to be favorable by the investigator in consultation with the patient. Therapy may continue beyond radiographic PD as determined by the investigator until they deem there is further loss of clinical benefit for the patient. Loss of clinical benefit is defined as unacceptable toxicity or symptomatic deterioration attributed to disease progression as determined by the investigator after an integrated assessment of radiographic data and clinical status.

3.3.4 Rationale for Collection of Blood Specimens

The primary biomarker objective of this study is to measure bTMB in to evaluate predictive cutoffs for a novel blood-based diagnostic assay. This may help predict which patients may benefit most from atezolizumab and may help future development of tissue-free diagnostic options for patients who have inadequate quality or insufficient quantity of tissue.

Blood samples will be collected at screening, baseline, during therapy, and at first evidence of radiographic disease progression or loss of clinical benefit in order to better understand the changes in bTMB and immune signature profiles and potential mechanisms of resistance upon progression. Blood samples will be collected at screening, on Day 1 of Cycles 1, 2, and 4 (mandatory), and on Day 1 of Cycle 3 (optional), to evaluate additional exploratory biomarker changes that may be associated with the therapeutic effects of atezolizumab or the pathogenesis of NSCLC (see Appendix 2). Methods for exploratory analysis include, but are not limited to, NGS, PCR, and proteomics-based approaches.

"Use of this document is governed by the terms of use on the first page of this document."

3.3.5 Rationale for Collection of Archival and/or Fresh Tumor Specimens

In this study, archival and/or fresh tumor specimens from each patient will not be mandated but will be strongly encouraged. Tissue biopsies (tissue blocks preferred) may be submitted at any time during the study, with biopsies from diagnosis (archival or fresh) and at progression preferred. For patients who provide tissue samples, NGS may be performed by Foundation Medicine. If performed by Foundation Medicine, the investigator can obtain results from these analyses in the form of an NGS report, which is available upon request directly from Foundation Medicine. The investigator may share and discuss the results with the patient, unless the patient chooses otherwise. The Foundation Medicine NGS assay, FoundationOne®, has not been cleared or approved by health authorities. The NGS report is generated for research purposes and is not provided for the purpose of guiding future treatment decisions (see Section 4.5.7). Results may not be available for samples that do not meet testing criteria.

To assess mutational load in tissue (tissue tumor mutational burden [tTMB]), a mutational load algorithm will be applied to NGS data. Analysis will be retrospective.

In addition to the assessment of tTMB, other exploratory markers, such as those with potential predictive and prognostic value related to the clinical benefit of atezolizumab, tumor immunobiology, mechanisms of resistance, or tumor type, may also be analyzed. DNA and/or RNA extraction may be performed to enable NGS and PCR to identify somatic mutations to increase understanding of disease pathobiology.

3.3.6 Rationale for Biomarker Assessments

Despite the promising clinical efficacy of PD-L1/PD-L1 inhibitors in NSCLC, PD-L1 expression as determined by immunohistochemistry does not appear to identify all of the patients who derive clinical benefit from these drugs. Emerging evidence suggests that patients with a high burden of somatic mutations derive significant clinical benefit from anti–PD-1/PD-L1 therapy; thus, further development of mutation load as a predictive biomarker for atezolizumab is warranted.

Blood samples (mandatory) will be collected at baseline, during therapy, and at first evidence of radiographic disease progression or loss of clinical benefit. Tissue biopsies (tissue blocks preferred) may be submitted at any time during the study, with biopsies from diagnosis (archival or fresh) and at progression preferred. DNA and RNA extraction to enable analysis via NGS, expression, and/or PCR to identify somatic mutations that are predictive of response to study drug can increase the knowledge and understanding of disease biology. NGS techniques such as targeted sequencing may offer a unique opportunity to identify biomarkers of response. NGS may be performed by a clinical cancer genomic profiling laboratory (e.g., Foundation Medicine).

Genomics is increasingly informing researchers' understanding of disease pathobiology. Targeted NGS provides a multiplex characterization of the genome and, along with

"Use of this document is governed by the terms of use on the first page of this document."

clinical data collected in this study, may increase the opportunity for developing new therapeutic approaches. Data will be analyzed in the context of this study but will also be explored in aggregate with data from other studies. The availability of a larger dataset will assist in identification of important pathways, guiding the development of new targeted agents.

4. MATERIALS AND METHODS

4.1 PATIENTS

Approximately 150 patients with Stage IIIB–IVB, locally advanced or metastatic, EGFR/ALK–negative NSCLC who are immunotherapy-naive will be enrolled.

4.1.1 Inclusion Criteria

Patients must meet all of the following criteria to be eligible for study entry:

- Signed Informed Consent Form
- Age ≥ 18 years
- ECOG performance status of 0 or 1
- Histologically or cytologically confirmed Stage IIIB–IVB NSCLC (based on the IASLC Lung Cancer Staging Project proposed for the eighth edition of the AJCC NSCLC staging see Appendix 3)
  - Patients with histologically or cytologically confirmed Stage IIIB-IV NSCLC based on the seventh edition of the AJCC NSCLC staging system are also eligible for study entry.
- For patients who have received prior neo-adjuvant/adjuvant chemotherapy or chemoradiotherapy with curative intent for non-metastatic disease: a treatment-free interval of at least 6 months prior to enrollment.
- Patients with any PD-L1 test result by IHC are eligible for the study.
- Patients without a PD-L1 test result are eligible for the study.
- Measurable disease, as defined by RECIST v1.1 (based on the assessments detailed in Section 4.5.5.
  - Previously irradiated lesions can only be considered measurable disease if disease progression has been unequivocally documented at that site since radiation and the previously irradiated lesion is not the only site of measureable disease
- Adequate hematologic and end-organ function, defined by the following laboratory test results obtained within 14 days prior to the first dose of study drug:
  - ANC ≥ 1500 cells/μL without granulocyte colony-stimulating factor support
  - Lymphocyte count ≥500 cells/μL
  - Platelet count ≥100,000 cells/μL without transfusion
  - Hemoglobin ≥9.0 g/dL

"Use of this document is governed by the terms of use on the first page of this document."

Patients may be transfused to meet this criterion.

- International normalized ratio (INR) or activated partial thromboplastin time (aPTT) $\leq$1.5 $\times$ the upper limit of normal (ULN)

  This applies only to patients who are not receiving therapeutic anticoagulation; patients receiving therapeutic anticoagulation must have an INR or aPTT within therapeutic limits for at least 1 week prior to enrollment.

- AST, ALT, and alkaline phosphatase $\leq$2.5 $\times$ ULN with the following exceptions:

  Patients with documented liver metastases: AST and/or ALT $\leq$5 $\times$ ULN

  Patients with documented liver or bone metastases: alkaline phosphatase $\leq$5 $\times$ ULN

- Serum bilirubin $\leq$1.5 $\times$ ULN

  Patients with known Gilbert disease who have a serum bilirubin level $\leq$3 $\times$ ULN may be enrolled.

- Calculated creatinine clearance $\geq$ 30 mL/min

* For women of childbearing potential: agreement to remain abstinent (refrain from heterosexual intercourse) or use a contraceptive method with a failure rate of <1% per year during the treatment period and for at least 5 months after the last dose of atezolizumab.
  - A woman is considered to be of childbearing potential if she is postmenarcheal, has not reached a postmenopausal state ($\geq$12 continuous months of amenorrhea with no identified cause other than menopause), and has not undergone surgical sterilization (removal of ovaries and/or uterus).
  - Examples of contraceptive methods with a failure rate of <1% per year include bilateral tubal ligation, male sterilization, hormonal contraceptives that inhibit ovulation, hormone-releasing intrauterine devices, and copper intrauterine devices.
  - The reliability of sexual abstinence should be evaluated in relation to the duration of the clinical study and the preferred and usual lifestyle of the patient. Periodic abstinence (e.g., calendar, ovulation, symptothermal, or postovulation methods) and withdrawal are not acceptable methods of contraception.

4.1.2 Exclusion Criteria

Patients who meet any of the following criteria will be excluded from study entry.

- Prior treatment with immunotherapy for any stage NSCLC, including early-stage (neoadjuvant or adjuvant) disease.
- Patients with EGFR-sensitizing mutations and ALK rearrangements are excluded from the study and should be treated according to standard clinical guidelines. Refer to NCCN guidelines for recommendations on molecular testing (NCCN Guidelines 2016).

"Use of this document is governed by the terms of use on the first page of this document."

- Active CNS metastases requiring treatment as determined by computed tomography (CT) or magnetic resonance imaging (MRI) evaluation during screening and prior radiographic assessments
  - Patients with a history of treated asymptomatic CNS metastases are eligible, provided they meet all of the following criteria:

    Only supratentorial and cerebellar metastases allowed (i.e., no metastases to midbrain, pons, medulla, or spinal cord)

    No ongoing requirement for corticosteroids as therapy for CNS disease

    No stereotactic radiation within 7 days or whole-brain radiation within 14 days prior to enrollment

    No evidence of interim progression between the completion of CNS-directed therapy and the screening radiographic study

    Patients with new asymptomatic CNS metastases detected at the screening scan must receive radiation therapy and/or surgery for CNS metastases. Following treatment, these patients may then be eligible without the need for an additional brain scan prior to enrollment, if all other criteria are met.
- Spinal cord compression not definitively treated with surgery and/or radiation, or previously diagnosed and treated spinal cord compression without evidence that disease has been clinically stable for ≥2 weeks prior to enrollment
- Leptomeningeal disease
- Uncontrolled tumor-related pain
  - Patients requiring pain medication must be on a stable regimen at study entry.
  - Symptomatic lesions amenable to palliative radiotherapy (e.g., bone metastases or metastases causing nerve impingement) should be treated prior to enrollment. Patients should be recovered from the effects of radiation. There is no required minimum recovery period.
  - Asymptomatic metastatic lesions whose further growth would likely cause functional deficits or intractable pain (e.g., epidural metastasis that is not currently associated with spinal cord compression) should be considered for loco-regional therapy if appropriate prior to enrollment.
- Uncontrolled pleural effusion, pericardial effusion, or ascites requiring recurrent drainage procedures (once monthly or more frequently)
  - Patients with indwelling catheters (e.g., PleurX®) are allowed.
- Uncontrolled or symptomatic hypercalcemia (>1.5 mmol/L ionized calcium or calcium >12 mg/dL or corrected serum calcium greater than ULN)
  - Patients who are receiving denosumab prior to enrollment must be willing and eligible to discontinue its use and replace it with a bisphosphonate instead while in the study.
- Malignancies other than NSCLC within 5 years prior to enrollment, with the exception of those with a negligible risk of metastasis or death (e.g., expected

"Use of this document is governed by the terms of use on the first page of this document."

5-year OS >90%) treated with an expected curative outcome (such as adequately treated carcinoma in situ of the cervix, basal- or squamous-cell skin cancer, localized prostate cancer treated surgically with curative intent, or ductal carcinoma in situ treated surgically with curative intent)

- Women who are pregnant, lactating, or intending to become pregnant during the study
  - Women who are not postmenopausal (≥12 months of non–therapy-induced amenorrhea) or surgically sterile must have a negative serum pregnancy test result within 14 days prior to initiation of study drug.
- History of severe allergic, anaphylactic, or other hypersensitivity reactions to chimeric or humanized antibodies or fusion proteins
- Known hypersensitivity to biopharmaceuticals produced in Chinese hamster ovary cells or any component of the atezolizumab formulation
- History of autoimmune disease, including but not limited to myasthenia gravis, myositis, autoimmune hepatitis, systemic lupus erythematosus, rheumatoid arthritis, inflammatory bowel disease, vascular thrombosis associated with antiphospholipid syndrome, Wegener's granulomatosis, Sjögren's syndrome, Guillain-Barré syndrome, multiple sclerosis, vasculitis, or glomerulonephritis (see Appendix 7 for a more comprehensive list of autoimmune diseases)
  - Patients with a history of autoimmune-related hypothyroidism on a stable dose of thyroid-replacement hormone may be eligible for this study.
  - Patients with controlled Type 1 diabetes mellitus on a stable insulin regimen are eligible for this study.
  - Patients with eczema, psoriasis, lichen simplex chronicus, or vitiligo with dermatologic manifestations only (e.g., patients with psoriatic arthritis would be excluded) are permitted provided that they meet the following conditions:

    Rash must cover <10% of body surface area

    Disease is well controlled at baseline and only requiring low-potency topical steroids

    No acute exacerbations of underlying condition within the last 12 months requiring treatment with either psoralen plus ultraviolet A radiation, methotrexate, retinoids, biologic agents, oral calcineurin inhibitors, or high-potency or oral steroids.
- History of idiopathic pulmonary fibrosis, organizing pneumonia (e.g., bronchiolitis obliterans), drug-induced pneumonitis, idiopathic pneumonitis, or evidence of active pneumonitis on screening chest CT scan
  - History of radiation pneumonitis in the radiation field (fibrosis) is permitted.
- Positive HIV test
- Active hepatitis B virus (HBV) infection (chronic or acute), defined as having a positive hepatitis B surface antigen (HBsAg) test at screening

"Use of this document is governed by the terms of use on the first page of this document."

- Patients with past or resolved HBV infection, defined as having a negative HBsAg test and a positive total hepatitis B core antibody (HBcAb) test, are eligible for the study. An HBV DNA test must be performed in these patients prior to enrollment.

- Active hepatitis C virus (HCV) infection, defined as having a positive HCV antibody test and a positive HCV RNA test at screening
- Active tuberculosis
- Severe infections within 4 weeks prior to enrollment, including but not limited to hospitalization for complications of infection, bacteremia, or severe pneumonia
- Significant cardiovascular disease, such as New York Heart Association cardiac disease (Class II or greater), myocardial infarction within 3 months prior to enrollment, unstable arrhythmias, or unstable angina
  - Patients with known coronary artery disease, congestive heart failure not meeting the above criteria, or left ventricular ejection fraction <50% must be on a stable medical regimen that is optimized in the opinion of the treating physician, in consultation with a cardiologist if appropriate.
- Major surgical procedure other than for diagnosis within 28 days prior to enrollment or anticipation of need for a major surgical procedure during the course of the study
- Prior allogeneic bone marrow transplantation or solid organ transplant
- Any other diseases, metabolic dysfunction, physical examination finding, or clinical laboratory finding giving reasonable suspicion of a disease or condition that contraindicates the use of an investigational drug or that may affect the interpretation of the results or render the patient at high risk from treatment complications
- Prior treatment with CD137 agonists or immune checkpoint blockade therapies, anti–PD-1, and anti–PD-L1 therapeutic antibodies
- Treatment with systemic immunostimulatory agents (including but not limited to interferons or interleukin 2) within 4 weeks or 5 half-lives of the drug, whichever is longer, prior to enrollment
- Any approved anti-cancer therapy or hormonal therapy within 3 weeks prior to enrollment; the following exceptions are allowed:
  - Hormone-replacement therapy or oral contraceptives
  - TKIs approved for treatment of NSCLC discontinued ≥7 days prior to enrollment; the baseline scan must be obtained after discontinuation of prior TKIs.
- Treatment with any other investigational agent or participation in another clinical study with therapeutic intent within 28 days prior to enrollment
- Received therapeutic oral or IV antibiotics within 2 weeks prior to enrollment
  - Patients receiving prophylactic antibiotics (e.g., for prevention of a urinary tract infection or to prevent chronic obstructive pulmonary disease exacerbation) are eligible.

"Use of this document is governed by the terms of use on the first page of this document."

- Administration of a live, attenuated vaccine within 4 weeks before enrollment or anticipation that such a live attenuated vaccine will be required during the study or for 5 months after the last dose of atezolizumab
  - Influenza vaccination should be given during influenza season only (approximately October to March).
  - Patients must not receive live, attenuated influenza vaccine (e.g., FluMist®) within 4 weeks prior to enrollment, at any time during the study, or for 5 months after the last dose of atezolizumab.
- Treatment with systemic corticosteroids or other systemic immunosuppressive medications (including but not limited to prednisone, dexamethasone, cyclophosphamide, azathioprine, methotrexate, thalidomide, and anti–tumor necrosis factor [TNF] agents) within 2 weeks prior to enrollment
  - Patients who have received acute, low-dose, systemic immunosuppressant medications (e.g., a one-time dose of dexamethasone for nausea) may be enrolled in the study after discussion with and approval by the Medical Monitor.
  - The use of inhaled corticosteroids for chronic obstructive pulmonary disease, mineralocorticoids (e.g., fludrocortisone) for patients with orthostatic hypotension, and low-dose supplemental corticosteroids for adrenocortical insufficiency are allowed.

4.2 METHOD OF TREATMENT ASSIGNMENT AND BLINDING

This is an open-label single-arm study. Patients who meet all of the inclusion criteria and none of the exclusion criteria as assessed during the screening period will be enrolled in the study to receive treatment with atezolizumab.

The screening tests and evaluations will be performed within 28 days prior to Cycle 1, Day 1. Results of standard-of-care tests or examinations performed prior to obtaining informed consent and within 28 days prior to Cycle 1, Day 1 may be used; such tests do not need to be repeated for screening.

After written informed consent has been obtained and eligibility has been established, the study site will obtain the patient's unique identification number by registering the patient in an interactive voice/Web response system (IxRS) on the day of enrollment.

Patients should receive their first dose of study drug on the day of enrollment if possible. If this is not possible, the first dose should occur within 5 business days after the patient is registered in the IxRS.

4.3 STUDY TREATMENT

The investigational medicinal product (IMP) for this study is atezolizumab.

"Use of this document is governed by the terms of use on the first page of this document."

4.3.1 Formulation, Packaging, and Handling

Atezolizumab (MPDL3280A) will be supplied by the Sponsor in a single-use, 20-mL USP/European Pharmacopoeia Type 1 glass vial as a colorless to slightly yellow, sterile, preservative-free clear liquid solution intended for IV administration. The vial is designed to deliver 20 mL (1200 mg) atezolizumab solution but may contain more than the stated volume to enable delivery of the entire 20 mL volume. For information on the formulation and handling of atezolizumab, see the Atezolizumab Pharmacy Manual and Investigator's Brochure.

4.3.2 Dosage, Administration, and Compliance

The dose level of atezolizumab proposed to be tested in this study is 1200 mg (equivalent to an average body weight–based dose of 15 mg/kg) administered by IV infusion every 21 (±3) days.

Administration of atezolizumab will be performed in a setting with emergency medical facilities and staff who are trained to monitor for and respond to medical emergencies.

The initial dose of atezolizumab will be delivered via IV infusion over 60 (±15) minutes. If the first infusion is tolerated without infusion-associated adverse events, the second infusion may be delivered over 30 (±10) minutes. If the 30-minute infusion is well tolerated, all subsequent infusions may be delivered over 30 (±10) minutes. For all infusions, vital signs will be assessed per the local standard of care (see Appendix 1).

Dose modifications to atezolizumab are not permitted.

No premedication will be allowed for the first dose of atezolizumab. Premedication may be administered for Cycles ≥2 at the discretion of the treating physician. The management of infusion-related reactions will be according to severity as follows:

- In the event that a patient experiences a mild (NCI CTCAE Grade 1) infusion-related event, the infusion rate should be reduced to one-half the rate being given at the time of event onset. Once the event has resolved, the investigator should continue to deliver the infusion at the reduced rate for 30 minutes. If tolerated, the infusion rate may then be increased to the original rate.
- In the event that a patient experiences a moderate infusion-related event (NCI CTCAE Grade 2) or flushing, fever, or throat pain, the patient should have his or her infusion immediately interrupted and should receive aggressive symptomatic treatment. The infusion should be restarted only after the symptoms have adequately resolved to the baseline grade. The infusion rate at restart should be one-half of the rate that was in progress at the time of the onset of the infusion-related event.
- For severe or life-threatening infusion-related events (NCI CTCAE Grade 3 or 4), the infusion should be stopped immediately, and aggressive resuscitation and supportive measures should be initiated. Patients experiencing severe or

"Use of this document is governed by the terms of use on the first page of this document."

life-threatening infusion-related events will not receive further infusion and will be further managed as clinically indicated until the event resolves.

See Appendix 5 for anaphylaxis precautions.

Atezolizumab infusions will be administered per the instructions outlined in Table 4.

Table 4 Administration of First and Subsequent Infusions of Atezolizumab

First Infusion	Subsequent Infusions
• No premedication administered for atezolizumab specifically is permitted<br>• Record patient's vital signs (heart rate, respiratory rate, blood pressure, and temperature) within 60 minutes before starting infusion.<br>• Infuse atezolizumab (1200 mg in a 250-mL 0.9% NaCl intravenous infusion bag) over 60 (± 15) minutes.<br>• If clinically indicated, record patient's vital signs (heart rate, respiratory rate, blood pressure, and temperature) during the infusion at 15, 30, 45, and 60 minutes (± 5-minute windows are allowed for all timepoints).<br>• Record patient's vital signs (heart rate, respiratory rate, blood pressure, and temperature) at 30 (± 10) minutes after the infusion.<br>• Patients will be informed about the possibility of delayed post-infusion symptoms and instructed to contact their study physician if they develop such symptoms.	• If patient experienced infusion-related reaction during any previous infusion, premedication with antihistamines may be administered for Cycles ≥2 at the discretion of the treating physician.<br>• Record patient's vital signs (heart rate, respiratory rate, blood pressure, and temperature) within 60 minutes before starting infusion.<br>• If the patient tolerated the first infusion well without infusion-associated adverse events, the second infusion may be delivered over 30 (± 10) minutes.<br>• If no reaction occurs, subsequent infusions may be delivered over 30 (± 10) minutes. Continue to record vital signs within 60 minutes before starting infusion and during and after the infusion, if clinically indicated.<br>• If the patient had an infusion-related reaction during the previous infusion, the subsequent infusion must be delivered over 60 (± 15) minutes. Record patient's vital signs (heart rate, respiratory rate, blood pressure, and temperature) every 15 (± 5) minutes during the infusion if clinically indicated or patient experienced symptoms during the previous infusion.<br>• Record patient's vital signs (heart rate, respiratory rate, blood pressure, and temperature) 30 min (± 10 min) after the infusion if clinically indicated or patient experienced symptoms during previous infusion.

NaCl = sodium chloride.

Guidelines for treatment interruption or discontinuation are provided in Section 5.1.2.

"Use of this document is governed by the terms of use on the first page of this document."

Any overdose or incorrect administration of study drug should be noted on the Study Drug Administration electronic Case Report Form (eCRF). Adverse events associated with an overdose or incorrect administration of study drug should be recorded on the Adverse Event eCRF.

Refer to the Pharmacy Manual for detailed instructions on drug preparation, storage, and administration.

4.3.3 Investigational Medicinal Product Accountability

All IMPs required for completion of this study (atezolizumab) will be provided by the Sponsor where required by local health authority regulations. The study site will acknowledge receipt of IMPs by using the IxRS to confirm the shipment condition and content. Any damaged shipments will be replaced.

IMPs either will be disposed of at the study site according to the study site's institutional standard operating procedure or will be returned to the Sponsor with the appropriate documentation. The site's method of IMP destruction must be agreed to by the Sponsor. The site must obtain written authorization from the Sponsor before any IMP is destroyed, and IMP destruction must be documented on the appropriate form.

Accurate records of all IMPs received at, dispensed from, returned to, and disposed of by the study site should be recorded on the Drug Inventory Log.

4.3.4 Post-Trial Access to Atezolizumab

The Sponsor will evaluate the appropriateness of continuing to provide atezolizumab to patients who are still on therapy at the end of study. Decisions will be made in accordance with the Roche Global Policy on Continued Access to Investigational Medicinal Product, available at the following Web site:

http://www.roche.com/policy_continued_access_to_investigational_medicines.pdf

4.4 CONCOMITANT THERAPY

Concomitant therapy includes any medication (e.g., prescription drugs, over-the-counter drugs, vaccines, herbal or homeopathic remedies, and nutritional supplements) used by a patient from 7 days prior to screening until the study completion/discontinuation visit. All such medications should be reported to the investigator and recorded on the Concomitant Medications eCRF.

4.4.1 Permitted Therapy

Premedication with antihistamines may be administered for any atezolizumab infusions after Cycle 1.

"Use of this document is governed by the terms of use on the first page of this document."

Patients should continue use of maintenance therapy as follows:

- Hormonal therapy with gonadotropin-releasing hormone agonists or antagonists for prostate cancer
- Oral contraceptives
- Hormone-replacement therapy
- Prophylactic or therapeutic anticoagulation therapy (such as low-molecular-weight heparin or warfarin at a stable dose level)
- Palliative radiotherapy (e.g., treatment of known bony metastases), provided it does not interfere with the assessment of tumor target lesions (e.g., the lesion being irradiated is not the only site of disease, because that would render the patient not evaluable for response by tumor assessments according to RECIST v1.1)
  - It is not a requirement to withhold atezolizumab during palliative radiotherapy
- Inactive vaccinations, but not live, attenuated vaccinations, are permitted during the course of the study (i.e., only inactivated forms of the Influenza vaccinations are permitted). Live, attenuated vaccinations are prohibited (see Section 4.4.3).
- Megestrol administered as an appetite stimulant
- Inhaled corticosteroids for chronic obstructive pulmonary disease
- Mineralocorticoids (e.g., fludrocortisone)
- Low-dose corticosteroids for patients with orthostatic hypotension or adrenocortical insufficiency

In general, investigators should manage a patient's care with supportive therapies as clinically indicated per local standards. Patients who experience infusion-associated symptoms may be treated symptomatically with acetaminophen, ibuprofen, diphenhydramine, and/or famotidine or another H2-receptor antagonist per standard practice. Serious infusion-associated events manifested by dyspnea, hypotension, wheezing, bronchospasm, tachycardia, reduced oxygen saturation, or respiratory distress should be managed with supportive therapies as clinically indicated (e.g., supplemental oxygen and β_2-adrenergic agonists) see Appendix 5.

All medications must be recorded on the appropriate Concomitant Medications eCRF.

4.4.2 Cautionary Therapy for Atezolizumab-Treated Patients

Systemic corticosteroids and TNF-α inhibitors may attenuate potential beneficial immunologic effects of treatment with atezolizumab. Therefore, in situations in which systemic corticosteroids or TNF-α inhibitors would be routinely administered, alternatives, including antihistamines, should be considered first by the treating physician. If the alternatives are not feasible, systemic corticosteroids and TNF-α inhibitors may be administered at the discretion of the treating physician except in the case of patients for whom CT scans with contrast are contraindicated (i.e., patients with contrast allergy or impaired renal clearance) (see also Section 4.4.1).

"Use of this document is governed by the terms of use on the first page of this document."

Systemic corticosteroids are recommended, with caution at the discretion of the treating physician, for the treatment of specific adverse events when associated with atezolizumab therapy. Guidelines for the management of immune-mediated adverse events are described in the Investigator's Brochure.

The concomitant use of herbal therapies is not recommended because their pharmacokinetics, safety profiles, and potential drug-drug interactions are generally unknown. Their use for patients on this study is allowed at the discretion of the investigator, however the herbal therapy must have no known interactions with any study treatment nor can it be used specifically for the treatment of cancer (see Section 4.4.3).

4.4.3 Prohibited Therapy

Any concomitant therapy intended for the treatment of cancer, whether health authority–approved or experimental, is prohibited for various time periods prior to starting study drug, depending on the anti-cancer agent (see Section 4.1.2), and during study drug treatment until disease progression is documented and the patient has discontinued study drug. This includes but is not limited to chemotherapy, hormonal therapy, immunotherapy, radiotherapy, investigational agents, or herbal therapy (unless otherwise noted).

The following medications are prohibited while in the study, unless otherwise noted:

- Traditional herbal medicines intended for the treatment of cancer or those with known interactions with the study treatment, because their use may result in unanticipated drug–drug interactions that may cause or confound assessment of toxicity
- Any live, attenuated vaccine (e.g., FluMist®) within 4 weeks prior to Cycle 1, Day 1, at any time during the study, or for 5 months after the last dose of atezolizumab
- Use of corticosteroids to premedicate patients for whom CT scans with contrast are contraindicated (i.e., patients with contrast allergy or impaired renal clearance); in such patients, non-contrast CT scans of the chest and non-contrast CT scans or MRIs of the abdomen and pelvis should be performed.

4.5 STUDY ASSESSMENTS

Please see Appendix 1 for the schedule of activities to be performed during the study.

4.5.1 Informed Consent Forms and Screening Log

Written informed consent for participation in the study must be obtained before any study-related procedures are performed. Informed Consent Forms for enrolled patients and for patients who are not subsequently enrolled will be maintained at the study site.

All screening evaluations must be completed and reviewed to confirm that patients meet all eligibility criteria before enrollment. The investigator will maintain a screening log to

"Use of this document is governed by the terms of use on the first page of this document."

record details of all patients screened and to confirm eligibility or record reasons for screening failure, as applicable.

4.5.2 Medical History and Demographic Data

Medical history includes clinically significant diseases, surgeries, cancer history (including prior cancer therapies and procedures), reproductive status, smoking history, and all medications (e.g., prescription drugs, vaccines, over-the-counter drugs, herbal or homeopathic remedies, and nutritional supplements) used by the patient within 7 days prior to the screening visit.

Demographic data will include age, sex, and self-reported race/ethnicity.

4.5.3 Physical Examinations

A complete physical examination should be performed at screening and should include an evaluation of the head, eyes, ears, nose, and throat, and the cardiovascular, dermatological, musculoskeletal, respiratory, gastrointestinal, and neurological systems. Any abnormality should be recorded on the General Medical History and Baseline Conditions eCRF.

At subsequent visits (or as clinically indicated), limited symptom-directed physical examinations should be performed. Changes from baseline abnormalities should be recorded in patient notes. New or worsened clinically significant abnormalities should be recorded as adverse events on the Adverse Event eCRF.

4.5.4 Vital Signs

Vital signs will include measurements of respiratory rate, heart rate, and systolic and diastolic blood pressures while the patient is in a seated position, and temperature.

Baseline vital sign assessment will be done on Cycle 1, Day 1 prior to the first dose of study drug. All subsequent vital sign assessments (i.e., during study drug cycles and treatment discontinuation visits) will be done per the local standard of care, as outlined in the schedule of activities (see Appendix 1). Specific requirements for the monitoring of vital signs during atezolizumab infusions are provided in Section 4.3.2.

Patients will be informed about the possibility of delayed post-infusion symptoms and instructed to contact their study physician if they develop such symptoms.

4.5.5 Tumor and Response Evaluations

Tumor assessments will be performed at screening and then every 6 weeks (± 7 days) thereafter, regardless of dose delays, for the first 12 months following Cycle 1, Day 1. After 12 months, tumor assessments will be required every 9 weeks (± 7 days) until disease progression or treatment discontinuation. Disease status (complete response [CR], partial response [PR], stable disease, or PD) will be assessed by the investigator with the use of RECIST v1.1 (see Appendix 4).

"Use of this document is governed by the terms of use on the first page of this document."

The same radiographic procedure used at screening should be used throughout the study (e.g., the same contrast protocol for CT scans). Assessments should be performed by the same evaluator, if possible, to ensure internal consistency across visits. Results must be reviewed by the investigator before dosing at the next cycle. In addition, all radiographic images will be sent to an independent review facility to standardize results and interpretation. Further details on radiographic methods are described in the IRF charter.

Bone scans (Technetium-99m) or sodium fluoride positron emission tomography (NaF-PET) may be performed at screening if clinically indicated. If bone metastases are present at screening and cannot be seen on CT or MRI scans, Technetium-99m or NaF-PET bone scans should be repeated at the discretion of the investigator when complete response is identified in the target disease or when progression in bone is suspected.

Patients who continue treatment beyond radiographic disease progression assessed per RECIST v1.1 should be monitored with a follow-up scan at the next scheduled tumor assessment when the scan frequency is every 6 weeks (± 7 days). If the scan frequency is every 9 weeks, the follow-up scan is recommended at 6 weeks (± 7 days) or earlier if clinically indicated, or per local standard of care.

For patients who discontinue study drug for any reason other than radiographic PD per RECIST v1.1, tumor assessments should continue at the same frequency as would have been followed if the patient had remained on the study drug until radiographic disease progression per RECIST v1.1 (or loss of clinical benefit for patients who continued treatment after disease progression according to RECIST v1.1), withdrawal of consent, death, or study termination by the Sponsor, whichever occurs first.

At the investigator’s discretion, tumor assessments may be repeated at any time if PD is suspected.

4.5.6 Laboratory, Biomarker, and Other Biological Samples

4.5.6.1 Samples for Safety Laboratory Tests

Samples for the following safety laboratory tests will be sent to the study site's local laboratory for analysis:

- Hematology (CBC, including RBC count, hemoglobin, hematocrit, WBC count with differential [neutrophils, eosinophils, lymphocytes, monocytes, basophils, and other cells], and platelet count)
- Serum chemistries (glucose, BUN or urea, creatinine, sodium, potassium, magnesium, chloride, bicarbonate or total CO_2, calcium, phosphorus, total and direct bilirubin, ALT, AST, alkaline phosphatase, LDH, total protein, and albumin)
- Coagulation (aPTT or INR)
- For all women of childbearing potential (including those who have had a tubal ligation), a serum pregnancy test at screening. Urine pregnancy tests will be

"Use of this document is governed by the terms of use on the first page of this document."

performed at specified subsequent visits (see Appendix 1). If a urine pregnancy test is positive, it must be confirmed by a serum pregnancy test.

- Urinalysis (specific gravity, pH, glucose, protein, ketones, and blood); dipstick permitted
- Thyroid function testing (thyroid-stimulating hormone, free T3, free T4)
- HBV serology: HBsAg, total HBcAb
  - If a patient has a negative HbsAg test and a positive total HBcAb test at screening, an HBV DNA test should be performed at screening.
- HCV serology: HCV antibody and (if HCV antibody test is positive) HCV RNA
  - If a patient has a positive HCV antibody test at screening, an HCV RNA test must be performed to determine if the patient has an active HCV infection.
- HIV testing
  - All patients will be tested for HIV prior to inclusion in the study; HIV-positive patients will be excluded.
- EGFR testing
  - If EGFR testing is not available as a part of standard-of-care diagnostic workup, contact the Medical Monitor for additional options. (See Section 4.1.2 for exclusion criteria).

4.5.6.2 Biomarker Samples

A central laboratory will coordinate the sample collection of tissue and blood samples for research-related testing at central laboratories or at the Sponsor. Instruction manuals and supply kits will be provided for all central laboratory assessments. Samples for the following laboratory tests will be sent to one or several central laboratories or to the Sponsor for analysis:

- Biomarker assays in blood samples
  - Blood samples will be obtained for biomarker evaluation (including but not limited to biomarkers related to NSCLC or tumor immune biology) from all eligible patients according to the schedule in Appendix 2. Samples will be processed to obtain plasma and PBMCs for the determination of changes in blood-based biomarkers (e.g., ctDNA). Whole-blood samples may be processed to obtain their derivatives (e.g., RNA and DNA) and evaluated for immune-related, tumor type–related, and other exploratory biomarkers (e.g., alterations in gene expression or single-nucleotide polymorphisms).
  - Any remaining samples collected for biomarker assays may be used for exploratory biomarker profiling, identification, and pharmacodynamic assay development purposes as appropriate.
- Optional collection of tumor tissue
  - Although this study includes all otherwise-eligible patients irrespective of tissue-related biomarker status, tissue biopsies (tissue blocks preferred) may be submitted at any time during the study, with biopsies from diagnosis (archival or

"Use of this document is governed by the terms of use on the first page of this document."

fresh) and at progression preferred, for exploratory biomarker and correlative analysis. If tissue samples are submitted, NGS may be performed by Foundation Medicine. If performed by Foundation Medicine, the investigator can obtain results from these analyses in the form of an NGS report, which is available upon request directly from Foundation Medicine. The investigator may share and discuss the results with the patient, unless the patient chooses otherwise. The Foundation Medicine NGS assay, FoundationOne®, has not been cleared or approved by health authorities. The NGS report is generated for research purposes and is not provided for the purpose of guiding future treatment decisions (see Section 4.5.7). Results may not be available for samples that do not meet testing criteria

– Representative formalin-fixed paraffin-embedded tumor specimens in paraffin blocks (blocks preferred) from a recent (the most recent diagnosis of metastatic NSCLC) biopsy specimen with a minimum volume of 0.2mm^3 viable tumor tissue can be provided at any time during the study. Tissue quality should be confirmed by a local pathologist and should adhere to the Foundation Medicine Pathology Review Process for Optimal Tissue Utilization.

– Fine-needle aspiration, brushing, cell pellet from pleural effusion, bone metastases, and lavage samples are not acceptable. For core-needle biopsy specimens, at least 3 cores should be submitted for evaluation.

– Patients who do not have tissue specimens meeting eligibility requirements may still be eligible for enrollment provided all other eligibility criteria are met. Acceptable samples include core needle biopsies for deep tumor tissue (minimum 3 cores) or excisional, incisional, punch, or forceps biopsies for cutaneous, subcutaneous, or mucosal lesions.

For patients who consent to the optional collection of samples for the Research Biosample Repository (RBR), any leftover material from the above sample collection will be stored and used for exploratory analyses as indicated in Section 4.5.7.

Exploratory biomarker research may include, but will not be limited to, the biomarkers listed in Appendix 2.

For sampling procedures, storage conditions, and shipment instructions, see the laboratory manual.

Unless the patient gives specific consent for his or her leftover samples to be stored for optional exploratory research (see Section 4.5.7), biological samples will be destroyed when the final Clinical Study Report has been completed, with the following exception(s):

- PBMCs and plasma and tumor tissue samples collected for biomarker analysis will be destroyed no later than 15 years after the final Clinical Study Report has been completed.

"Use of this document is governed by the terms of use on the first page of this document."

When a patient withdraws from the study, samples collected prior to the date of withdrawal may still be analyzed, unless the patient specifically requests that the samples be destroyed or local laws require destruction of the samples.

Data arising from sample analysis, including data on germline mutations, will be subject to the confidentiality standards described in Section 4.5.7.4.

4.5.7 Optional Samples for Research Biosample Repository

4.5.7.1 Overview of the Research Biosample Repository

The RBR is a centrally administered group of facilities used for the long-term storage of human biological specimens, including body fluids, solid tissues, and derivatives thereof (e.g., DNA, RNA, proteins, and peptides). The collection, storage, and analysis of RBR specimens will facilitate the rational design of new pharmaceutical agents and the development of diagnostic tests, which may allow for individualized drug therapy for patients in the future.

Specimens for the RBR will be collected from patients who give specific consent to participate in this optional research. RBR specimens will be used to achieve the following objectives:

- To study the association of biomarkers with efficacy, adverse events, or disease progression
- To increase the knowledge and understanding of disease biology
- To study drug response, including drug effects and the processes of drug absorption and disposition
- To develop biomarker or diagnostic assays and establish the performance characteristics of these assays

4.5.7.2 Approval by the Institutional Review Board or Ethics Committee

Collection and submission of biological samples to the RBR is contingent upon the review and approval of the exploratory research and the RBR portion of the Informed Consent Form by each site's Institutional Review Board (IRB) or Ethics Committee (EC) and, if applicable, an appropriate regulatory body. If a site has not been granted approval for RBR sampling, this section of the protocol (Section 4.5.7) will not be applicable at that site.

4.5.7.3 Sample Collection

The following samples will be stored in the RBR and used for research purposes, including, but not limited to, research on biomarkers related to atezolizumab or diseases:

- Blood samples and/or plasma and/or PMBCs collected at screening; on Day 1 of Cycles 1, 2, 3 and 4; and at first evidence of radiographic progression or loss of clinical benefit

"Use of this document is governed by the terms of use on the first page of this document."

- Tumor (e.g., primary tumor, metastatic site, or site of local recurrence or advancement, if appropriate) samples collected at diagnosis or later
- Leftover blood, serum, plasma, PBMC, and tumor tissue samples

The above samples may be sent to one or more laboratories for DNA extraction to enable analysis of germline mutations or somatic mutations via whole genome sequencing (WGS), whole exome sequencing (WES), NGS, or other genomic analysis methods.

Genomics is increasingly informing researchers' understanding of disease pathobiology. WGS and WES provide a comprehensive characterization of the genome and exome, respectively, along with clinical data collected in this study, may increase the opportunity for developing new therapeutic approaches. Data will be analyzed in the context of this study, but will also be explored in aggregate with data from other studies. The availability of a larger dataset will assist in the identification of important pathways, guiding the development of new targeted agents.

For sampling procedures, storage conditions, and shipment instructions, see the laboratory manual.

RBR specimens are to be stored until they are no longer needed or until they are exhausted. However, the RBR storage period will be in accordance with the IRB/EC–approved Informed Consent Form and applicable laws (e.g., health authority requirements).

4.5.7.4 Confidentiality

Specimens and associated data will be labeled with a unique patient identification number.

Patient medical information associated with RBR specimens is confidential and may be disclosed to third parties only as permitted by the Informed Consent Form (or separate authorization for use and disclosure of personal health information) signed by the patient, unless permitted or required by law.

Given the complexity and exploratory nature of the analyses, data derived from RBR specimens will generally not be provided to study investigators or patients unless required by law. The aggregate results of any conducted research will be available in accordance with the effective Sponsor policy on study data publication.

Data generated from RBR specimens must be available for inspection upon request by representatives of national and local health authorities and Sponsor monitors, representatives, and collaborators, as appropriate.

"Use of this document is governed by the terms of use on the first page of this document."

Any inventions and resulting patents, improvements, and/or know-how originating from the use of the RBR data will become and remain the exclusive and unburdened property of the Sponsor, except where agreed otherwise.

4.5.7.5 Consent to Participate in the Research Biosample Repository

The Informed Consent Form will contain a separate section that addresses participation in the RBR. The investigator or authorized designee will explain to each patient the objectives, methods, and potential hazards of participation in the RBR. Patients will be told that they are free to refuse to participate and may withdraw their specimens at any time and for any reason during the storage period. A separate, specific signature will be required to document a patient's agreement to provide optional RBR specimens. Patients who decline to participate will not provide a separate signature.

The investigator should document whether or not the patient has given consent to participate and (if applicable) the date(s) of consent, by completing the RBR Research Sample Informed Consent eCRF.

In the event of an RBR participant's death or loss of competence, the participant's specimens and data will continue to be used as part of the RBR research.

A separate, specific signature is not required for the following mandatory RBR samples:

- Blood taken at screening, baseline, and progression

4.5.7.6 Withdrawal from the Research Biosample Repository

Patients who give consent to provide RBR specimens have the right to withdraw their specimens from the RBR at any time for any reason. If a patient wishes to withdraw consent to the testing of his or her specimens, the investigator must inform the Medical Monitor in writing of the patient's wishes through use of the appropriate RBR Subject Withdrawal Form and, if the study is ongoing, must enter the date of withdrawal on the RBR Research Sample Withdrawal of Informed Consent eCRF. The patient will be provided with instructions on how to withdraw consent after the study is closed. A patient's withdrawal from Study ML39237 does not, by itself, constitute withdrawal of specimens from the RBR. Likewise, a patient's withdrawal from the RBR does not constitute withdrawal from Study ML39237.

4.5.7.7 Monitoring and Oversight

RBR specimens will be tracked in a manner consistent with Good Clinical Practice by a quality-controlled, auditable, and appropriately validated laboratory information management system to ensure compliance with data confidentiality, as well as adherence to authorized use of specimens as specified in this protocol and in the Informed Consent Form. Sponsor monitors and auditors will have direct access to appropriate parts of records relating to patient participation in the RBR for the purposes of verifying the data provided to the Sponsor. The site will permit monitoring, audits,

"Use of this document is governed by the terms of use on the first page of this document."

IRB/EC review, and health authority inspections by providing direct access to source data and documents related to the RBR samples.

4.5.8 On-Study Survival Follow-Up

While in the study, patients will be followed for survival and anti-cancer therapy. Survival follow-up assessments will occur at specified intervals until study completion or discontinuation. Assessments will include survival status, date of death, cause of death, and any new anti-cancer treatment.

4.5.9 Optional Long-Term Follow-Up

Patients enrolled in the study will be asked to participate in optional long-term follow-up, which will include collection of long-term survival data beyond the end of the study. This long-term data collection will be managed by the Sponsor's designee. The patient may withdraw consent from optional long-term follow-up at any time during or after the study by e-mail.

Long-term follow-up will be initiated after the study end or if the study is terminated for any reason. *Survival data will be collected during the optional long-term follow-up period every 3 months for the first year, then every 6 months thereafter for up to 5 years from enrollment of the first patient into the study, until the last patient dies, withdraws consent, or is lost to follow-up, whichever occurs first.*

4.5.10 ECOG Performance Status

ECOG performance status (see Appendix 6) will be measured at the visits specified in Appendix 1.

4.6 PATIENT, TREATMENT, STUDY, AND SITE DISCONTINUATION

4.6.1 Patient Discontinuation

Patients have the right to voluntarily withdraw from the study at any time for any reason. In addition, the investigator has the right to withdraw a patient from the study at any time. Reasons for withdrawal from the study may include, but are not limited to, the following:

- Patient withdrawal of consent at any time
- Any medical condition that the investigator or Sponsor determines may jeopardize the patient's safety if he or she continues in the study
- Investigator or Sponsor determines that it is in the best interest of the patient
- Patient non-compliance
- Patient is lost to follow-up

Every effort should be made to obtain information on patients who withdraw from the study. The primary reason for withdrawal from the study should be documented on the appropriate eCRF. However, patients will not be followed for any reason after consent has been withdrawn. Patients who withdraw from the study will not be replaced.

"Use of this document is governed by the terms of use on the first page of this document."

4.6.2 Study Treatment Discontinuation

Patients must discontinue study drug if they experience any of the following (but will remain in the study and be evaluated for safety and efficacy):

- Pregnancy
- Intolerable toxicity related to study drug
- Any medical condition that may jeopardize the patient's safety if he or she continues with study drug
- Use of another systemic anti-cancer therapy (see Section 4.1.2)
- Radiographic disease progression per RECIST v1.1
  - **Exception:** Patients will be permitted to continue atezolizumab after RECIST v1.1 criteria for PD are met if they meet all of the following criteria:

    Evidence of clinical benefit (defined as the stabilization or improvement of disease-related symptoms) as assessed by the investigator

    Absence of symptoms and signs (including worsening of laboratory values [e.g., new or worsening hypercalcemia]) indicating unequivocal progression of disease

    No decline in ECOG Performance Status that can be attributed to disease progression

    Absence of tumor progression at critical anatomical sites (e.g., leptomeningeal disease) that cannot be readily managed and stabilized by protocol-allowed medical interventions prior to repeat dosing

When a patient discontinues study drug, regardless of the reason for discontinuation, the patient will be asked to return to the clinic within 30 days after the last dose of study drug for a study drug discontinuation visit. The visit at which the decision is made to discontinue treatment (e.g., loss of clinical benefit is confirmed or disease progression occurs) may be used as the study-drug discontinuation visit. The primary reason for study drug discontinuation should be documented on the appropriate eCRF. Patients who discontinue study drug prematurely will not be replaced.

4.6.3 Study and Site Discontinuation

The Sponsor has the right to terminate this study at any time. Reasons for terminating the study may include, but are not limited to, the following:

- The incidence or severity of adverse events in this or other studies indicates a potential health hazard to patients.
- Patient enrollment is unsatisfactory.
- The prespecified futility analysis is met (see Section 6.7)

The Sponsor will notify the investigator if the Sponsor decides to discontinue the study.

"Use of this document is governed by the terms of use on the first page of this document."

The Sponsor has the right to close a site at any time. Reasons for closing a site may include, but are not limited to, the following:

- Excessively slow recruitment
- Poor protocol adherence
- Inaccurate or incomplete data recording
- Non-compliance with the International Conference on Harmonisation (ICH) guideline for Good Clinical Practice
- No study activity (i.e., all patients have completed the study and all obligations have been fulfilled)

5. ASSESSMENT OF SAFETY

5.1 SAFETY PLAN

Atezolizumab is approved in the United States for the treatment of locally advanced or metastatic UC. The safety plan for patients in this study is based on clinical experience with atezolizumab in completed and ongoing studies. The anticipated important safety risks are outlined below (see Section 5.1.1).

Measures will be taken to ensure the safety of patients participating in this study, including the use of stringent inclusion and exclusion criteria and close monitoring of patients during the study. Administration of atezolizumab will be performed in a monitored setting in which there is immediate access to trained personnel and adequate equipment and medicine to manage potentially serious reactions. After initiation of study drug, all adverse events will be reported until 30 days after the last dose of study drug or until initiation of new anti-cancer therapy, whichever occurs first, and serious adverse events and adverse events of special interest will continue to be reported until 90 days after the last dose of study drug or until initiation of new anti-cancer therapy, whichever occurs first. Guidelines for managing anticipated adverse events, including criteria for dosage modification and treatment interruption or discontinuation, are provided below (see Section 5.1.2). Refer to Section 5.2, Section 5.3, Section 5.4, Section 5.5, and Section 5.6 for details on safety reporting during the study.

5.1.1 Risks Associated with Atezolizumab

Atezolizumab has been associated with risks such as the following: infusion-related reactions and immune-related hepatitis, pneumonitis, colitis, pancreatitis, diabetes mellitus, hypothyroidism, hyperthyroidism, adrenal insufficiency, hypophysitis, Guillain-Barré syndrome, myasthenic syndrome or myasthenia gravis, meningoencephalitis, and myocarditis.

Refer to the Atezolizumab Investigator's Brochure for a detailed description of anticipated safety risks for atezolizumab.

"Use of this document is governed by the terms of use on the first page of this document."

Systemic immune activation is a rare condition characterized by an excessive immune response. Given the mechanism of action of atezolizumab, systemic immune activation is considered a potential risk when given in combination with other immunomodulating agents. Systemic immune activation should be included in the differential diagnosis for patients who, in the absence of an alternative etiology, develop a sepsis-like syndrome after administration of atezolizumab.

The initial evaluation should include the following:

- CBC with peripheral smear
- PT, PTT, fibrinogen, and D-dimer
- Ferritin
- Triglycerides
- AST, ALT, and total bilirubin
- LDH
- Complete neurologic and abdominal examination (assess for hepatosplenomegaly)

If systemic immune activation is still suspected after the initial evaluation, contact the Medical Monitor for additional recommendations.

5.1.2 Management of Patients Who Experience Specific Adverse Events

There will be no dose modifications for atezolizumab in this study.

Study treatment may be temporarily suspended in patients experiencing toxicity that is considered to be related to study drug. If atezolizumab is withheld for >105 days, the patient will be discontinued from atezolizumab. If the investigator believes the patient is likely to derive clinical benefit and the Medical Monitor is in agreement, atezolizumab treatment can be resumed after being withheld for >105 days. If a patient must be tapered off corticosteroids used to treat adverse events, study drug may be withheld for >105 days. The investigator and the Medical Monitor will determine the acceptable length of treatment interruption.

Dose interruptions for reasons other than toxicity (e.g., surgical procedures) may be allowed with Medical Monitor approval. The investigator and the Medical Monitor will determine the acceptable length of treatment interruption.

Guidelines for management of patients who experience specific adverse events associated with atezolizumab are provided in the Atezolizumab Investigator's Brochure and in Appendix 8.

"Use of this document is governed by the terms of use on the first page of this document."

5.2 SAFETY PARAMETERS AND DEFINITIONS

Safety assessments will consist of monitoring and recording adverse events, including serious adverse events and adverse events of special interest, performing protocol-specified safety laboratory assessments, measuring protocol-specified vital signs, and conducting other protocol-specified tests that are deemed critical to the safety evaluation of the study.

Certain types of events require immediate reporting to the Sponsor, as outlined in Section 5.4.

5.2.1 Adverse Events

According to the ICH guideline for Good Clinical Practice, an adverse event is any untoward medical occurrence in a clinical investigation subject administered a pharmaceutical product, regardless of causal attribution. An adverse event can therefore be any of the following:

- Any unfavorable and unintended sign (including an abnormal laboratory finding), symptom, or disease temporally associated with the use of a medicinal product, whether or not considered related to the medicinal product
- Any new disease or exacerbation of an existing disease (a worsening in the character, frequency, or severity of a known condition), except as described in Section 5.3.5.10
- Recurrence of an intermittent medical condition (e.g., headache) not present at baseline
- Any deterioration in a laboratory value or other clinical test (e.g., ECG, X-ray) that is associated with symptoms or leads to a change in study drug or concomitant therapy or discontinuation from study drug
- Adverse events that are related to a protocol-mandated intervention, including those that occur prior to assignment of study drug (e.g., screening invasive procedures such as biopsies)

5.2.2 Serious Adverse Events (Immediately Reportable to the Sponsor)

A serious adverse event is any adverse event that meets any of the following criteria:

- Is fatal (i.e., the adverse event actually causes or leads to death)
- Is life threatening (i.e., the adverse event, in the view of the investigator, places the patient at immediate risk of death)
  - This does not include any adverse event that, had it occurred in a more severe form or was allowed to continue, might have caused death.
- Requires or prolongs inpatient hospitalization (see Section 5.3.5.11)

"Use of this document is governed by the terms of use on the first page of this document."

- Results in persistent or significant disability/incapacity (i.e., the adverse event results in substantial disruption of the patient's ability to conduct normal life functions)
- Is a congenital anomaly/birth defect in a neonate/infant born to a mother exposed to study drug
- Is a significant medical event in the investigator's judgment (e.g., may jeopardize the patient or may require medical/surgical intervention to prevent one of the outcomes listed above)

The terms "severe" and "serious" are not synonymous. Severity refers to the intensity of an adverse event (e.g., rated as mild, moderate, or severe, or according to the NCI CTCAE; see Section 5.3.3); the event itself may be of relatively minor medical significance (such as severe headache without any further findings).

Severity and seriousness need to be independently assessed for each adverse event recorded on the eCRF.

Serious adverse events are required to be reported by the investigator to the Sponsor immediately (i.e., no more than 24 hours after learning of the event; see Section 5.4.2 for reporting instructions).

5.2.3 Adverse Events of Special Interest (Immediately Reportable to the Sponsor)

Adverse events of special interest are required to be reported by the investigator to the Sponsor immediately (i.e., no more than 24 hours after learning of the event; see Section 5.3.5.4 for reporting instructions). Adverse events of special interest for this study include the following:

- Any of the following confirmed treatment-emergent autoimmune conditions:
  - Pneumonitis
  - Colitis
  - Endocrinopathies: diabetes mellitus, pancreatitis, or adrenal insufficiency or hyperthyroidism
  - Hepatitis
  - Transaminitis: Grade ≥2 (AST or ALT $>3\times$ ULN and bilirubin $>2\times$ ULN) OR AST or ALT $>10\times$ ULN
  - Systemic lupus erythematosus
  - Neurologic: Guillain-Barré syndrome, myasthenia gravis, meningoencephalitis
  - Nephritis
- Events suggestive of hypersensitivity, cytokine release, influenza-like illness, systemic inflammatory response system, systemic immune activation, or infusion-reaction syndromes

"Use of this document is governed by the terms of use on the first page of this document."

- Cases of potential drug-induced liver injury that include an elevated ALT or AST in combination with either an elevated bilirubin or clinical jaundice, as defined by Hy's law (see Section 5.3.5.7)
- Suspected transmission of an infectious agent by the study drug, as defined below:
  - Any organism, virus, or infectious particle (e.g., prion protein transmitting transmissible spongiform encephalopathy), pathogenic or non-pathogenic, is considered an infectious agent. A transmission of an infectious agent may be suspected from clinical symptoms or laboratory findings that indicate an infection in a patient exposed to a medicinal product. This term applies only when a contamination of the study drug is suspected.

5.3 METHODS AND TIMING FOR CAPTURING AND ASSESSING SAFETY PARAMETERS

The investigator is responsible for ensuring that all adverse events (see Section 5.2.1 for definition) are recorded on the Adverse Event eCRF and reported to the Sponsor in accordance with instructions provided in this section and in Section 5.4, Section 5.5, and Section 5.6.

For each adverse event recorded on the Adverse Event eCRF, the investigator will make an assessment of seriousness (see Section 5.2.2 for seriousness criteria), severity (see Section 5.3.3), and causality (see Section 5.3.4).

5.3.1 Adverse Event Reporting Period

Investigators will seek information on adverse events at each patient contact. All adverse events, whether reported by the patient or noted by study personnel, will be recorded in the patient's medical record and on the Adverse Event eCRF.

After informed consent has been obtained **but prior to initiation of study drug**, only serious adverse events caused by a protocol-mandated intervention (e.g., invasive procedures such as biopsies, discontinuation of medications) should be reported (see Section 5.4.2 for instructions for reporting serious adverse events).

After initiation of study drug, all adverse events will be reported until 30 days after the last dose of study drug or until initiation of new anti-cancer therapy, whichever occurs first, and serious adverse events and adverse events of special interest will continue to be reported until 90 days after the last dose of study drug or until initiation of new anti-cancer therapy, whichever occurs first.

For patients participating in the optional long term follow-up (as outlined in Section 4.5.9) who continue to receive atezolizumab, all AE's will be reported until 30 days after the last dose of atezolizumab during this period of the study. Additionally, serious adverse events and non-serious adverse events of special interest will be reported until 90 days after the last dose of atezolizumab.

"Use of this document is governed by the terms of use on the first page of this document."

Female patients of childbearing potential will be instructed to immediately inform the investigator if they become pregnant during the optional long-term follow-up period or within 5 months after receiving their last dose of atezolizumab.

Pregnancies are to be reported to the Sponsor, as applicable, according to the same guidelines as outlined in Section 5.4.3.1.

5.3.2 Eliciting Adverse Event Information

A consistent methodology of non-directive questioning should be adopted for eliciting adverse event information at all patient evaluation timepoints. Examples of non-directive questions include the following:

"How have you felt since your last clinic visit?"

"Have you had any new or changed health problems since you were last here?"

5.3.3 Assessment of Severity of Adverse Events

The adverse event severity grading scale for NCI CTCAE v4.0 will be used for assessing adverse event severity. Table 5 will be used for assessing severity for adverse events that are not specifically listed in the NCI CTCAE.

"Use of this document is governed by the terms of use on the first page of this document."

Table 5 Adverse Event Severity Grading Scale for Events Not Specifically Listed in the NCI CTCAE

Grade	Severity
1	Mild; asymptomatic or mild symptoms; clinical or diagnostic observations only; or intervention not indicated
2	Moderate; minimal, local, or non-invasive intervention indicated; or limiting age-appropriate instrumental activities of daily living [a]
3	Severe or medically significant, but not immediately life-threatening; hospitalization or prolongation of hospitalization indicated; disabling; or limiting self-care activities of daily living [b,c]
4	Life-threatening consequences or urgent intervention indicated [d]
5	Death related to adverse event [d]

NCI CTCAE = National Cancer Institute Common Terminology Criteria for Adverse Events.

Note: Based on the most recent version of the NCI CTCAE (v 4.0), which can be found at: http://ctep.cancer.gov/protocolDevelopment/electronic_applications/ctc.htm

[a] Instrumental activities of daily living refers to preparing meals, shopping for groceries or clothes, using the telephone, managing money, etc.

[b] Examples of self-care activities of daily living include bathing, dressing and undressing, feeding oneself, using the toilet, and taking medications, as performed by patients who are not bedridden.

[c] If an event is assessed as a "significant medical event," it must be reported as a serious adverse event (see Section 5.4.2 for reporting instructions), per the definition of serious adverse event in Section 5.2.2.

[d] Grade 5 events must be reported as serious adverse events (see Section 5.4.2 for reporting instructions), per the definition of serious adverse event in Section 5.2.2.

5.3.4 Assessment of Causality of Adverse Events

Investigators should use their knowledge of the patient, the circumstances surrounding the event, and an evaluation of any potential alternative causes to determine whether an adverse event is considered to be related to the study drug, indicating "yes" or "no" accordingly. The following guidance should be taken into consideration (see also Table 6):

- Temporal relationship of event onset to the initiation of study drug
- Course of the event, with special consideration of the effects of dose reduction, discontinuation of study drug, or reintroduction of study drug (as applicable)
- Known association of the event with the study drug or with similar treatments
- Known association of the event with the disease under study
- Presence of risk factors in the patient or use of concomitant medications known to increase the occurrence of the event
- Presence of non–treatment-related factors that are known to be associated with the occurrence of the event

"Use of this document is governed by the terms of use on the first page of this document."

Table 6 Causal Attribution Guidance

Is the adverse event suspected to be caused by the study drug on the basis of facts, evidence, science-based rationales, and clinical judgment?	
YES	There is a plausible temporal relationship between the onset of the adverse event and administration of the study drug, and the adverse event cannot be readily explained by the patient's clinical state, intercurrent illness, or concomitant therapies; and/or the adverse event follows a known pattern of response to the study drug; and/or the adverse event abates or resolves upon discontinuation of the study drug or dose reduction and, if applicable, reappears upon re-challenge.
NO	An adverse event will be considered related, unless it fulfills the criteria specified below. Evidence exists that the adverse event has an etiology other than the study drug (e.g., preexisting medical condition, underlying disease, intercurrent illness, or concomitant medication); and/or the adverse event has no plausible temporal relationship to administration of the study drug (e.g., cancer diagnosed 2 days after first dose of study drug).

For patients receiving combination therapy, causality will be assessed individually for each protocol-mandated therapy.

5.3.5 Procedures for Recording Adverse Events

Investigators should use correct medical terminology/concepts when recording adverse events on the Adverse Event eCRF. Avoid colloquialisms and abbreviations.

Only one adverse event term should be recorded in the event field on the Adverse Event eCRF.

5.3.5.1 Infusion-Related Reactions

Adverse events that occur during or within 24 hours after study drug administration and are judged to be related to study drug infusion should be captured as a diagnosis (e.g., "infusion-related reaction" or "anaphylactic reaction") on the Adverse Event eCRF. If possible, avoid ambiguous terms such as "systemic reaction." Associated signs and symptoms should be recorded on the dedicated Infusion-Related Reaction eCRF. If a patient experiences both a local and systemic reaction to the same dose of study drug, each reaction should be recorded separately on the Adverse Event eCRF, with signs and symptoms also recorded separately on the dedicated Infusion-Related Reaction eCRF.

5.3.5.2 Diagnosis versus Signs and Symptoms

For adverse events, a diagnosis (if known) should be recorded on the Adverse Event eCRF rather than individual signs and symptoms (e.g., record only liver failure or hepatitis rather than jaundice, asterixis, and elevated transaminases). However, if a constellation of signs and/or symptoms cannot be medically characterized as a single diagnosis or syndrome at the time of reporting, each individual event should be recorded on the Adverse Event eCRF. If a diagnosis is subsequently established, all previously

"Use of this document is governed by the terms of use on the first page of this document."

reported adverse events based on signs and symptoms should be nullified and replaced by one adverse event report based on the single diagnosis, with a starting date that corresponds to the starting date of the first symptom of the eventual diagnosis.

5.3.5.3 Adverse Events That Are Secondary to Other Events

In general, adverse events that are secondary to other events (e.g., cascade events or clinical sequelae) should be identified by their primary cause, with the exception of severe or serious secondary events. A medically significant secondary adverse event that is separated in time from the initiating event should be recorded as an independent event on the Adverse Event eCRF. For example:

- If vomiting results in mild dehydration with no additional treatment in a healthy adult, only vomiting should be reported on the eCRF.
- If vomiting results in severe dehydration, both events should be reported separately on the eCRF.
- If a severe gastrointestinal hemorrhage leads to renal failure, both events should be reported separately on the eCRF.
- If dizziness leads to a fall and consequent fracture, all three events should be reported separately on the eCRF.
- If neutropenia is accompanied by an infection, both events should be reported separately on the eCRF.

All adverse events should be recorded separately on the Adverse Event eCRF if it is unclear as to whether the events are associated.

5.3.5.4 Persistent or Recurrent Adverse Events

A persistent adverse event is one that extends continuously, without resolution, between patient evaluation timepoints. Such events should only be recorded once on the Adverse Event eCRF. The initial severity (intensity or grade) of the event will be recorded at the time the event is first reported. If a persistent adverse event becomes more severe, the most extreme severity should also be recorded on the Adverse Event eCRF. If the event becomes serious, it should be reported to the Sponsor immediately (i.e., no more than 24 hours after learning that the event became serious; see Section 5.4.2 for reporting instructions). The Adverse Event eCRF should be updated by changing the event from "non-serious" to "serious," providing the date that the event became serious, and completing all data fields related to serious adverse events.

A recurrent adverse event is one that resolves between patient evaluation timepoints and subsequently recurs. Each recurrence of an adverse event should be recorded as a separate event on the Adverse Event eCRF.

"Use of this document is governed by the terms of use on the first page of this document."

5.3.5.5 Abnormal Laboratory Values

Not every laboratory abnormality qualifies as an adverse event. A laboratory test result must be reported as an adverse event if it is a change from baseline and meets any of the following criteria:

- Is accompanied by clinical symptoms
- Results in a change in study drug (e.g., dosage modification, treatment interruption, or treatment discontinuation)
- Results in a medical intervention (e.g., potassium supplementation for hypokalemia) or a change in concomitant therapy
- Is clinically significant in the investigator’s judgment
  - Note: For oncology studies, certain abnormal values may not qualify as adverse events.

It is the investigator’s responsibility to review all laboratory findings. Medical and scientific judgment should be exercised in deciding whether an isolated laboratory abnormality should be classified as an adverse event.

If a clinically significant laboratory abnormality is a sign of a disease or syndrome (e.g., alkaline phosphatase and bilirubin $5 \times$ ULN associated with cholestasis), only the diagnosis (i.e., cholestasis) should be recorded on the Adverse Event eCRF.

If a clinically significant laboratory abnormality is not a sign of a disease or syndrome, the abnormality itself should be recorded on the Adverse Event eCRF, along with a descriptor indicating whether the test result is above or below the normal range (e.g., "elevated potassium," as opposed to "abnormal potassium"). If the laboratory abnormality can be characterized by a precise clinical term per standard definitions, the clinical term should be recorded as the adverse event. For example, an elevated serum potassium level of 7.0 mEq/L should be recorded as "hyperkalemia."

Observations of the same clinically significant laboratory abnormality from visit to visit should only be recorded once on the Adverse Event eCRF (see Section 5.3.5.4 for details on recording persistent adverse events).

5.3.5.6 Abnormal Vital Sign Values

Not every vital sign abnormality qualifies as an adverse event. A vital sign result must be reported as an adverse event if it is a change from baseline and meets any of the following criteria:

- Is accompanied by clinical symptoms
- Results in a change in study drug (e.g., dosage modification, treatment interruption, or treatment discontinuation)
- Results in a medical intervention or a change in concomitant therapy
- Is clinically significant in the investigator’s judgment

"Use of this document is governed by the terms of use on the first page of this document."

It is the investigator's responsibility to review all vital sign findings. Medical and scientific judgment should be exercised in deciding whether an isolated vital sign abnormality should be classified as an adverse event.

If a clinically significant vital sign abnormality is a sign of a disease or syndrome (e.g., high blood pressure), only the diagnosis (i.e., hypertension) should be recorded on the Adverse Event eCRF.

Observations of the same clinically significant vital sign abnormality from visit to visit should only be recorded once on the Adverse Event eCRF (see Section 5.3.5.4 for details on recording persistent adverse events).

5.3.5.7 Abnormal Liver Function Tests

The finding of an elevated ALT or AST (>3 × baseline value) in combination with either an elevated total bilirubin (>2 × ULN) or clinical jaundice in the absence of cholestasis or other causes of hyperbilirubinemia is considered to be an indicator of severe liver injury (as defined by Hy's law). Therefore, investigators must report as an adverse event the occurrence of either of the following:

- Treatment-emergent ALT or AST >3 × baseline value in combination with total bilirubin >2 × ULN (of which ≥35% is direct bilirubin)
- Treatment-emergent ALT or AST >3 × baseline value in combination with clinical jaundice

The most appropriate diagnosis or (if a diagnosis cannot be established) the abnormal laboratory values should be recorded on the Adverse Event eCRF (see Section 5.3.5.2) and reported to the Sponsor immediately (i.e., no more than 24 hours after learning of the event), either as a serious adverse event or an adverse event of special interest (see Section 5.4.2).

5.3.5.8 Deaths

For this protocol, mortality is an efficacy endpoint. Deaths that occur during the protocol-specified adverse event reporting period (see Section 5.3.1) that are attributed by the investigator solely to progression of NSCLC should be recorded on the Study Completion/Study Discontinuation eCRF. All other on-study deaths, regardless of relationship to study drug, must be recorded on the Adverse Event eCRF and immediately reported to the Sponsor (see Section 5.4.2).

Death should be considered an outcome and not a distinct event. The event or condition that caused or contributed to the fatal outcome should be recorded as the single medical concept on the Adverse Event eCRF. Generally, only one such event should be reported. If the cause of death is unknown and cannot be ascertained at the time of reporting, **"unexplained death"** should be recorded on the Adverse Event eCRF. If the cause of death later becomes available (e.g., after autopsy), "unexplained death" should be replaced by the established cause of death. The term "sudden death" should not be

"Use of this document is governed by the terms of use on the first page of this document."

used unless combined with the presumed cause of death (e.g., "sudden cardiac death"). Deaths that occur after the adverse event reporting period should be reported as described in Section 5.6.

5.3.5.9 Preexisting Medical Conditions

A preexisting medical condition is one that is present at the screening visit for this study. Such conditions should be recorded on the General Medical History and Baseline Conditions eCRF.

A preexisting medical condition should be recorded as an adverse event only if the frequency, severity, or character of the condition worsens during the study. When recording such events on the Adverse Event eCRF, it is important to convey the concept that the preexisting condition has changed by including applicable descriptors (e.g., "more frequent headaches").

5.3.5.10 Lack of Efficacy or Worsening of NSCLC

Events that are clearly consistent with the expected pattern of progression of the underlying disease should not be recorded as adverse events. These data will be captured as efficacy assessment data only. In most cases, the expected pattern of progression will be based on RECIST v 1.1 criteria. In rare cases, the determination of clinical progression will be based on symptomatic deterioration. However, every effort should be made to document progression through use of objective criteria. If there is any uncertainty as to whether an event is due to disease progression, it should be reported as an adverse event.

5.3.5.11 Hospitalization or Prolonged Hospitalization

Any adverse event that results in hospitalization (i.e., inpatient admission to a hospital) or prolonged hospitalization should be documented and reported as a serious adverse event (per the definition of serious adverse event in Section 5.2.2), except as outlined below.

An event that leads to hospitalization under the following circumstances should not be reported as an adverse event or a serious adverse event:

- Hospitalization for respite care
- Planned hospitalization required by the protocol (e.g., for study drug administration or to perform an efficacy measurement for the study)
- Hospitalization for a preexisting condition, provided that all of the following criteria are met:
  - The hospitalization was planned prior to the study or was scheduled during the study when elective surgery became necessary because of the expected normal progression of the disease
  - The patient has not experienced an adverse event
- Hospitalization due solely to progression of the underlying cancer

"Use of this document is governed by the terms of use on the first page of this document."

An event that leads to hospitalization under the following circumstances is not considered to be a serious adverse event, but should be reported as an adverse event instead:

- Hospitalization that was necessary because of patient requirement for outpatient care outside of normal outpatient clinic operating hours

5.3.5.12 Adverse Events Associated with an Overdose or Error in Drug Administration

An overdose is the accidental or intentional use of a drug in an amount higher than the dose being studied. An overdose or incorrect administration of study drug is not itself an adverse event, but it may result in an adverse event. All adverse events associated with an overdose or incorrect administration of study drug should be recorded on the Adverse Event eCRF. If the associated adverse event fulfills seriousness criteria, the event should be reported to the Sponsor immediately (i.e., no more than 24 hours after learning of the event; see Section 5.4.2).

No safety data related to overdosing of atezolizumab are available.

5.4 IMMEDIATE REPORTING REQUIREMENTS FROM INVESTIGATOR TO SPONSOR

Certain events require immediate reporting to allow the Sponsor to take appropriate measures to address potential new risks in a clinical trial. The investigator must report such events to the Sponsor immediately; under no circumstances should reporting take place more than 24 hours after the investigator learns of the event. The following is a list of events that the investigator must report to the Sponsor within 24 hours after learning of the event, regardless of relationship to study drug:

- Serious adverse events (see Section 5.4.2 for further details)
- Adverse events of special interest (see Section 5.4.2 for further details)
- Pregnancies (see Section 5.4.3 for further details)

The investigator must report new significant follow-up information for these events to the Sponsor immediately (i.e., no more than 24 hours after becoming aware of the information). New significant information includes the following:

- New signs or symptoms or a change in the diagnosis
- Significant new diagnostic test results
- Change in causality based on new information
- Change in the event’s outcome, including recovery
- Additional narrative information on the clinical course of the event

Investigators must also comply with local requirements for reporting serious adverse events to the local health authority and IRB/EC.

"Use of this document is governed by the terms of use on the first page of this document."

5.4.1 Emergency Medical Contacts

Medical Monitor Contact Information

PRA Health Sciences Medical Monitoring Support Center (North America) Medical Monitor contact information:

Medical Monitor: [redacted], M.D. (Primary Contact)

Telephone Nos.: 1.866.326.5053 (toll-free) or 1.434.951.4082 (direct; USA)

Fax Nos.: 1.800.280.7035 (toll-free) or 1.913.307.5751 (direct; USA)

E-mail: ML39237@prahs.com

Genentech Medical Monitor contact information:

Medical Monitor: [redacted], Pharm.D. (Secondary Contact)

Telephone No.: [redacted] (USA)

E-mail: [redacted]

5.4.2 Reporting Requirements for Serious Adverse Events and Adverse Events of Special Interest

5.4.2.1 Events That Occur prior to Study Drug Initiation

After informed consent has been obtained but prior to initiation of study drug, only serious adverse events caused by a protocol-mandated intervention should be reported. The Serious Adverse Event/Adverse Event of Special Interest Reporting Form provided to investigators should be completed and submitted to the Sponsor or its designee immediately (i.e., no more than 24 hours after learning of the event), either by faxing or by scanning and e-mailing the form by using the fax number or e-mail address provided to investigators.

5.4.2.2 Events That Occur after Study Drug Initiation

After initiation of study drug, serious adverse events and adverse events of special interest will be reported until 90 days after the last dose of study drug or initiation of new anti-cancer therapy, whichever occurs first. All other adverse events, regardless of relationship to study drug, will be reported until 30 days after the last dose of study drug or initiation of new anti-cancer therapy, whichever occurs first.

Investigators should record all case details that can be gathered immediately (i.e., within 24 hours after learning of the event) on the Adverse Event eCRF and submit the report via the electronic data capture (EDC) system. A report will be generated and sent to Safety Risk Management by the EDC system.

In the event that the EDC system is unavailable, the Serious Adverse Event/Adverse Event of Special Interest Reporting Form provided to investigators should be completed and submitted to the Sponsor or its designee immediately (i.e., no more than 24 hours after learning of the event), either by faxing or by scanning and e-mailing the form by using the fax number or e-mail address provided to investigators. Once the EDC system is available, all information will need to be entered and submitted via the EDC system.

"Use of this document is governed by the terms of use on the first page of this document."

Instructions for reporting serious adverse events that occur >90 days after the last dose of study drug are provided in Section 5.6.

5.4.3 Reporting Requirements for Pregnancies

5.4.3.1 Pregnancies in Female Patients

Female patients of childbearing potential will be instructed to immediately inform the investigator if they become pregnant during the study or within 5 months after the last dose of study drug. A Clinical Trial Pregnancy Reporting Form should be completed and submitted to the Sponsor or its designee immediately (i.e., no more than 24 hours after learning of the pregnancy), either by faxing or by scanning and e-mailing the form by using the fax number or e-mail address provided to investigators. Pregnancy should not be recorded on the Adverse Event eCRF. The investigator should discontinue study drug and counsel the patient, discussing the risks of the pregnancy and the possible effects on the fetus. Monitoring of the patient should continue until conclusion of the pregnancy. Any serious adverse events associated with the pregnancy (e.g., an event in the fetus, an event in the mother during or after the pregnancy, or a congenital anomaly/birth defect in the child) should be reported on the Adverse Event eCRF. In addition, the investigator will submit a Clinical Trial Pregnancy Reporting Form when updated information on the course and outcome of the pregnancy becomes available.

5.4.3.2 Congenital Anomalies/Birth Defects and Abortions

Any congenital anomaly/birth defect in a child born to a female patient exposed to study drug should be classified as a serious adverse event, recorded on the Adverse Event eCRF, and reported to the Sponsor immediately (i.e., no more than 24 hours after learning of the event; see Section 5.4.2). Any abortion should be reported in the same fashion (because the Sponsor considers abortions to be medically significant).

5.4.4 Reporting Requirements for Cases of Atezolizumab Accidental Overdose or Medication Error

Accidental overdose and medication error (hereafter collectively referred to as "special situations"), are defined as follows:

- Accidental overdose: accidental administration of a drug in a quantity that is higher than the assigned dose
- Medication error: accidental deviation in the administration of a drug

    In some cases, a medication error may be intercepted prior to administration of the drug.

"Use of this document is governed by the terms of use on the first page of this document."

Special situations are not in themselves adverse events, but may result in adverse events. All special situations associated with atezolizumab, regardless of whether they result in an adverse event, should be recorded on the Adverse Event eCRF and reported to the Sponsor immediately (i.e., no more than 24 hours after learning of the event). Special situations should be recorded as described below:

- Accidental overdose: Enter the drug name and "accidental overdose" as the event term. Check the "Accidental overdose" and "Medication error" boxes.
- Medication error that does not qualify as an overdose: Enter the name of the drug administered and a description of the error (e.g., wrong dose administered, wrong dosing schedule, incorrect route of administration, wrong drug, expired drug administered) as the event term. Check the "Medication error" box.
- Medication error that qualifies as an overdose: Enter the drug name and "accidental overdose" as the event term. Check the "Accidental overdose" and "Medication error" boxes. Enter a description of the error in the additional case details.
- Intercepted medication error: Enter the drug name and "intercepted medication error" as the event term. Check the "Medication error" box. Enter a description of the error in the additional case details.

Each adverse event associated with a special situation should be recorded separately on the Adverse Event eCRF. If the associated adverse event fulfills seriousness criteria, the event should be reported to the Sponsor immediately (i.e., no more than 24 hours after learning of the event; see Section 5.4.2). Adverse events associated with special situations should be recorded as described below for each situation:

- Accidental overdose: Enter the adverse event term. Check the "Accidental overdose" and "Medication error" boxes.
- Medication error that does not qualify as an overdose: Enter the adverse event term. Check the "Medication error" box.
- Medication error that qualifies as an overdose: Enter the adverse event term. Check the "Accidental overdose" and "Medication error" boxes.

As an example, an accidental overdose that resulted in a headache would require the completion of two Adverse Event eCRF pages, one to report the accidental overdose and one to report the headache. The "Accidental overdose" and "Medication error" boxes would need to be checked on both eCRF pages.

5.5 FOLLOW-UP OF PATIENTS AFTER ADVERSE EVENTS

5.5.1 Investigator Follow-Up

The investigator should follow each adverse event until the event has resolved to baseline grade or better, the event is assessed as stable by the investigator, the patient is lost to follow-up, or the patient withdraws consent. Every effort should be made to follow all serious adverse events considered to be related to study drug or trial-related procedures until a final outcome can be reported.

"Use of this document is governed by the terms of use on the first page of this document."

During the study period, resolution of adverse events (with dates) should be documented on the Adverse Event eCRF and in the patient's medical record to facilitate source data verification.

All pregnancies reported during the study should be followed until pregnancy outcome.

5.5.2 Sponsor Follow-Up

For serious adverse events, adverse events of special interest, and pregnancies, the Sponsor or a designee may follow up by telephone, fax, electronic mail, and/or a monitoring visit to obtain additional case details and outcome information (e.g., from hospital discharge summaries, consultant reports, autopsy reports) in order to perform an independent medical assessment of the reported case.

5.6 ADVERSE EVENTS THAT OCCUR AFTER THE ADVERSE EVENT REPORTING PERIOD

The sponsor should be notified if the investigator becomes aware of any serious adverse event or adverse event of special interest that occurs after the end of the adverse event reporting period (defined as 30 days after the last dose of study drug or until initiation of new anti-cancer therapy, whichever occurs first, for adverse events and until 90 days after the last dose of study drug or until initiation of new anti-cancer therapy, whichever occurs first for serious adverse events and adverse events of special interest), if the event is believed to be related to prior study drug treatment. All such events should be reported until the end of study (defined as the date after which all enrolled patients who remain alive have been followed for at least 18 months from the start of study drug.)

The investigator should report these events directly to Roche or its designee, either by faxing or by scanning and e-mailing the Serious Adverse Event/Adverse Event of Special Interest Reporting Form with use of the fax number or e-mail address provided to investigators.

5.7 EXPEDITED REPORTING TO HEALTH AUTHORITIES, INVESTIGATORS, INSTITUTIONAL REVIEW BOARDS, AND ETHICS COMMITTEES

The Sponsor will promptly evaluate all serious adverse events and adverse events of special interest against cumulative product experience to identify and expeditiously communicate possible new safety findings to investigators, IRBs, ECs, and applicable health authorities based on applicable legislation.

To determine reporting requirements for single adverse event cases, the Sponsor will assess the expectedness of these events with the use of the following reference document:

- Atezolizumab Investigator's Brochure

"Use of this document is governed by the terms of use on the first page of this document."

The Sponsor will compare the severity of each event and the cumulative event frequency reported for the study with the severity and frequency reported in the applicable reference document.

Reporting requirements will also be based on the investigator's assessment of causality and seriousness, with allowance for upgrading by the Sponsor as needed.

6. STATISTICAL CONSIDERATIONS AND ANALYSIS PLAN

There will be approximately 150 patients enrolled in the study. Safety analyses will include all patients who receive at least one dose of study drug. Biomarker analyses will include all patients who received at least one dose of study drug and have baseline biomarker assessment. The primary efficacy analysis measured by ORR and the primary biomarker analysis will be performed after all patients complete 6 months of follow-up (i.e., 6 months after the final patient has been enrolled). The final analysis will be performed at the end of the study, 18 months after all patients have been enrolled. *The overall survival analysis will be updated once the long-term follow-up data is available, and will be summarized in a separate report. Safety reporting for patients who remain on study drug will also be included.*

In addition, OS data collected through the external death registry data after study completion during the optional long-term follow-up will be updated periodically (e.g., every 1–2 years) based on data availability.

6.1 DETERMINATION OF SAMPLE SIZE

The sample size for this study is based on the primary efficacy objective and primary biomarker objective.

Based on 150 patients, the maximum half width of the 2-sided 95% CI of the estimated ORR will be within 8%.

Additionally, based on 120 biomarker-evaluable patients (80% of the study population), with a minimum follow-up of 6 months during the primary analysis, and with 28 patients in the smaller biomarker positive or negative group (19% of the study population), the study primary biomarker analysis will have 89% and 79% power to detect PFS difference if the HRs of PFS between the biomarker positive vs. negative group is 0.50 or 0.55, respectively. The above-mentioned power is for a 2-sided test with a significance level of 0.1, assuming a median PFS of 4 months for the subgroup with a shorter median PFS.

6.2 SUMMARIES OF CONDUCT OF STUDY

The number of patients who enroll in, discontinue, or complete the study will be summarized. Reasons for premature study withdrawal will be listed and summarized. Enrollment and major protocol deviations will be listed and evaluated for their potential effects on the interpretation of study results.

"Use of this document is governed by the terms of use on the first page of this document."

6.3 SUMMARIES OF DEMOGRAPHIC AND BASELINE CHARACTERISTICS

Demographic and baseline characteristics (e.g., age, sex) will be summarized by using means, SDs, medians, and ranges for continuous variables, and proportions for categorical variables, as appropriate.

6.4 EFFICACY ANALYSES

The efficacy analysis population will be based on all patients who have received at least one dose of study drug.

6.4.1 Primary Efficacy Endpoint

The primary efficacy endpoint of this study is investigator-assessed ORR, defined as the proportion of patients whose confirmed best overall response is either a PR or CR per RECIST v1.1. For this analysis, patients not meeting these criteria, including patients without at least one post-baseline response assessment, will be considered non-responders. An estimate of the ORR from all patients who received study drug and the 95% CI will be calculated by using the Blaker method.

6.4.2 Secondary Efficacy Endpoints

Secondary efficacy endpoints include OS and investigator (INV)-assessed duration of response (DOR) and PFS per RECIST v1.1.

DOR will be analyzed for the subset of patients who achieved an objective response. DOR is defined as the time from the initial occurrence of documented CR or PR until documented disease progression as determined by the investigator, or death, whichever occurs first. Median and range will be presented for a descriptive summary.

PFS is defined as the time from the first dose of study drug to the time of disease progression or death from any cause during the study, whichever occurs first. Patients who have not experienced disease progression or death at the time of analysis will be censored at the time of the last tumor assessment. Patients with no post-baseline tumor assessment will be censored at the time of first dose.

OS is defined as the time from the first dose of study drug to the time of death from any cause during the study. Patients who are still alive at the time of analysis will be censored at the time of their last study assessment (for active patients) or at the last date known alive (for patients in follow-up).

PFS and OS of patients in this study will be summarized graphically and with descriptive statistics such as median and landmark PFS by using the Kaplan-Meier (K-M) methodology. The Brookmeyer-Crowley methodology will be used to construct the 95% CI for the median PFS and OS (Brookmeyer and Crowley 1982). K-M methods will also be used to estimate landmark PFS, along with the corresponding 95% CI, by using Greenwood’s formula.

"Use of this document is governed by the terms of use on the first page of this document."

6.4.3 Exploratory Efficacy Endpoints

The relationship between efficacy, baseline characteristics, and biomarkers may also be explored.

6.5 SAFETY ANALYSES

Safety will be assessed through summaries of adverse events, including protocol-defined events of special interest, changes in laboratory test results, changes in vital signs, and exposure to study.

Verbatim descriptions of adverse events will be mapped to thesaurus terms using MedDRA. Adverse event data will be listed by study site, patient number, and study day. Events occurring on the day of or after administration of the first dose of treatment will be summarized by thesaurus term, appropriate thesaurus levels, and NCI CTCAE v4.0 grade. Serious adverse events, including deaths, will be listed separately and will be summarized. For events of varying severity, the highest grade will be used in summaries.

Relevant laboratory tests and vital sign (heart rate, respiratory rate, blood pressures, and temperature) data will be displayed by time, with Grade 3 and 4 values identified, where appropriate. Additionally, all laboratory data will be summarized by NCI CTCAE v4.0 grade.

6.6 BIOMARKER ANALYSES

Biomarker analysis population will be based on all patients who have received at least one dose of study drug and have baseline biomarker assessment.

6.6.1 Primary Biomarker Analyses

K-M curves and a log-rank test will be used to evaluate the differences in INV-assessed PFS between mutation positive versus negative groups at various cutoff points. Tests will be two-sided at a significance level of 0.10.

6.6.2 Secondary Biomarker Analyses

K-M curves and a log-rank test will be used to evaluate the differences in INV-assessed PFS between mutation positive versus negative groups at various cutoff points. Descriptive statistics for PFS curves including median PFS time and 6-, 9-, and 12-month PFS probabilities will be estimated for various cutoff points. OS will be analyzed in a similar way as PFS. INV-assessed ORR will also be summarized by various MLs and by immune signature expression level. The Brookmeyer-Crowley methodology will be used to construct the 95% CI for the median. Greenwood's formula will be used to construct the 95% CI for the landmark PFS.

"Use of this document is governed by the terms of use on the first page of this document."

6.6.3 Exploratory Biomarker Analyses

Change in bTMB after immunotherapy will be analyzed. Status of additional circulating biomarkers, including but not limited to expression of immune genes, as well as others related to immunotherapy and NSCLC may be examined at prespecified timepoints. Outcomes with the study drug will be summarized. A historical control cohort may also be used to evaluate outcomes.

The relationship between bTMB and tTMB may be explored by visual data display and correlation coefficient if enough tTMB data is available.

6.7 INTERIM ANALYSES

6.7.1 Planned Interim Analysis

One interim analysis is planned 6 months after approximately one-half of the patients have been enrolled. The purpose of the interim analysis is to provide preliminary results in evaluating the biomarker cutoff and to perform a futility analysis. The futility criteria to stop the study will be met if the true ORR is more likely $\leq$5% rather than $\geq$15%, or if 4 or fewer patients have an objective response from the first 75 enrolled patients. The chance of stopping the study is $>$67% if the true ORR is 5% or less. The chance of stopping the study is $<$1% if the true ORR is 15% or larger.

No conclusions or study alterations/terminations are to be made based on the biomarker interim analysis results. No type-1 error adjustments will be made for the interim analysis.

The study may continue while the interim analysis is being performed.

6.7.2 Optional Interim Analyses

Given the hypothesis-generating nature of this study, the Sponsor may choose to conduct additional interim analyses. The decision to conduct an optional interim analysis and the timing of the analysis will be documented in the Sponsor's trial master file prior to the conduct of the interim analysis. The interim analysis will be performed and interpreted by Sponsor study team personnel.

7. DATA COLLECTION AND MANAGEMENT

7.1 DATA QUALITY ASSURANCE

The Sponsor will supply eCRF specifications for this study. A contract research organization (CRO) will be responsible for data management of this study, including quality checking of the data. Data entered manually will be collected via EDC through the use of eCRFs. Sites will be responsible for data entry into the EDC system. In the event of discrepant data, the CRO will request data clarification from the sites, which the sites will resolve electronically in the EDC system.

"Use of this document is governed by the terms of use on the first page of this document."

The CRO will produce a Data Quality Plan that describes the quality checking to be performed on the data. Central laboratory data will be sent directly to the CRO with the use of the CRO's standard procedures to handle and process the electronic transfer of these data.

The Sponsor will perform oversight of the data management of this study, including approval of the CRO's data management plans and specifications. Data will be periodically transferred electronically from the CRO to the Sponsor, and the Sponsor's standard procedures will be used to handle and process the electronic transfer of these data.

eCRFs and correction documentation will be maintained in the EDC system's audit trail. System backups for data stored at the CRO and records retention for the study data will be consistent with the CRO's standard procedures.

7.2 ELECTRONIC CASE REPORT FORMS

eCRFs are to be completed through the use of a Sponsor-designated EDC system. Sites will receive training and have access to a manual for appropriate eCRF completion. eCRFs will be submitted electronically to the Sponsor and should be handled in accordance with instructions from the Sponsor.

All eCRFs should be completed by designated, trained site staff. eCRFs should be reviewed and electronically signed and dated by the investigator or a designee.

At the end of the study, the investigator will receive patient data for his or her site in a readable format on a compact disc that must be kept with the study records. Acknowledgement of receipt of the compact disc is required.

7.3 SOURCE DATA DOCUMENTATION

Study monitors will perform ongoing source data verification to confirm that critical protocol data (i.e., source data) entered into the eCRFs by authorized site personnel are accurate, complete, and verifiable from source documents.

Source documents (paper or electronic) are those in which patient data are recorded and documented for the first time. They include, but are not limited to, hospital records, clinical and office charts, laboratory notes, memoranda, patient-reported outcomes, evaluation checklists, pharmacy dispensing records, recorded data from automated instruments, copies of transcriptions that are certified after verification as being accurate and complete, microfiche, photographic negatives, microfilm or magnetic media, X-rays, patient files, and records kept at pharmacies, laboratories, and medico-technical departments involved in a clinical trial.

Before study initiation, the types of source documents that are to be generated will be clearly defined in the Trial Monitoring Plan. This includes any protocol data to be

"Use of this document is governed by the terms of use on the first page of this document."

entered directly into the eCRFs (i.e., no prior written or electronic record of the data) and considered source data.

Source documents that are required to verify the validity and completeness of data entered into the eCRFs must not be obliterated or destroyed and must be retained per the policy for retention of records described in Section 7.5.

To facilitate source data verification, the investigators and institutions must provide the Sponsor direct access to applicable source documents and reports for trial-related monitoring, Sponsor audits, and IRB/EC review. The study site must also allow inspection by applicable health authorities.

7.4 USE OF COMPUTERIZED SYSTEMS

When clinical observations are entered directly into a study site's computerized medical record system (i.e., in lieu of original hardcopy records), the electronic record can serve as the source document if the system has been validated in accordance with health authority requirements pertaining to computerized systems used in clinical research. An acceptable computerized data collection system allows preservation of the original entry of data. If original data are modified, the system should maintain a viewable audit trail that shows the original data as well as the reason for the change, name of the person making the change, and date of the change.

7.5 RETENTION OF RECORDS

Records and documents pertaining to the conduct of this study and the distribution of IMP, including eCRFs, Informed Consent Forms, laboratory test results, and medication inventory records, must be retained by the Principal Investigator for at least 15 years after completion or discontinuation of the study or for the length of time required by relevant national or local health authorities, whichever is longer. After that period of time, the documents may be destroyed, subject to local regulations.

No records may be disposed of without the written approval of the Sponsor. Written notification should be provided to the Sponsor prior to transferring any records to another party or moving them to another location.

8. ETHICAL CONSIDERATIONS

8.1 COMPLIANCE WITH LAWS AND REGULATIONS

This study will be conducted in full conformance with the ICH E6 guideline for Good Clinical Practice and the principles of the Declaration of Helsinki, or the laws and regulations of the country in which the research is conducted, whichever affords the greater protection to the individual. The study will comply with the requirements of the ICH E2A guideline (Clinical Safety Data Management: Definitions and Standards for Expedited Reporting). Studies conducted in the United States or under a U.S. Investigational New Drug (IND) application will comply with FDA regulations and

"Use of this document is governed by the terms of use on the first page of this document."

applicable local, state, and federal laws. Studies conducted in the European Union or European Economic Area will comply with the E.U. Clinical Trial Directive (2001/20/EC).

8.2 INFORMED CONSENT

The Sponsor's sample Informed Consent Form (and ancillary sample Informed Consent Forms such as a Child's Informed Assent Form or Mobile Nursing Informed Consent Form, if applicable) will be provided to each site. If applicable, it will be provided in a certified translation of the local language. The Sponsor or its designee must review and approve any proposed deviations from the Sponsor's sample Informed Consent Forms or any alternate consent forms proposed by the site (collectively, the "Consent Forms") before IRB/EC submission. The final IRB/EC–approved Consent Forms must be provided to the Sponsor for health authority submission purposes according to local requirements.

If applicable, the Informed Consent Form will contain separate sections for any optional procedures. The investigator or authorized designee will explain to each patient the objectives, methods, and potential risks associated with each optional procedure. Patients will be told that they are free to refuse to participate and may withdraw their consent at any time for any reason. A separate, specific signature will be required to document a patient's agreement to participate in optional procedures. Patients who decline to participate will not provide a separate signature.

The Consent Forms must be signed and dated by the patient or the patient's legally authorized representative before his or her participation in the study. The case history or clinical records for each patient shall document the informed consent process and that written informed consent was obtained prior to participation in the study.

The Consent Forms should be revised whenever there are changes to study procedures or when new information becomes available that may affect the willingness of the patient to participate. The final revised IRB/EC–approved Consent Forms must be provided to the Sponsor for health authority submission purposes.

Patients must be re-consented to the most current version of the Consent Forms (or to a significant new information/findings addendum in accordance with applicable laws and IRB/EC policy) during their participation in the study. For any updated or revised Consent Forms, the case history or clinical records for each patient shall document the informed consent process and that written informed consent was obtained with the use of the updated/revised Consent Forms for continued participation in the study.

A copy of each signed Consent Form must be provided to the patient or the patient's legally authorized representative. All signed and dated Consent Forms must remain in each patient's study file or in the site file and must be available for verification by study monitors at any time.

"Use of this document is governed by the terms of use on the first page of this document."

Each Consent Form may also include patient authorization to allow use and disclosure of personal health information in compliance with the U.S. Health Insurance Portability and Accountability Act (HIPAA) of 1996. If the site uses a separate Authorization Form for patient authorization for use and disclosure of personal health information under the HIPAA regulations, the review, approval, and other processes outlined above apply, except that IRB review and approval may not be required per study site policies.

8.3 INSTITUTIONAL REVIEW BOARD OR ETHICS COMMITTEE

This protocol, the Informed Consent Forms, any information to be given to the patient, and relevant supporting information must be submitted to the IRB/EC by the Principal Investigator and reviewed and approved by the IRB/EC before the study is initiated. In addition, any patient recruitment materials must be approved by the IRB/EC.

The Principal Investigator is responsible for providing written summaries of the status of the study to the IRB/EC annually or more frequently in accordance with the requirements, policies, and procedures established by the IRB/EC. Investigators are also responsible for promptly informing the IRB/EC of any protocol amendments (see Section 9.6).

In addition to the requirements for reporting all adverse events to the Sponsor, investigators must comply with requirements for reporting serious adverse events to the local health authority and IRB/EC. Investigators may receive written IND safety reports or other safety-related communications from the Sponsor. Investigators are responsible for ensuring that such reports are reviewed and processed in accordance with health authority requirements and the policies and procedures established by their IRB/EC, and archived in the site's study file.

8.4 CONFIDENTIALITY

The Sponsor maintains confidentiality standards by coding each patient enrolled in the study through assignment of a unique patient identification number. This means that patient names are not included in datasets that are transmitted to any Sponsor location.

Patient medical information obtained by this study is confidential and may be disclosed to third parties only as permitted by the Informed Consent Form (or separate authorization for use and disclosure of personal health information) signed by the patient, unless permitted or required by law.

Medical information may be given to a patient's personal physician or other appropriate medical personnel responsible for the patient's welfare, for treatment purposes.

Given the complexity and exploratory nature of the analyses, data derived from exploratory biomarker specimens will generally not be provided to study investigators or patients unless required by law. The aggregate results of any conducted research will be available in accordance with the effective Sponsor policy on study data publication (see Section 9.5).

"Use of this document is governed by the terms of use on the first page of this document."

Data generated by this study must be available for inspection upon request by representatives of national and local health authorities; Sponsor monitors, representatives, and collaborators; and the IRB/EC for each study site, as appropriate.

8.5 FINANCIAL DISCLOSURE

Investigators will provide the Sponsor with sufficient, accurate financial information in accordance with local regulations to allow the Sponsor to submit complete and accurate financial certification or disclosure statements to the appropriate health authorities. Investigators are responsible for providing information on financial interests during the course of the study and for 1 year after completion of the study.

9. STUDY DOCUMENTATION, MONITORING, AND ADMINISTRATION

9.1 STUDY DOCUMENTATION

The investigator must maintain adequate and accurate records to enable the conduct of the study to be fully documented, including, but not limited to, the protocol, protocol amendments, Informed Consent Forms, and documentation of IRB/EC and governmental approval. In addition, at the end of the study, the investigator will receive the patient data, including an audit trail containing a complete record of all changes to data.

9.2 PROTOCOL DEVIATIONS

The investigator should document and explain any protocol deviations. The investigator should promptly report any deviations that might have an impact on patient safety and data integrity to the Sponsor and to the IRB/EC in accordance with established IRB/EC policies and procedures. The Sponsor will review all protocol deviations and assess whether any represent a serious breach of Good Clinical Practice guidelines and require reporting to health authorities. As per the Sponsor's standard operating procedures, prospective requests to deviate from the protocol, including requests to waive protocol eligibility criteria, are not allowed.

9.3 SITE INSPECTIONS

Site visits will be conducted by the Sponsor or an authorized representative for inspection of study data, subjects' medical records, and eCRFs. The investigator will permit national and local health authorities; Sponsor monitors, representatives, and collaborators; and the IRBs/ECs to inspect facilities and records relevant to this study.

9.4 ADMINISTRATIVE STRUCTURE

This trial will be sponsored by Genentech, a member of the Roche group, and will be managed by Genentech and CROs. CROs will provide clinical operations management, data management, biostatistics, and medical monitoring.

"Use of this document is governed by the terms of use on the first page of this document."

An IxRS will be used to assign patient numbers, monitor enrollment and patient status, and manage study treatment requests and study drug shipments.

A central laboratory will be used for the optional tumor assessment and collection of blood specimens.

Patient data will be recorded via an EDC system with the use of eCRFs (see Section 7.2).

9.5 PUBLICATION OF DATA AND PROTECTION OF TRADE SECRETS

Regardless of the outcome of a trial, the Sponsor is dedicated to openly providing information on the trial to health care professionals and to the public, both at scientific congresses and in peer-reviewed journals. The Sponsor will comply with all requirements for the publication of study results. For more information, refer to the Roche Global Policy on Sharing of Clinical Trials Data at the following Web site:

http://www.roche.com/roche_global_policy_on_sharing_of_clinical_study_information.pdf

The results of this study may be published or presented at scientific congresses. For all clinical trials in patients involving an IMP for which a marketing authorization application has been filed or approved in any country, the Sponsor aims to submit a journal manuscript reporting primary clinical trial results within 6 months after the availability of the respective Clinical Study Report. In addition, for all clinical trials in patients involving an IMP for which a marketing authorization application has been filed or approved in any country, the Sponsor aims to publish results from analyses of additional endpoints and exploratory data that are clinically meaningful and statistically sound.

The investigator must agree to submit all manuscripts or abstracts to the Sponsor prior to submission for publication or presentation. This allows the Sponsor to protect proprietary information and to provide comments based on information from other studies that may not yet be available to the investigator.

In accordance with standard editorial and ethical practice, the Sponsor will generally support publication of multicenter trials only in their entirety and not as individual center data. In this case, a coordinating investigator will be designated by mutual agreement.

Authorship will be determined by mutual agreement and in line with International Committee of Medical Journal Editors authorship requirements. Any formal publication of the study in which contribution of Sponsor personnel exceeded that of conventional monitoring will be considered as a joint publication by the investigator and the appropriate Sponsor personnel.

"Use of this document is governed by the terms of use on the first page of this document."

Any inventions and resulting patents, improvements, and/or know-how originating from the use of data from this study will become and remain the exclusive and unburdened property of the Sponsor, except where agreed otherwise.

9.6 PROTOCOL AMENDMENTS

Any protocol amendments will be prepared by the Sponsor. Protocol amendments will be submitted to the IRB/EC and to regulatory authorities in accordance with local regulatory requirements.

Approval must be obtained from the IRB/EC and regulatory authorities (as locally required) before implementation of any changes, except for changes necessary to eliminate an immediate hazard to patients or changes that involve logistical or administrative aspects only (e.g., change in Medical Monitor or contact information).

"Use of this document is governed by the terms of use on the first page of this document."

10. REFERENCES

Bai S, Jorga K, Xin Y, et al. A guide to rational dosing of monoclonal antibodies. Clin Pharmacokinet 2012;51(2):119–35.

Barlesi F, Park K, Ciardiello F, et al. Primary analysis from OAK, a randomized phase III study comparing atezolizumab with docetaxel in advanced NSCLC. Oral presentation at European Society for Medical Oncology (ESMO) 2016. Abstract LBA44_PR.

Besse B, Johnson ML, Jänne PA, et al. Phase II, single-arm trial (BIRCH) of atezolizumab as first-line or subsequent therapy for locally advanced or metastatic PD-L1–selected non-small cell lung cancer (NSCLC). The European Cancer Congress 2015, Vienna, Austria. Abstract 16LBA.

Borghaei H, Paz-Ares L, Horn L, et al. Nivolumab versus docetaxel in advanced nonsquamous non–small-cell lung cancer. New Engl J Med 2015;373(17):1627–39.

Brahmer J, Reckamp KL, Baas P, et al. Nivolumab versus docetaxel in advanced squamous-cell non–small-cell lung cancer. New Engl J Med. 2015;373(2):123–35.

Brookmeyer R, Crowley J. A confidence interval for the median survival time. Biometrics 1982;38:29–41.

Cetin K, Ettinger DS, Hei YJ, et al. Survival by histologic subtype in stage IV nonsmall cell lung cancer based on data from the Surveillance, Epidemiology and End Results Program. Clin Epidemiol 2011;3:139–48.

Chen DS, Irving BA, Hodi FS. Molecular pathways: next-generation immunotherapy—inhibiting programmed death-ligand 1 and programmed death-1. Clin Cancer Research 2012;18(24):6580–7.

Chiou VL, Burotto M. Pseudoprogression and Immune-Related Response in Solid Tumors. J Clin Oncol 2015;33(31):3541–3.

Fehrenbacher L, Spira A, Ballinger M, et al, for the POPLAR Study Group. Atezolizumab versus docetaxel for patients with previously treated non-small-cell lung cancer (POPLAR): a multicentre, open-label, phase 2 randomised controlled trial. Lancet 2016; published online March 9. http://dx.doi.org/10.1016/S0140-6736(16)00587-0.

Gettinger SN, Horn L, Gandhi L, et al. Overall survival and long-term safety of nivolumab (anti–programmed death 1 antibody, BMS-936558, ONO-4538) in patients with previously treated advanced non–small-cell lung cancer. J Clin Oncol 2015;33(18):2004–12.

Goldstraw P, Chansky K, Crowley J, et al. The IASLC Lung Cancer Staging Project: Proposals for Revision of the TNM Stage Groupings in the Fourthcoming (Eighth) Edition of the TNM Classification for Lung Cancer. J Thorac Onc 2015;11(1):39–51.

Hales RK, Banchereau J, Ribas A, et al. Assessing oncologic benefit in clinical trials of immunotherapy agents. Ann Oncol 2010:mdq048.

"Use of this document is governed by the terms of use on the first page of this document."

Herbst RS, Baas P, Kim DW, et al. Pembrolizumab versus docetaxel for previously treated, PD-L1-positive, advanced non-small-cell lung cancer (KEYNOTE-010): a randomised controlled trial. Lancet 2016;387(10027):1540–50.

Hodi FS, O'Day SJ, McDermott DF, et al. Improved survival with ipilimumab in patients with metastatic melanoma. New Engl J Med 2010;363(8):711–23.

Howlader N, Noone AM, Krapcho M, et al. ER Cancer Statistics Review, 1975-2012, National Cancer Institute. Bethesda, MD, http://seer.cancer.gov/csr/1975_2012/, based on November 2013 SEER data submission, posted to the SEER web site, April 2014.

Kantoff PW, Higano CS, Shore ND. Sipuleucel-T immunotherapy for castration-resistant prostate cancer. New Engl J Med 2010;363(5):411–22.

Langer CJ, Besse B, Gualberto A, et al. The evolving role of histology in the management of advanced non–small-cell lung cancer. J Clin Oncol 2010;28(36):5311–20.

Lawrence MS, Stojanov P, Polak P, et al. Mutational heterogeneity in cancer and the search for new cancer-associated genes. Nature 2013;499(7457):214–8.

Molina JR, Yang P, Cassivi SD et al. 2008: Non–small cell lung cancer: epidemiology, risk factors, treatment, and survivorship. Mayo Clin Proc 2008;584–94.

Miller KD, Siegel RL, Lin CC, et al. Cancer treatment and survivorship statistics, 2016. CA Cancer J Clin 2016;66:271–89.

National Comprehensive Cancer Network (NCCN). NCCN Guidelines® for Non-Small Cell Lung Cancer. [resource on the Internet]. November 2016. Available at: https://www.nccn.org/professionals/physician_gls/f_guidelines.asp.

National Comprehensive Cancer Network (NCCN). NCCN Guidelines® for Non-Small Cell Lung Cancer (footnote hh). [resource on the Internet]. September 2017. https://www.nccn.org/professionals/physician_gls/f_guidelines.asp.

Reck M, Rodríguez-Abreu D, Robinson AG. Pembrolizumab versus chemotherapy for PD-L1–positive non–small-cell lung cancer. New Engl J Med 2016;375(19):1823-33.

Riely GJ, Politi KA, Miller VA, et al. Update on epidermal growth factor receptor mutations in non-small cell lung cancer. Clin Cancer Res 2006;12(24):7232 – 41.

Rittmeyer A, Barlesi F, Waterkamp D, et al. Atezolizumab versus docetaxel in patients with previously treated non-small-cell lung cancer (OAK): a phase 3, open-label, multicentre randomised controlled trial. Lancet 2017;389(10066):255–65.

Rizvi NA, Hellmann MD, Snyder A. Mutational landscape determines sensitivity to PD-1 blockade in non–small cell lung cancer. Science 2015;348(6230):124–8.

"Use of this document is governed by the terms of use on the first page of this document."

Scagliotti GV, Parikh P, von Pawel J, et al. Phase III study comparing cisplatin plus gemcitabine with cisplatin plus pemetrexed in chemotherapy-naive patients with advanced-stage non-small-cell lung cancer. J Clin Oncol 2008; 26: 3543–51.

Sandler A, Gray R, Perry MC. Paclitaxel–carboplatin alone or with bevacizumab for non–small-cell lung cancer. New Engl J Med 2006;355(24):2542–50.

Schiller JH, Harrington D, Belani CP, et al. Eastern Cooperative Oncology Group. Comparison of four chemotherapy regimens for advanced non–small-cell lung cancer. N Engl J Med 2002;346:92–8.

SEER Stat Fact Sheets: Lung and Bronchus Cancer. National Cancer Institute website [updated April 2016; accessed October 7, 20116.] Available from http://seer.cancer.gov/statfacts/html/lungb.html.

Shtivelman E, Hensing T, Simon GR, et al. Molecular pathways and therapeutic targets in lung cancer. Oncotarget 2014;5(6):1392–433.

Socinski M, Creelan B, Horn, L et al. A phase 3 trial of Nivolumab vs Investigator's Choice (IC) of Platinum-Based Doublet Chemotherapy as First-Line Therapy for Stage IV/Recurrent Programmed Death Ligand 1 (PD-L1) – Positive NSCLC. Oral presentation at European Society for Medical Oncology (ESMO) 2016. Abstract LBA7.

Thomas A, Liu SV, Subramaniam DS, et al. Refining the treatment of NSCLC according to histological and molecular subtypes. Nat Rev Clin Oncol 2015;12(9):511–26.

Topalian SL, Hodi FS, Brahmer JR, et al. Safety, activity, and immune correlates of anti–PD-1 antibody in cancer. N Engl J Med 2012;366:2443–54.

Travis WD, Brambilla E, Noguchi M, et al. International association for the study of lung cancer/american thoracic society/european respiratory society international multidisciplinary classification of lung adenocarcinoma. J Thorac Oncol. 2011 Feb;6(2):244–85.

Tsui DW, Berger MF. Profiling Non-Small Cell Lung Cancer: From Tumor to Blood. Clin Cancer Res 2016 Feb 15;22(4):790–2.

Vansteenkiste J, Fehrenbacher L, Spira AI, et al. Atezolizumab monotherapy vs docetaxel in 2L/3L non-small cell lung cancer: Primary analyses for efficacy, safety and predictive biomarkers from a randomized phase II study (POPLAR). Presented at: 2015 European Cancer Congress; September 25-29; Vienna, Austria. Abstract 14LBA.

"Use of this document is governed by the terms of use on the first page of this document."

Appendix 1*a*
Schedule of Activities

Activity	Screening [a]	All Treatment Cycles [b]		Treatment DC [c]	On-Study Follow-Up	Study Completion/ DC [d]	Optional Long-Term Follow-Up [e]
	Days –28 to –1	Day 1, Cycle 1	Day 1, Cycle 2 and Beyond Every 21 (±3) Days	≤30 Days after Last Dose of Study Treatment	Every 3 Months until Study Completion/DC	≤30 Days after Study Completion/DC	
Informed consent	x [f]						
Review of eligibility criteria	x						
Demographics	x						
Medical history and baseline conditions [g]	x						
Vital signs [h]	x	x	x				
Weight	x						
Height	x						
Complete physical examination [i]	x						
Limited physical examination [j]		x	x				
ECOG performance status	x	x	x	x		x	
Concomitant medications [k]	x	x	x	x			
Hematology [l]	x	x	x	x			
Serum chemistry [m]	x	x	x	x			
Coagulation test (aPTT or INR)	x						
Pregnancy test	x [n]	Every 3 cycles [n]					
TSH, free T3/T4 [o]	x	Every 3 cycles					

"Use of this document is governed by the terms of use on the first page of this document."

Appendix 1*a*
Schedule of Activities

Activity	Screening [a]	All Treatment Cycles [b]		Treatment DC [c]	On-Study Follow-Up	Study Completion/ DC [d]	Optional Long-Term Follow-Up [e]
	Days –28 to –1	Day 1, Cycle 1	Day 1, Cycle 2 and Beyond Every 21 (±3) Days	≤30 Days after Last Dose of Study Treatment	Every 3 Months until Study Completion/DC	≤30 Days after Study Completion/DC	
Urinalysis [p]	x						
HIV, HBV, HCV serology [q]	x						
EGFR/ALK testing	x [r]						
Whole-blood samples for biomarker analyses [s] (mandatory)	x	Day 1 of Cycles 2 and 4, and at disease progression or loss of clinical benefit					
Whole-blood sample for RBR [s] & exploratory biomarker analyses (optional) [s, t]			Day 1, Cycle 3 only				
Tissue biopsies [s] (optional)	x [u]	x [u]		x [u]			
Study drug administration [v]		x	x				
Tumor response assessment [w]	x [w]	Every 6 weeks (±7 days) during treatment regardless of dose delays, for the first 12 months following Cycle 1, Day 1. After 12 months, tumor assessment will be required every 9 weeks (±7 days). [w]					
Adverse events [x]	x	x	x	x	x	x	
Survival and anti-cancer therapy follow-up [y]					x		
Long-term survival follow-up (optional)[e]							x

"Use of this document is governed by the terms of use on the first page of this document."

Appendix 1*a*
Schedule of Activities

ALK = anaplastic lymphoma kinase; aPTT = activated partial thromboplastin time; CT = computed tomography; ECOG = Eastern Cooperative Oncology Group; eCRF = electronic Case Report Form; EGFR = epidermal growth factor receptor; HBcAb = hepatitis B core antibody; HBsAg = hepatitis B surface antigen; HBV = hepatitis B virus; HCV = hepatitis C virus; INR = international normalized ratio; MRI = magnetic resonance image; NSCLC = non–small cell lung cancer; RBR = Research Biomarker Repository; TH = thyroid hormone; TSH = thyroid stimulating hormone.

Notes: Dosing and all assessments coinciding with Day 1 of each cycle should be performed within ± 3 days of the specified visit date, unless otherwise noted. Physical examinations, safety laboratory assessments, and assessment of ECOG performance status may be done within 96 hours prior to dosing.

[a] Results of standard-of-care tests or examinations performed prior to obtaining informed consent and within 28 days prior to study entry (except where otherwise specified) may be used for screening assessments rather than repeating such tests.

[b] All assessments should be performed on the day of the specified visit unless a time window for that assessment is otherwise specified. Assessments scheduled on the day of study drug administration (Day 1) of each cycle should be performed prior to study drug infusion unless otherwise noted. If scheduled dosing and study assessments are precluded because of a holiday, weekend, or other event, then dosing may be postponed to the soonest following date, with subsequent dosing continuing on a 21-day schedule. If treatment was postponed for fewer than 3 days, the patient can resume the original schedule.

[c] Patients will be asked to return to the clinic no more than 30 days after the last dose for a treatment discontinuation visit. Note that the Study Discontinuation eCRF should not be completed until after the patient has discontinued from the study.

[d] A patient is considered to have completed the study if he or she does not discontinue from the study prior to the end of the study. The end of this study is defined as the date after which all enrolled patients who remain alive have been followed for at least 18 months from the start of study drug. Thereafter, patients will be followed for long-term survival. Patients who complete the study will return to the clinic for a study completion visit within 30 days following study completion. Patients who discontinue the study prematurely will return to the clinic for a study discontinuation visit within 30 days following discontinuation from the study.

[e] For patients who provide consent for optional long-term follow-up, *refer to Appendix 1b.for the schedule of assessments*.

[f] Informed consent must be documented before any study-specific screening procedure is performed, and may be obtained no more than 28 days before initiation of study drug.

[g] Includes clinically significant diseases, surgeries, cancer history (including stage and date of diagnosis, prior cancer therapies, and procedures), reproductive status, and smoking history.

[h] Includes heart rate, respiratory rate, and systolic and diastolic blood pressures while the patient is in a seated position, and temperature. For specific requirements during atezolizumab infusions, refer to Section 4.3.2.

"Use of this document is governed by the terms of use on the first page of this document."

Appendix 1*a*
Schedule of Activities

[i] A complete physical examination includes evaluation of the head, eyes, ears, nose, and throat, and the cardiovascular, dermatological, musculoskeletal, respiratory, gastrointestinal, and neurological systems. Record abnormalities observed at baseline on the General Medical History and Baseline Conditions eCRF. At subsequent visits, record new or worsened clinically significant abnormalities on the Adverse Events eCRF.

[j] Perform a limited, symptom-directed physical examination within 96 hours prior to dosing at specified timepoints, and as clinically indicated. Changes from baseline abnormalities should be recorded in patient notes. New or worsened clinically significant abnormalities should be recorded as adverse events on the Adverse Event eCRF. (See Section 4.5.3)

[k] Includes any medication (e.g., prescription drugs, vaccines, over-the-counter drugs, herbal or homeopathic remedies, nutritional supplements) used by the patient from 7 days prior to screening until the study completion/discontinuation visit. At each post-screening visit, changes to current medications or new medications used since the last documentation of medications will be recorded.

[l] Hematology consists of CBC, including RBC count, hemoglobin, hematocrit, WBC count with differential (neutrophils, lymphocytes, eosinophils, monocytes, basophils, and other cells), and platelet count.

[m] Serum chemistry includes glucose, BUN or urea, creatinine, sodium, potassium, magnesium, chloride, bicarbonate or total CO_2, calcium, phosphorus, total and direct bilirubin, ALT, AST, alkaline phosphatase, LDH, total protein, and albumin. Serum will be collected every 3 cycles to test thyroid function. HBV and HCV will be tested as clinically indicated. CRP from screening and Cycle 3, Day 1 will be tested centrally from previously obtained samples; no additional samples will be needed.

[n] All women of childbearing potential (including those who have had a tubal ligation) must have a negative serum pregnancy test at screening (within 14 days prior to Cycle 1, Day 1). Perform urine or serum pregnancy test every three cycles thereafter. Confirm any positive urine pregnancy test with a serum pregnancy test. Patients with positive serum pregnancy tests should be discontinued from study drug.

[o] Thyroid function testing (TH, free T3, free T4) will be performed at screening, Cycle 1, Day 1, and every three cycles thereafter.

[p] Urinalysis by dipstick (specific gravity, pH, glucose, protein, ketones, and blood).

[q] At screening, patients will be tested for HIV, HBsAg, total HBcAb, and HCV antibody. If a patient has a negative HBsAg test and a positive total HBcAb test at screening, an HBV DNA test should be performed. If a patient has a positive HCV antibody test at screening, an HCV RNA test must be performed to determine if the patient has an active HCV infection.

[r] EGFR and ALK testing with approved test to be conducted at or prior to screening to rule out presence of EGFR-sensitizing mutations or ALK rearrangements. If either test is confirmed positive, the patient will be ineligible for enrollment (see Section 4.1.2 for exclusion criteria and Section *1.1.3* for NCCN guidelines on testing). For patients who are unable or unwilling to provide a tissue sample to determine EGFR status, the EGFR Cobas® plasma test is available. Please contact the Medical Monitor for additional options.

[s] See Appendix 2 for detailed schedule.

"Use of this document is governed by the terms of use on the first page of this document."

Appendix 1*a*
Schedule of Activities

[t]	The optional RBR whole blood sample requires separate specific informed consent and the sample can be collected at any time during the course of the study.
[u]	Optional tissue biopsy specimen(s) may be submitted at any time prior to or during enrollment at the following timepoints: from the most recent diagnosis of metastatic NSCLC, during treatment, and/or at progression. The quality of the sample should be confirmed by a local pathologist and conform to the Foundation Medicine Pathology Review Process for Optimal Tissue Utilization.
[v]	The initial dose will be delivered over 60 (± 15) minutes. If the first infusion is well tolerated, all subsequent infusions may be delivered over 30 (± 10) minutes. Atezolizumab treatment may be continued until lack of clinical benefit, unacceptable toxicity, withdrawal of consent, patient or physician decision to discontinue treatment, or death.
[w]	CT scans (with oral/intravenous contrast unless contraindicated) or MRI scans of the chest and abdomen. A CT scan of the pelvis is required at screening and as clinically indicated or as per local standard of care at subsequent response evaluations. A CT (with contrast) or MRI scan of the head must be done at screening to evaluate CNS metastasis in all patients. See Section 4.5.5 for details. Perform tumor assessment at baseline and every 6 weeks (± 7 days) thereafter regardless of dose delays for the first 12 months following Cycle 1, Day 1. After 12 months, tumor assessment will be required every 9 weeks (± 7 days); tumor assessments will continue until disease progression per RECIST v1.1 or loss of clinical benefit, consent withdrawal, study discontinuation, study completion or death, whichever occurs first. Patients who discontinue treatment for reasons other than disease progression (e.g., toxicity) will continue scheduled tumor assessments until disease progression, withdrawal of consent, study discontinuation, study completion (i.e., end of study), or death, whichever occurs first. In the absence of disease progression, tumor assessments should continue regardless of whether patients start a new anti-cancer therapy, unless consent is withdrawn.
[x]	All serious adverse events and adverse events of special interest, regardless of relationship to study drug, will be reported until 90 days after the last dose of study drug or initiation of new anti-cancer therapy, whichever occurs first. All other adverse events, regardless of relationship to study drug, will be reported until 30 days after the last dose of study drug or initiation of new anti-cancer therapy, whichever occurs first. After this period, the investigator should report any serious adverse events or adverse events of special interest.
[y]	Survival follow-up should continue until at least 18 months following the start of the study for each patient and at end of study defined as 18 months after the last patient's C1D1, death, withdrawal of consent, the patient is lost to follow-up, or whichever occurs first.

"Use of this document is governed by the terms of use on the first page of this document."

Appendix 1b
Optional Long-Term Follow-Up Schedule of Assessments

Activity	*All Treatment Cycles*	*Treatment Completion/DC* [b,c]	*Safety Follow- Up (Phone Call)*	*Overall Survival Follow-Up (Phone Call)*	*Study Completion/ DC*
	Continued dosing Every 21 (±3) Days [a]		*30 days post-last treatment*	*Every 3 months for the first year, then every 6 months thereafter, or up to 5 years from enrollment of the first patient into the study until the last patient dies, withdraws consent, is lost to follow-up, whichever occurs first*	
Study drug administration	*x*				
Adverse Event	*x*	*x*	*x*		
Survival Information				*x*	*x*

DC=discontinuation

[a] *Patients on study drug will continue therapy until they have received a total of 2 years of atezolizumab treatment (32 cycles).*

[b] *Patients will be asked to return to the clinic no more than 30 days after the last dose for a treatment discontinuation visit. Note that the Study Discontinuation eCRF should not be completed until after the patient has discontinued from the study.*

[c] *Patients who complete the optional long-term follow-up study will return to the clinic for a study completion visit within 30 days following study completion. Patients who discontinue the study prematurely will return to the clinic for a study discontinuation visit within 30 days following discontinuation from the study.*

"Use of this document is governed by the terms of use on the first page of this document."

Appendix 2
Schedule of Biomarker Samples

Visit	Timepoint	Sample Type	Proposed Biomarkers	Mandatory/ Optional
Screening (Day −28 to Day −1)	NA	Plasma	bTMB	Mandatory
Baseline (Day 1 of Cycle 1)	Prior to infusion	Plasma	bTMB	Mandatory
Baseline (Day 1 of Cycle 1)	Prior to infusion	PBMC	Immune gene expression	Mandatory
Day 1 of Cycle 2	Prior to infusion	Plasma	bTMB	Mandatory
Day 1 of Cycle 2	Prior to infusion	PBMC	Immune gene expression	Mandatory
Day 1 of Cycle 3	Prior to infusion	Plasma	bTMB	Optional
Day 1 of Cycle 4	Prior to infusion	Plasma	bTMB	Mandatory
During treatment	At radiographic progression or loss of clinical benefit	Plasma	bTMB	Mandatory
During treatment	At radiographic progression or loss of clinical benefit	PBMC	Immune gene expression	Mandatory
NA	Anytime during study	Tissue	tTMB and somatic alterations	Optional

bTMB = blood tumor mutational burden; NA = not applicable; PBMC = peripheral blood mononuclear cells; tTMB = tissue tumor mutational burden.

"Use of this document is governed by the terms of use on the first page of this document."

Appendix 3
IASLC Lung Cancer Staging Project Proposed for the Eighth Edition of the American Joint Committee on Cancer Non–Small Cell Lung Cancer Staging System[1]

T: Primary tumor	
Tx	Primary tumor cannot be assessed or tumor proven by presence of malignant cells in sputum or bronchial washings but not visualized by imaging or bronchoscopy
T0	No evidence of primary tumor
Tis	Carcinoma in situ
T1	Tumor ≤3 cm in greatest dimension surrounded by lung or visceral pleura without bronchoscopic evidence of invasion more proximal than the lobar bronchus (i.e., not in the main bronchus)[a]
**T1a(mi)**	**Minimally invasive adenocarcinoma**[b]
**T1a**	**Tumor ≤1 cm in greatest dimension**[a]
**T1b**	**Tumor >1 cm but ≤2 cm in greatest dimension**[a]
**T1c**	**Tumor >2 cm but ≤3 cm in greatest dimension**[a]
T2	Tumor >3 cm **but ≤5 cm** or tumor with any of the following features[c]:<br>- **Involves main bronchus regardless of distance from the carina but without involvement of the carina**<br>- Invades visceral pleura<br>- **Associated with atelectasis or obstructive pneumonitis that extends to the hilar region, involving part or all of the lung**
**T2a**	**Tumor >3 cm but ≤4 cm in greatest dimension**
**T2b**	**Tumor >4 cm but ≤5 cm in greatest dimension**
T3	**Tumor >5 cm but ≤7 cm in greatest dimension** or associated with separate tumor nodule(s) in the same lobe as the primary tumor or directly invades any of the following structures: chest wall (including the parietal pleura and superior sulcus tumors), phrenic nerve, parietal pericardium
T4	**Tumor >7 cm in greatest dimension** or associated with separate tumor nodule(s) in a different ipsilateral lobe than that of the primary tumor or invades any of the following structures: **diaphragm**, mediastinum, heart, great vessels, trachea, recurrent laryngeal nerve, esophagus, vertebral body, and carina
N: Regional lymph node involvement	
Nx	Regional lymph nodes cannot be assessed
N0	No regional lymph node metastasis
N1	Metastasis in ipsilateral peribronchial and/or ipsilateral hilar lymph nodes and intrapulmonary nodes, including involvement by direct extension
N2	Metastasis in ipsilateral mediastinal and/or subcarinal lymph node(s)
N3	Metastasis in contralateral mediastinal, contralateral hilar, ipsilateral or contralateral scalene, or supraclavicular lymph node(s)
M: Distant metastasis	
M0	No distant metastasis
M1	Distant metastasis present
M1a	Separate tumor nodule(s) in a contralateral lobe; tumor with pleural or pericardial nodule(s) or malignant pleural or pericardial effusion[d]
**M1b**	**Single extrathoracic metastasis**[e]
**M1c**	**Multiple extrathoracic metastases in one or more organs**

Note: Changes to the seventh edition are in bold.

[a]The uncommon superficial spreading tumor of any size with its invasive component limited to the bronchial wall, which may extend proximal to the main bronchus, is also classified as T1a.

[b]Solitary adenocarcinoma, ≤ 3cm with a predominately lepidic pattern and ≤ 5mm invasion in any one focus.

[c]T2 tumors with these features are classified as T2a if ≤4 cm in greatest dimension or if size cannot be determined, and T2b if >4 cm but ≤5 cm in greatest dimension.

[d]Most pleural (pericardial) effusions with lung cancer are due to tumor. In a few patients, however, multiple microscopic examinations of pleural (pericardial) fluid are negative for tumor and the fluid is nonbloody and not an exudate. When these elements and clinical judgment dictate that the effusion is not related to the tumor, the effusion should be excluded as a staging descriptor.

[e]This includes involvement of a single distant (nonregional) lymph node.

"Use of this document is governed by the terms of use on the first page of this document."

Appendix 3
IASLC Lung Cancer Staging Project Proposed for the Eighth Edition of the American Joint Committee on Cancer Non–Small Cell Lung Cancer Staging System[1] (cont.)

		N categories			
		Overall stage			
Descriptor in 7th edition	Proposed T/M	N0	N1	N2	N3
T1 ≤ 1 cm	T1a	**IA1** (IA)	**IIB** (IIA)	IIIA	IIIB
T1 > 1-2 cm	T1b	**IA2** (IA)	**IIB** (IIA)	IIIA	IIIB
T1 > 2-3 cm	T1c	**IA3** (IA)	**IIB** (IIA)	IIIA	IIIB
T2 > 3-4 cm	T2a	IB	**IIB** (IIA)	IIIA	IIIB
T2 > 4-5 cm	T2b	**IIA** (IB)	**IIB** (IIA)	IIIA	IIIB
T2 > 5-7 cm	T3	**IIB** (IIA)	**IIIA** (IIB)	**IIIB** (IIIA)	**IIIC** (IIIB)
T3 structures	T3	IIB	IIIA	**IIIB** (IIIA)	**IIIC** (IIIB)
T3 > 7 cm	T4	**IIIA** (IIB)	IIIA	**IIIB** (IIIA)	**IIIC** (IIIB)
T3 diaphragm	T4	**IIIA** (IIB)	IIIA	**IIIB** (IIIA)	**IIIC** (IIIB)
T3 endobronchial: location/atelectasis 3-4 cm	T2a	**IB** (IIB)	**IIB** (IIIA)	IIIA	IIIB
T3 endobronchial: location/atelectasis 4-5 cm	T2b	**IIA** (IIB)	**IIB** (IIIA)	IIIA	IIIB
T4	T4	IIIA	IIIA	IIIB	**IIIC** (IIIB)
M1a	M1a	**IVA** (IV)	**IVA** (IV)	**IVA** (IV)	**IVA** (IV)
M1b single lesion	M1b	**IVA** (IV)	**IVA** (IV)	**IVA** (IV)	**IVA** (IV)
M1c multiple lesions	M1c	**IVB** (IV)	**IVB** (IV)	**IVB** (IV)	**IVB** (IV)

[a]Where there is a change, the resultant stage groupings proposed for the eighth edition are in bold, and the stage in the seventh edition is given in parenthesis.
T, tumor; M, metastasis.

[1] Goldstraw P, Chansky K, Crowley J, et al. The IASLC Lung Cancer Staging Project: Proposals for Revision of the TNM Stage Groupings in the Fourthcoming (Eighth) Edition of the TNM Classification for Lung Cancer. J Thorac Onc 2015;11(1): 39-51.

"Use of this document is governed by the terms of use on the first page of this document."

Appendix 4
Response Evaluation Criteria in Solid Tumors (RECIST): Excerpt from Original Publication

Selected sections from the Response Evaluation Criteria in Solid Tumors (RECIST), Version 1.1[1] are presented below, with slight modifications and the addition of explanatory text as needed for clarity.[2]

MEASURABILITY OF TUMOR AT BASELINE

DEFINITIONS

At baseline, tumor lesions/lymph nodes will be categorized measurable or nonmeasurable as follows:

Measurable Tumor Lesions

Tumor Lesions. Tumor lesions must be accurately measured in at least one dimension (longest diameter in the plane of measurement is to be recorded) with a minimum size of:

- 10 mm by computed tomography (CT) or magnetic resonance imaging (MRI) scan (CT/MRI scan slice thickness/interval no greater than 5 mm)
- 10-mm caliper measurement by clinical examination (lesions that cannot be accurately measured with calipers should be recorded as nonmeasurable)
- 20 mm by chest X-ray

Malignant Lymph Nodes. To be considered pathologically enlarged and measurable, a lymph node must be $\geq$ 15 mm in the short axis when assessed by CT scan (CT scan slice thickness recommended to be no greater than 5 mm). At baseline and in follow-up, only the short axis will be measured and followed. See also notes below on “Baseline Documentation of Target and Nontarget Lesions” for information on lymph node measurement.

Nonmeasurable Tumor Lesions

Nonmeasurable tumor lesions encompass small lesions (longest diameter $<$ 10 mm or pathological lymph nodes with $\geq$ 10 to $<$ 15 mm short axis), as well as truly nonmeasurable lesions. Lesions considered truly nonmeasurable include leptomeningeal disease, ascites, pleural or pericardial effusion, inflammatory breast disease, lymphangitic involvement of skin or lung, peritoneal spread, and abdominal masses/abdominal organomegaly identified by physical examination that is not measurable by reproducible imaging techniques.

---

1 Eisenhauer EA, Therasse P, Bogaerts J, et al. New response evaluation criteria in solid tumors: Revised RECIST guideline (Version 1.1). Eur J Cancer 2009;45:228–47.

2 For consistency within this document, the section numbers and cross-references to other sections within the article have been deleted and minor formatting changes have been made.

"Use of this document is governed by the terms of use on the first page of this document."

Special Considerations Regarding Lesion Measurability

Bone lesions, cystic lesions, and lesions previously treated with local therapy require particular comment, as outlined below.

Bone lesions:

- Bone scan, positron emission tomography (PET) scan, or plain films are not considered adequate imaging techniques to measure bone lesions. However, these techniques can be used to confirm the presence or disappearance of bone lesions. When a bone scan is the sole indicator of progression, progression in bone will be defined as when at least two or more new lesions are seen on bone scan compared with screening. In situations where the scan findings are suggestive of a flare reaction, or apparent new lesion(s) which may represent trauma, these results must be confirmed with other imaging modalities such as MRI or fine-cut CT to constitute progression. Only a single new bone lesion on bone scan is required for progression if the lesion can be correlated on CT, MRI or plain film.
- Lytic bone lesions or mixed lytic-blastic lesions, with identifiable soft tissue components, that can be evaluated by cross-sectional imaging techniques such as CT or MRI can be considered measurable lesions if the soft tissue component meets the definition of measurability described above.
- Blastic bone lesions are nonmeasurable.

Cystic lesions:

- Lesions that meet the criteria for radiographically defined simple cysts should not be considered malignant lesions (neither measurable nor nonmeasurable) since they are, by definition, simple cysts.
- Cystic lesions thought to represent cystic metastases can be considered measurable lesions if they meet the definition of measurability described above. However, if noncystic lesions are present in the same patient, these are preferred for selection as target lesions.

Lesions with prior local treatment:

- Tumor lesions situated in a previously irradiated area, or in an area subjected to other loco-regional therapy, are usually not considered measurable unless there has been demonstrated progression in the lesion. Study protocols should detail the conditions under which such lesions would be considered measurable.

"Use of this document is governed by the terms of use on the first page of this document."

TARGET LESIONS: SPECIFICATIONS BY METHODS OF MEASUREMENTS

Measurement of Lesions

All measurements should be recorded in metric notation, with use of calipers if clinically assessed. All baseline evaluations should be performed as close as possible to the treatment start and never more than 4 weeks before the beginning of the treatment.

Method of Assessment

The same method of assessment and the same technique should be used to characterize each identified and reported lesion at baseline and during study. Imaging-based evaluation should always be the preferred option.

Clinical Lesions. Clinical lesions will only be considered measurable when they are superficial and ≥ 10 mm in diameter as assessed using calipers (e.g., skin nodules). For the case of skin lesions, documentation by color photography, including a ruler to estimate the size of the lesion, is suggested.

Chest X-Ray. Chest CT is preferred over chest X-ray, particularly when progression is an important endpoint, since CT is more sensitive than X-ray, particularly in identifying new lesions. However, lesions on chest X-ray may be considered measurable if they are clearly defined and surrounded by aerated lung.

CT, MRI. CT is the best currently available and reproducible method to measure lesions selected for response assessment. This guideline has defined measurability of lesions on CT scan based on the assumption that CT slice thickness is 5 mm or less. When CT scans have slice thickness greater than 5 mm, the minimum size for a measurable lesion should be twice the slice thickness. MRI is also acceptable.

If prior to enrollment it is known that a patient is unable to undergo CT scans with intravenous (IV) contrast because of allergy or renal insufficiency, the decision as to whether a noncontrast CT or MRI (without IV contrast) will be used to evaluate the patient at baseline and during the study should be guided by the tumor type under investigation and the anatomic location of the disease. For patients who develop contraindications to contrast after baseline contrast CT is done, the decision as to whether noncontrast CT or MRI (enhanced or nonenhanced) will be performed should also be based on the tumor type and the anatomic location of the disease and should be optimized to allow for comparison with the prior studies if possible. Each case should be discussed with the radiologist to determine if substitution of these other approaches is possible and, if not, the patient should be considered not evaluable from that point forward. Care must be taken in measurement of target lesions on a different modality

"Use of this document is governed by the terms of use on the first page of this document."

and interpretation of nontarget disease or new lesions since the same lesion may appear to have a different size with use of a new modality.

Ultrasound. Ultrasound is not useful in the assessment of lesion size and should not be used as a method of measurement.

Endoscopy, Laparoscopy, Tumor Markers, Cytology, Histology. The utilization of these techniques for objective tumor evaluation cannot generally be advised.

TUMOR RESPONSE EVALUATION

ASSESSMENT OF OVERALL TUMOR BURDEN AND MEASURABLE DISEASE

To assess objective response or future progression, it is necessary to estimate the overall tumor burden at baseline and to use this as a comparator for subsequent measurements. Measurable disease is defined by the presence of at least one measurable lesion, as detailed above.

BASELINE DOCUMENTATION OF TARGET AND NONTARGET LESIONS

When more than one measurable lesion is present at baseline, all lesions up to a maximum of five lesions total (and a maximum of two lesions per organ) representative of all involved organs should be identified as target lesions and will be recorded and measured at baseline. This means in instances where patients have only one or two organ sites involved, a maximum of two lesions (one site) and four lesions (two sites), respectively, will be recorded. Other lesions (albeit measurable) in those organs will be recorded as nonmeasurable lesions (even if the size is $>$10 mm by CT scan).

Target lesions should be selected on the basis of their size (lesions with the longest diameter) and be representative of all involved organs, but additionally, should lend themselves to reproducible repeated measurements. It may be the case that, on occasion, the largest lesion does not lend itself to reproducible measurement, in which circumstance the next largest lesion that can be measured reproducibly should be selected.

Lymph nodes merit special mention since they are normal anatomical structures that may be visible by imaging even if not involved by tumor. As noted above, pathological nodes that are defined as measurable and may be identified as target lesions must meet the criterion of a short axis of $\geq$15 mm by CT scan. Only the short axis of these nodes will contribute to the baseline sum. The short axis of the node is the diameter normally used by radiologists to judge if a node is involved by solid tumor. Nodal size is normally reported as two dimensions in the plane in which the image is obtained (for CT scan, this

"Use of this document is governed by the terms of use on the first page of this document."

is almost always the axial plane; for MRI the plane of acquisition may be axial, sagittal, or coronal). The smaller of these measures is the short axis. For example, an abdominal node that is reported as being 20 mm × 30 mm has a short axis of 20 mm and qualifies as a malignant, measurable node. In this example, 20 mm should be recorded as the node measurement. All other pathological nodes (those with short axis ≥ 10 mm but < 15 mm) should be considered nontarget lesions. Nodes that have a short axis < 10 mm are considered nonpathological and should not be recorded or followed.

A sum of the diameters (longest for non-nodal lesions, short axis for nodal lesions) for all target lesions will be calculated and reported as the baseline sum of diameters. If lymph nodes are to be included in the sum, then, as noted above, only the short axis is added into the sum. The baseline sum of diameters will be used as a reference to further characterize any objective tumor regression in the measurable dimension of the disease.

All other lesions (or sites of disease), including pathological lymph nodes, should be identified as nontarget lesions and should also be recorded at baseline. Measurements are not required and these lesions should be followed as "present," "absent," or in rare cases "unequivocal progression."

In addition, it is possible to record multiple nontarget lesions involving the same organ as a single item on the Case Report Form (CRF) (e.g., "multiple enlarged pelvic lymph nodes" or "multiple liver metastases").

RESPONSE CRITERIA

Evaluation of Target Lesions

This section provides the definitions of the criteria used to determine objective tumor response for target lesions.

Complete response (CR): disappearance of all target lesions

- Any pathological lymph nodes (whether target or nontarget) must have reduction in short axis to < 10 mm.

Partial response (PR): at least a 30% decrease in the sum of diameters of target lesions, taking as reference the baseline sum of diameters

Progressive disease (PD): at least a 20% increase in the sum of diameters of target lesions, taking as reference the smallest sum on study (nadir), including baseline

- In addition to the relative increase of 20%, the sum must also demonstrate an absolute increase of at least 5 mm.
- The appearance of one or more new lesions is also considered progression.

"Use of this document is governed by the terms of use on the first page of this document."

Stable disease (SD): neither sufficient shrinkage to qualify for PR nor sufficient increase to qualify for PD, taking as reference the smallest sum on study

Special Notes on the Assessment of Target Lesions

Lymph Nodes. Lymph nodes identified as target lesions should always have the actual short axis measurement recorded (measured in the same anatomical plane as the baseline examination), even if the nodes regress to < 10 mm on study. This means that when lymph nodes are included as target lesions, the sum of lesions may not be zero even if CR criteria are met since a normal lymph node is defined as having a short axis < 10 mm.

Target Lesions That Become Too Small to Measure. While on study, all lesions (nodal and non-nodal) recorded at baseline should have their actual measurements recorded at each subsequent evaluation, even when very small (e.g., 2 mm). However, sometimes lesions or lymph nodes that are recorded as target lesions at baseline become so faint on the CT scan that the radiologist may not feel comfortable assigning an exact measure and may report them as being too small to measure. When this occurs, it is important that a value be recorded on the CRF as follows:

- If it is the opinion of the radiologist that the lesion has likely disappeared, the measurement should be recorded as 0 mm.
- If the lesion is believed to be present and is faintly seen but too small to measure, a default value of 5 mm should be assigned and below measurable limit (BML) should be ticked. (Note: It is less likely that this rule will be used for lymph nodes since they usually have a definable size when normal and are frequently surrounded by fat such as in the retroperitoneum; however, if a lymph node is believed to be present and is faintly seen but too small to measure, a default value of 5 mm should be assigned in this circumstance as well and BML should also be ticked.)

<u>To reiterate, however, if the radiologist is able to provide an actual measure, that should be recorded, even if it is below 5 mm, and, in that case, BML should not be ticked.</u>

Lesions That Split or Coalesce on Treatment. When non-nodal lesions fragment, the longest diameters of the fragmented portions should be added together to calculate the target lesion sum. Similarly, as lesions coalesce, a plane between them may be maintained that would aid in obtaining maximal diameter measurements of each individual lesion. If the lesions have truly coalesced such that they are no longer separable, the vector of the longest diameter in this instance should be the maximal longest diameter for the coalesced lesion.

"Use of this document is governed by the terms of use on the first page of this document."

Evaluation of Nontarget Lesions

This section provides the definitions of the criteria used to determine the tumor response for the group of nontarget lesions. While some nontarget lesions may actually be measurable, they need not be measured and, instead, should be assessed only qualitatively at the timepoints specified in the protocol.

CR: disappearance of all nontarget lesions and (if applicable) normalization of tumor marker level)

- All lymph nodes must be non-pathological in size (< 10 mm short axis).

Non-CR/Non-PD: persistence of one or more nontarget lesion(s) and/or (if applicable) maintenance of tumor marker level above the normal limits

PD: unequivocal progression of existing nontarget lesions

- The appearance of one or more new lesions is also considered progression.

Special Notes on Assessment of Progression of Nontarget Disease

When the Patient Also Has Measurable Disease. In this setting, to achieve unequivocal progression on the basis of the nontarget disease, there must be an overall level of substantial worsening in nontarget disease in a magnitude that, even in the presence of SD or PR in target disease, the overall tumor burden has increased sufficiently to merit discontinuation of therapy. A modest increase in the size of one or more nontarget lesions is usually not sufficient to qualify for unequivocal progression status. The designation of overall progression solely on the basis of change in nontarget disease in the face of SD or PR of target disease will therefore be extremely rare.

When the Patient Has Only Nonmeasurable Disease. This circumstance arises in some Phase III trials when it is not a criterion of study entry to have measurable disease. The same general concepts apply here as noted above; however, in this instance, there is no measurable disease assessment to factor into the interpretation of an increase in nonmeasurable disease burden. Because worsening in nontarget disease cannot be easily quantified (by definition: if all lesions are truly nonmeasurable), a useful test that can be applied when assessing patients for unequivocal progression is to consider if the increase in overall disease burden based on the change in nonmeasurable disease is comparable in magnitude to the increase that would be required to declare PD for measurable disease, that is, an increase in tumor burden representing an additional 73% increase in volume (which is equivalent to a 20% increase in diameter in a measurable lesion). Examples include an increase in a pleural effusion from “trace” to “large” or an increase in lymphangitic disease from localized to widespread or may be described in protocols as “sufficient to require a change in therapy.” If unequivocal progression is seen, the patient should be considered to have had overall PD at that

"Use of this document is governed by the terms of use on the first page of this document."

point. While it would be ideal to have objective criteria to apply to nonmeasurable disease, the very nature of that disease makes it impossible to do so; therefore, the increase must be substantial.

When the patient has bone lesions at baseline. When a bone scan is the sole indicator of progression, progression in bone will be defined as when at least two or more new lesions are seen on bone scan compared with screening. In situations where the scan findings are suggestive of a flare reaction, or apparent new lesion(s) which may represent trauma, these results must be confirmed with other imaging modalities such as MRI or fine-cut CT to constitute progression. Only a single new bone lesion on bone scan is required for progression if the lesion can be correlated on CT, MRI or plain film.

New Lesions

The appearance of new malignant lesions denotes disease progression; therefore, some comments on detection of new lesions are important. There are no specific criteria for the identification of new radiographic lesions; however, the finding of a new lesion should be unequivocal, that is, not attributable to differences in scanning technique, change in imaging modality, or findings thought to represent something other than tumor (for example, some "new" bone lesions may be simply healing or flare of preexisting lesions). This is particularly important when the patient's baseline lesions show partial or complete response. For example, necrosis of a liver lesion may be reported on a CT scan report as a "new" cystic lesion, which it is not.

A lesion identified during the study in an anatomical location that was not scanned at baseline is considered a new lesion and will indicate disease progression.

If a new lesion is equivocal, for example because of its small size, continued therapy and follow-up evaluation will clarify if it represents truly new disease. If repeat scans confirm there is definitely a new lesion, then progression should be declared using the date of the initial scan.

New osteoblastic bone lesions identified on plain films, CT, or MRI will not be considered progression in an otherwise stable or responding subject, if, in the opinion of the physician, the osteoblastic lesion appears to be healing or a response to therapy.

EVALUATION OF RESPONSE

Timepoint Response (Overall Response)

It is assumed that at each protocol-specified timepoint, a response assessment occurs. Table 1 provides a summary of the overall response status calculation at each timepoint for patients who have measurable disease at baseline.

"Use of this document is governed by the terms of use on the first page of this document."

When patients have nonmeasurable (therefore nontarget) disease only, Table 2 is to be used.

Table 1 Timepoint Response: Patients with Target Lesions (with or without Nontarget Lesions)

Target Lesions	Nontarget Lesions	New Lesions	Overall Response
CR	CR	No	CR
CR	Non-CR/non-PD	No	PR
CR	Not evaluated	No	PR
PR	Non-PD or not all evaluated	No	PR
SD	Non-PD or not all evaluated	No	SD
Not all evaluated	Non-PD	No	NE
PD	Any	Yes or no	PD
Any	PD	Yes or no	PD
Any	Any	Yes	PD

CR = complete response; NE = not evaluable; PD = progressive disease; PR = partial response; SD = stable disease.

Table 2 Timepoint Response: Patients with Nontarget Lesions Only

Nontarget Lesions	New Lesions	Overall Response
CR	No	CR
Non-CR/non-PD	No	Non-CR/non-PD [a]
Not all evaluated	No	NE
Unequivocal PD	Yes or no	PD
Any	Yes	PD

CR = complete response; NE = not evaluable; PD = progressive disease.

[a] "Non-CR/non-PD" is preferred over "stable disease" for nontarget disease since stable disease is increasingly used as an endpoint for assessment of efficacy in some trials; thus, assigning "stable disease" when no lesions can be measured is not advised.

Missing Assessments and Not-Evaluable Designation

When no imaging/measurement is done at all at a particular timepoint, the patient is not evaluable at that timepoint. If only a subset of lesion measurements are made at an assessment, usually the case is also considered not evaluable at that timepoint, unless a convincing argument can be made that the contribution of the individual missing lesion(s) would not change the assigned timepoint response. This would be most likely to happen

"Use of this document is governed by the terms of use on the first page of this document."

in the case of PD. For example, if a patient had a baseline sum of 50 mm with three measured lesions and, during the study, only two lesions were assessed, but those gave a sum of 80 mm, the patient will have achieved PD status, regardless of the contribution of the missing lesion.

If one or more target lesions were not assessed either because the scan was not done or the scan could not be assessed because of poor image quality or obstructed view, the response for target lesions should be “unable to assess” since the patient is not evaluable. Similarly, if one or more nontarget lesions are not assessed, the response for nontarget lesions should be “unable to assess” except where there is clear progression. Overall response would be “unable to assess” if either the target response or the nontarget response is “unable to assess,” except where this is clear evidence of progression as this equates with the case being not evaluable at that timepoint.

"Use of this document is governed by the terms of use on the first page of this document."

Table 3 Best Overall Response When Confirmation Is Required

Overall Response at First Timepoint	Overall Response at Subsequent Timepoint	Best Overall Response
CR	CR	CR
CR	PR	SD, PD, or PR [a]
CR	SD	SD, provided minimum duration for SD was met; otherwise, PD
CR	PD	SD, provided minimum duration for SD was met; otherwise, PD
CR	NE	SD, provided minimum duration for SD was met; otherwise, NE
PR	CR	PR
PR	PR	PR
PR	SD	SD
PR	PD	SD, provided minimum duration for SD was met; otherwise, PD
PR	NE	SD, provided minimum duration for SD was met; otherwise, NE
NE	NE	NE

CR = complete response; NE = not evaluable; PD = progressive disease; PR = partial response; SD = stable disease.

[a] If a CR is truly met at the first timepoint, any disease seen at a subsequent timepoint, even disease meeting PR criteria relative to baseline, qualifies as PD at that point (since disease must have reappeared after CR). Best response would depend on whether the minimum duration for SD was met. However, sometimes CR may be claimed when subsequent scans suggest small lesions were likely still present and in fact the patient had PR, not CR, at the first timepoint. Under these circumstances, the original CR should be changed to PR and the best response is PR.

Special Notes on Response Assessment

When nodal disease is included in the sum of target lesions and the nodes decrease to “normal” size (< 10 mm), they may still have a measurement reported on scans. This measurement should be recorded even though the nodes are normal in order not to overstate progression should it be based on increase in size of the nodes. As noted earlier, this means that patients with CR may not have a total sum of “zero” on the CRF.

Patients with a global deterioration of health status requiring discontinuation of treatment without objective evidence of disease progression at that time should be reported as “symptomatic deterioration.” Every effort should be made to document objective

"Use of this document is governed by the terms of use on the first page of this document."

progression even after discontinuation of treatment. Symptomatic deterioration is not a descriptor of an objective response; it is a reason for stopping study therapy. The objective response status of such patients is to be determined by evaluation of target and nontarget disease as shown in Table 1, Table 2, and Table 3.

For equivocal findings of progression (e.g., very small and uncertain new lesions; cystic changes or necrosis in existing lesions), treatment may continue until the next scheduled assessment. If at the next scheduled assessment progression is confirmed, the date of progression should be the earlier date when progression was suspected.

In studies for which patients with advanced disease are eligible (i.e., primary disease still or partially present), the primary tumor should also be captured as a target or nontarget lesion, as appropriate. This is to avoid an incorrect assessment of complete response if the primary tumor is still present but not evaluated as a target or nontarget lesion.

"Use of this document is governed by the terms of use on the first page of this document."

Appendix 5
Anaphylaxis Precautions

EQUIPMENT NEEDED

- Monitoring devices: ECG monitor, blood pressure monitor, oxygen saturation monitor, and thermometer
- Oxygen
- Epinephrine for subcutaneous, intravenous, and/or endotracheal use in accordance with standard practice
- Antihistamines
- Corticosteroids
- Intravenous infusion solutions, tubing, catheters, and tape

PROCEDURES

1. Stop the study drug infusion.
2. Call for additional medical assistance.
3. Maintain an adequate airway.
4. Ensure that appropriate monitoring is in place, with continuous ECG and pulse oximetry monitoring, if possible.
5. Administer antihistamines, epinephrine, or other medications as required by patient status and directed by the physician in charge.
6. Continue to observe the patient and document observations.

"Use of this document is governed by the terms of use on the first page of this document."

Appendix 6
Eastern Cooperative Oncology Group Performance Status Scale

Grade	Description
0	Fully active, able to carry on all predisease performance without restriction
1	Restricted in physically strenuous activity but ambulatory and able to carry out work of a light or sedentary nature, e.g., light housework or office work
2	Ambulatory and capable of all self-care but unable to carry out any work activities; up and about >50% of waking hours
3	Capable of only limited self-care, confined to a bed or chair >50% of waking hours
4	Completely disabled; cannot carry on any self-care; totally confined to bed or chair
5	Dead

"Use of this document is governed by the terms of use on the first page of this document."

Appendix 7
Preexisting Autoimmune Diseases

Subjects should be carefully questioned regarding their history of acquired or congenital immune deficiencies or autoimmune disease. Subjects with any history of immune deficiencies or autoimmune disease listed in the table below are excluded from participating in the study. Possible exceptions to this exclusion could be subjects with a medical history of such entities as atopic disease or childhood arthralgias where the clinical suspicion of autoimmune disease is low. Patients with a history of autoimmune-related hypothyroidism on a stable dose of thyroid replacement hormone may be eligible for this study. In addition, transient autoimmune manifestations of an acute infectious disease that resolved upon treatment of the infectious agent are not excluded (e.g., acute Lyme arthritis). Contact the Medical Monitor regarding any uncertainty over autoimmune exclusions.

Autoimmune Diseases and Immune Deficiencies

Acute disseminated encephalomyelitis	Dysautonomia	Ord's thyroiditis
Addison's disease	Epidermolysis bullosa acquista	Pemphigus
Ankylosing spondylitis	Gestational pemphigoid	Pernicious anemia
Antiphospholipid antibody syndrome	Giant cell arteritis	Polyarteritis nodusa
Aplastic anemia	Goodpasture's syndrome	Polyarthritis
Autoimmune hemolytic anemia	Graves' disease	Polyglandular autoimmune syndrome
Autoimmune hepatitis	Guillain-Barré syndrome	Primary biliary cirrhosis
Autoimmune hypoparathyroidism	Hashimoto's disease	Psoriasis
Autoimmune hypophysitis	IgA nephropathy	Reiter's syndrome
Autoimmune myocarditis	Inflammatory bowel disease	Rheumatoid arthritis
Autoimmune oophoritis	Interstitial cystitis	Sarcoidosis
Autoimmune orchitis	Kawasaki's disease	Scleroderma
Autoimmune thrombocytopenic purpura	Lambert-Eaton myasthenia syndrome	Sjögren's syndrome
Behcet's disease	Lupus erythematosus	Stiff-Person syndrome
Bullous pemphigold	Lyme disease - chronic	Takayasu's arteritis
Chronic fatigue syndrome	Meniere's syndrome	Ulcerative colitis
Chronic inflammatory demyelinating polyneuropathy	Mooren's ulcer	Vitiligo
Chung-Strauss syndrome	Morphea	Vogt-Kovanagi-Harada disease
Crohn's disease	Multiple sclerosis	Wegener's granulomatosis
Dermatomyositis	Myasthenia gravis	
Diabetes mellitus type 1	Neuromyotonia	
	Opsoclonus myoclonus syndrome	
	Optic neuritis	

"Use of this document is governed by the terms of use on the first page of this document."

Appendix 8
Management of Atezolizumab-Specific Adverse Events

Toxicities associated or possibly associated with atezolizumab treatment should be managed according to standard medical practice. Additional tests, such as autoimmune serology or biopsies, should be used to evaluate for a possible immunogenic etiology.

Although most immune-related adverse events observed with immunomodulatory agents have been mild and self-limiting, such events should be recognized early and treated promptly to avoid potential major complications. Discontinuation of atezolizumab may not have an immediate therapeutic effect, and in severe cases, immune-related toxicities may require acute management with topical corticosteroids, systemic corticosteroids, or other immunosuppressive agents.

The investigator should consider the benefit–risk balance a given patient may be experiencing prior to further administration of atezolizumab. In patients who have met the criteria for permanent discontinuation, resumption of atezolizumab may be considered if the patient is deriving benefit and has fully recovered from the immune-related event. Patients can be re-challenged with atezolizumab only after approval has been documented by both the investigator (or an appropriate delegate) and the Medical Monitor.

PULMONARY EVENTS

Dyspnea, cough, fatigue, hypoxia, pneumonitis, and pulmonary infiltrates have been associated with the administration of atezolizumab. Patients will be assessed for pulmonary signs and symptoms throughout the study and will also have computed tomography (CT) scans of the chest performed at every tumor assessment.

All pulmonary events should be thoroughly evaluated for other commonly reported etiologies such as pneumonia or other infection, lymphangitic carcinomatosis, pulmonary embolism, heart failure, chronic obstructive pulmonary disease, or pulmonary hypertension. Management guidelines for pulmonary events are provided in Table 1.

"Use of this document is governed by the terms of use on the first page of this document."

Table 1 Management Guidelines for Pulmonary Events, Including Pneumonitis

Event	Management
Pulmonary event, Grade 1	• Continue atezolizumab and monitor closely.<br>• Re-evaluate on serial imaging.<br>• Consider patient referral to pulmonary specialist.
Pulmonary event, Grade 2	• Withhold atezolizumab for up to 12 weeks after event onset. [a]<br>• Refer patient to pulmonary and infectious disease specialists and consider bronchoscopy or BAL.<br>• Initiate treatment with 1–2 mg/kg/day oral prednisone or equivalent.<br>• If event resolves to Grade 1 or better, resume atezolizumab. [b]<br>• If event does not resolve to Grade 1 or better while withholding atezolizumab, permanently discontinue atezolizumab and contact Medical Monitor. [c]<br>• For recurrent events, treat as a Grade 3 or 4 event.
Pulmonary event, Grade 3 or 4	• Permanently discontinue atezolizumab and contact Medical Monitor. [c]<br>• Bronchoscopy or BAL is recommended.<br>• Initiate treatment with 1–2 mg/kg/day oral prednisone or equivalent.<br>• If event does not improve within 48 hours after initiating corticosteroids, consider adding an immunosuppressive agent.<br>• If event resolves to Grade 1 or better, taper corticosteroids over $\geq$1 month.

BAL=bronchoscopic alveolar lavage.

[a] Atezolizumab may be withheld for a longer period of time (i.e., $>$12 weeks after event onset) to allow for corticosteroids (if initiated) to be reduced to $\leq$10 mg/day oral prednisone or equivalent. The acceptable length of the extended period of time must be agreed upon by the investigator and the Medical Monitor.

[b] If corticosteroids have been initiated, they must be tapered over $\geq$1 month to $\leq$10 mg/day oral prednisone or equivalent before atezolizumab can be resumed.

[c] Resumption of atezolizumab may be considered in patients who are deriving benefit and have fully recovered from the immune-related event. Patients can be re-challenged with atezolizumab only after approval has been documented by both the investigator (or an appropriate delegate) and the Medical Monitor.

"Use of this document is governed by the terms of use on the first page of this document."

HEPATIC EVENTS

Immune-related hepatitis has been associated with the administration of atezolizumab. Eligible patients must have adequate liver function, as manifested by measurements of total bilirubin and hepatic transaminases, and liver function will be monitored throughout study treatment. Management guidelines for hepatic events are provided in Table 2.

Patients with right upper-quadrant abdominal pain and/or unexplained nausea or vomiting should have liver function tests (LFTs) performed immediately and reviewed before administration of the next dose of study drug.

For patients with elevated LFTs, concurrent medication, viral hepatitis, and toxic or neoplastic etiologies should be considered and addressed, as appropriate.

Table 2 Management Guidelines for Hepatic Events

Event	Management
Hepatic event, Grade 1	• Continue atezolizumab.<br>• Monitor LFTs until values resolve to within normal limits.
Hepatic event, Grade 2	All events:<br>• Monitor LFTs more frequently until return to baseline values.<br>Events of > 5 days' duration:<br>• Withhold atezolizumab for up to 12 weeks after event onset. [a]<br>• Initiate treatment with 1–2 mg/kg/day oral prednisone or equivalent.<br>• If event resolves to Grade 1 or better, resume atezolizumab. [b]<br>• If event does not resolve to Grade 1 or better while withholding atezolizumab, permanently discontinue atezolizumab and contact Medical Monitor. [c]

LFT=liver function tests.

[a] Atezolizumab may be withheld for a longer period of time (i.e., > 12 weeks after event onset) to allow for corticosteroids (if initiated) to be reduced to ≤ 10 mg/day oral prednisone or equivalent. The acceptable length of the extended period of time must be agreed upon by the investigator and the Medical Monitor.

[b] If corticosteroids have been initiated, they must be tapered over ≥ 1 month to ≤ 10 mg/day oral prednisone or equivalent before atezolizumab can be resumed.

[c] Resumption of atezolizumab may be considered in patients who are deriving benefit and have fully recovered from the immune-related event. Patients can be re-challenged with atezolizumab only after approval has been documented by both the investigator (or an appropriate delegate) and the Medical Monitor.

"Use of this document is governed by the terms of use on the first page of this document."

Table 2 Management Guidelines for Hepatic Events (cont.)

Event	Management
Hepatic event, Grade 3 or 4	• Permanently discontinue atezolizumab and contact Medical Monitor. [c]<br>• Consider patient referral to gastrointestinal specialist for evaluation and liver biopsy to establish etiology of hepatic injury.<br>• Initiate treatment with 1–2 mg/kg/day oral prednisone or equivalent.<br>• If event does not improve within 48 hours after initiating corticosteroids, consider adding an immunosuppressive agent.<br>• If event resolves to Grade 1 or better, taper corticosteroids over ≥1 month.

LFT=liver function tests.

[a] Atezolizumab may be withheld for a longer period of time (i.e., >12 weeks after event onset) to allow for corticosteroids (if initiated) to be reduced to ≤10 mg/day oral prednisone or equivalent. The acceptable length of the extended period of time must be agreed upon by the investigator and the Medical Monitor.

[b] If corticosteroids have been initiated, they must be tapered over ≥1 month to ≤10 mg/day oral prednisone or equivalent before atezolizumab can be resumed.

[c] Resumption of atezolizumab may be considered in patients who are deriving benefit and have fully recovered from the immune-related event. Patients can be re-challenged with atezolizumab only after approval has been documented by both the investigator (or an appropriate delegate) and the Medical Monitor.

GASTROINTESTINAL EVENTS

Immune-related colitis has been associated with the administration of atezolizumab. Management guidelines for diarrhea or colitis are provided in Table 3.

All events of diarrhea or colitis should be thoroughly evaluated for other more common etiologies. For events of significant duration or magnitude or associated with signs of systemic inflammation or acute-phase reactants (e.g., increased C-reactive protein, platelet count, or bandemia): Perform sigmoidoscopy (or colonoscopy, if appropriate) with colonic biopsy, with three to five specimens for standard paraffin block to check for inflammation and lymphocytic infiltrates to confirm colitis diagnosis.

"Use of this document is governed by the terms of use on the first page of this document."

Table 3 Management Guidelines for Gastrointestinal Events (Diarrhea or Colitis)

Event	Management
Diarrhea or colitis, Grade 1	• Continue atezolizumab.<br>• Initiate symptomatic treatment.<br>• Endoscopy is recommended if symptoms persist for > 7 days.<br>• Monitor closely.
Diarrhea or colitis, Grade 2	• Withhold atezolizumab for up to 12 weeks after event onset. [a]<br>• Initiate symptomatic treatment.<br>• Patient referral to GI specialist is recommended.<br>• For recurrent events or events that persist ≥ 5 days, initiate treatment with 1–2 mg/kg/day oral prednisone or equivalent.<br>• If event resolves to Grade 1 or better, resume atezolizumab. [b]<br>• If event does not resolve to Grade 1 or better while withholding atezolizumab, permanently discontinue atezolizumab and contact Medical Monitor. [c]
Diarrhea or colitis, Grade 3	• Withhold atezolizumab for up to 12 weeks after event onset. [a]<br>• Refer patient to GI specialist for evaluation and confirmatory biopsy.<br>• Initiate treatment with 1–2 mg/kg/day IV methylprednisolone or equivalent and convert to 1–2 mg/kg/day oral prednisone or equivalent upon improvement.<br>• If event resolves to Grade 1 or better, resume atezolizumab. [b]<br>• If event does not resolve to Grade 1 or better while withholding atezolizumab, permanently discontinue atezolizumab and contact Medical Monitor. [c]

GI = gastrointestinal.

[a] Atezolizumab may be withheld for a longer period of time (i.e., > 12 weeks after event onset) to allow for corticosteroids (if initiated) to be reduced to ≤ 10 mg/day oral prednisone or equivalent. The acceptable length of the extended period of time must be agreed upon by the investigator and the Medical Monitor.

[b] If corticosteroids have been initiated, they must be tapered over ≥ 1 month to ≤ 10 mg/day oral prednisone or equivalent before atezolizumab can be resumed.

[c] Resumption of atezolizumab may be considered in patients who are deriving benefit and have fully recovered from the immune-related event. Patients can be re-challenged with atezolizumab only after approval has been documented by both the investigator (or an appropriate delegate) and the Medical Monitor.

"Use of this document is governed by the terms of use on the first page of this document."

Table 3 Management Guidelines for Gastrointestinal Events (Diarrhea or Colitis) (cont.)

Event	Management
Diarrhea or colitis, Grade 4	• Permanently discontinue atezolizumab and contact Medical Monitor. [c]<br>• Refer patient to GI specialist for evaluation and confirmation biopsy.<br>• Initiate treatment with 1–2 mg/kg/day IV methylprednisolone or equivalent and convert to 1–2 mg/kg/day oral prednisone or equivalent upon improvement.<br>• If event does not improve within 48 hours after initiating corticosteroids, consider adding an immunosuppressive agent.<br>• If event resolves to Grade 1 or better, taper corticosteroids over ≥1 month.

GI = gastrointestinal.

[a] Atezolizumab may be withheld for a longer period of time (i.e., > 12 weeks after event onset) to allow for corticosteroids (if initiated) to be reduced to ≤ 10 mg/day oral prednisone or equivalent. The acceptable length of the extended period of time must be agreed upon by the investigator and the Medical Monitor.

[b] If corticosteroids have been initiated, they must be tapered over ≥ 1 month to ≤ 10 mg/day oral prednisone or equivalent before atezolizumab can be resumed.

[c] Resumption of atezolizumab may be considered in patients who are deriving benefit and have fully recovered from the immune-related event. Patients can be re-challenged with atezolizumab only after approval has been documented by both the investigator (or an appropriate delegate) and the Medical Monitor.

ENDOCRINE EVENTS

Thyroid disorders, adrenal insufficiency, diabetes mellitus, and pituitary disorders have been associated with the administration of atezolizumab. Management guidelines for endocrine events are provided in Table 4.

Patients with unexplained symptoms such as headache, fatigue, myalgias, impotence, constipation, or mental status changes should be investigated for the presence of thyroid, pituitary, or adrenal endocrinopathies. The patient should be referred to an endocrinologist if an endocrinopathy is suspected. Thyroid-stimulating hormone (TSH) and free triiodothyronine and thyroxine levels should be measured to determine whether thyroid abnormalities are present. Pituitary hormone levels and function tests (e.g., TSH, growth hormone, luteinizing hormone, follicle-stimulating hormone, testosterone, prolactin, adrenocorticotropic hormone [ACTH] levels, and ACTH stimulation test) and magnetic resonance imaging (MRI) of the brain (with detailed pituitary sections) may help to differentiate primary pituitary insufficiency from primary adrenal insufficiency.

"Use of this document is governed by the terms of use on the first page of this document."

Table 4 Management Guidelines for Endocrine Events

Event	Management
Asymptomatic hypothyroidism	• Continue atezolizumab.<br>• Initiate treatment with thyroid replacement hormone.<br>• Monitor TSH weekly.
Symptomatic hypothyroidism	• Withhold atezolizumab.<br>• Initiate treatment with thyroid replacement hormone.<br>• Monitor TSH weekly.<br>• Consider patient referral to endocrinologist.<br>• Resume atezolizumab when symptoms are controlled and thyroid function is improving.
Asymptomatic hyperthyroidism	TSH $\geq$0.1 mU/L and $<$0.5 mU/L:<br>• Continue atezolizumab.<br>• Monitor TSH every 4 weeks.<br>TSH $<$0.1 mU/L:<br>• Follow guidelines for symptomatic hyperthyroidism.
Symptomatic hyperthyroidism	• Withhold atezolizumab.<br>• Initiate treatment with anti-thyroid drug such as methimazole or carbimazole as needed.<br>• Consider patient referral to endocrinologist.<br>• Resume atezolizumab when symptoms are controlled and thyroid function is improving.<br>• Permanently discontinue atezolizumab and contact Medical Monitor for life-threatening immune-related hyperthyroidism. [c]

MRI = magnetic resonance imaging; TSH = thyroid-stimulating hormone.

[a] Atezolizumab may be withheld for a longer period of time (i.e., > 12 weeks after event onset) to allow for corticosteroids (if initiated) to be reduced to ≤ 10 mg/day oral prednisone or equivalent. The acceptable length of the extended period of time must be agreed upon by the investigator and the Medical Monitor.

[b] If corticosteroids have been initiated, they must be tapered over ≥ 1 month to ≤ 10 mg/day oral prednisone or equivalent before atezolizumab can be resumed.

[c] Resumption of atezolizumab may be considered in patients who are deriving benefit and have fully recovered from the immune-related event. Patients can be re-challenged with atezolizumab only after approval has been documented by both the investigator (or an appropriate delegate) and the Medical Monitor.

"Use of this document is governed by the terms of use on the first page of this document."

Table 4 Management Guidelines for Endocrine Events (cont.)

Event	Management
Symptomatic adrenal insufficiency, Grade 2–4	• Withhold atezolizumab for up to 12 weeks after event onset. [a]<br>• Refer patient to endocrinologist.<br>• Perform appropriate imaging.<br>• Initiate treatment with 1–2 mg/kg/day IV methylprednisolone or equivalent and convert to 1–2 mg/kg/day oral prednisone or equivalent upon improvement.<br>• If event resolves to Grade 1 or better and patient is stable on replacement therapy, resume atezolizumab. [b]<br>• If event does not resolve to Grade 1 or better or patient is not stable on replacement therapy while withholding atezolizumab, permanently discontinue atezolizumab and contact Medical Monitor. [c]
Hyperglycemia, Grade 1 or 2	• Continue atezolizumab.<br>• Initiate treatment with insulin if needed.<br>• Monitor for glucose control.
Hyperglycemia, Grade 3 or 4	• Withhold atezolizumab.<br>• Initiate treatment with insulin.<br>• Monitor for glucose control.<br>• Resume atezolizumab when symptoms resolve and glucose levels are stable.

MRI = magnetic resonance imaging; TSH = thyroid-stimulating hormone.

[a] Atezolizumab may be withheld for a longer period of time (i.e., > 12 weeks after event onset) to allow for corticosteroids (if initiated) to be reduced to ≤ 10 mg/day oral prednisone or equivalent. The acceptable length of the extended period of time must be agreed upon by the investigator and the Medical Monitor.

[b] If corticosteroids have been initiated, they must be tapered over ≥ 1 month to ≤ 10 mg/day oral prednisone or equivalent before atezolizumab can be resumed.

[c] Resumption of atezolizumab may be considered in patients who are deriving benefit and have fully recovered from the immune-related event. Patients can be re-challenged with atezolizumab only after approval has been documented by both the investigator (or an appropriate delegate) and the Medical Monitor.

"Use of this document is governed by the terms of use on the first page of this document."

Table 4 Management Guidelines for Endocrine Events (cont.)

Event	Management
Hypophysitis (pan-hypopituitarism), Grade 2 or 3	• Withhold atezolizumab for up to 12 weeks after event onset. [a]<br>• Refer patient to endocrinologist.<br>• Perform brain MRI (pituitary protocol).<br>• Initiate treatment with 1–2 mg/kg/day IV methylprednisolone or equivalent and convert to 1–2 mg/kg/day oral prednisone or equivalent upon improvement.<br>• Initiate hormone replacement if clinically indicated.<br>• If event resolves to Grade 1 or better, resume atezolizumab. [b]<br>• If event does not resolve to Grade 1 or better while withholding atezolizumab, permanently discontinue atezolizumab and contact Medical Monitor. [c]<br>• For recurrent hypophysitis, treat as a Grade 4 event.
Hypophysitis (pan-hypopituitarism), Grade 4	• Permanently discontinue atezolizumab and contact Medical Monitor. [c]<br>• Refer patient to endocrinologist.<br>• Perform brain MRI (pituitary protocol).<br>• Initiate treatment with 1–2 mg/kg/day IV methylprednisolone or equivalent and convert to 1–2 mg/kg/day oral prednisone or equivalent upon improvement.<br>• Initiate hormone replacement if clinically indicated.

MRI = magnetic resonance imaging; TSH = thyroid-stimulating hormone.

[a] Atezolizumab may be withheld for a longer period of time (i.e., > 12 weeks after event onset) to allow for corticosteroids (if initiated) to be reduced to ≤ 10 mg/day oral prednisone or equivalent. The acceptable length of the extended period of time must be agreed upon by the investigator and the Medical Monitor.

[b] If corticosteroids have been initiated, they must be tapered over ≥ 1 month to ≤ 10 mg/day oral prednisone or equivalent before atezolizumab can be resumed.

[c] Resumption of atezolizumab may be considered in patients who are deriving benefit and have fully recovered from the immune-related event. Patients can be re-challenged with atezolizumab only after approval has been documented by both the investigator (or an appropriate delegate) and the Medical Monitor.

OCULAR EVENTS

An ophthalmologist should evaluate visual complaints (e.g., uveitis, retinal events). Management guidelines for ocular events are provided in Table 5.

"Use of this document is governed by the terms of use on the first page of this document."

Table 5 Management Guidelines for Ocular Events

Event	Management
Ocular event, Grade 1	• Continue atezolizumab.<br>• Patient referral to ophthalmologist is strongly recommended.<br>• Initiate treatment with topical corticosteroid eye drops and topical immunosuppressive therapy.<br>• If symptoms persist, treat as a Grade 2 event.
Ocular event, Grade 2	• Withhold atezolizumab for up to 12 weeks after event onset. [a]<br>• Patient referral to ophthalmologist is strongly recommended.<br>• Initiate treatment with topical corticosteroid eye drops and topical immunosuppressive therapy.<br>• If event resolves to Grade 1 or better, resume atezolizumab. [b]<br>• If event does not resolve to Grade 1 or better while withholding atezolizumab, permanently discontinue atezolizumab and contact Medical Monitor. [c]
Ocular event, Grade 3 or 4	• Permanently discontinue atezolizumab and contact Medical Monitor. [c]<br>• Refer patient to ophthalmologist.<br>• Initiate treatment with 1–2 mg/kg/day oral prednisone or equivalent.<br>• If event resolves to Grade 1 or better, taper corticosteroids over ≥1 month.

[a] Atezolizumab may be withheld for a longer period of time (i.e., > 12 weeks after event onset) to allow for corticosteroids (if initiated) to be reduced to ≤10 mg/day oral prednisone or equivalent. The acceptable length of the extended period of time must be agreed upon by the investigator and the Medical Monitor.

[b] If corticosteroids have been initiated, they must be tapered over ≥1 month to ≤10 mg/day oral prednisone or equivalent before atezolizumab can be resumed.

[c] Resumption of atezolizumab may be considered in patients who are deriving benefit and have fully recovered from the immune-related event. Patients can be re-challenged with atezolizumab only after approval has been documented by both the investigator (or an appropriate delegate) and the Medical Monitor.

"Use of this document is governed by the terms of use on the first page of this document."

IMMUNE-RELATED MYOCARDITIS

Immune-related myocarditis has been associated with the administration of atezolizumab. Immune-related myocarditis should be suspected in any patient presenting with signs or symptoms suggestive of myocarditis, including, but not limited to, dyspnea, chest pain, palpitations, fatigue, decreased exercise tolerance, or syncope. Immune-related myocarditis needs to be distinguished from myocarditis resulting from infection (commonly viral, e.g., in a patient who reports a recent history of GI illness), ischemic events, underlying arrhythmias, exacerbation of preexisting cardiac conditions, or progression of malignancy.

All patients with possible myocarditis should be urgently evaluated by performing cardiac enzyme assessment, an ECG, a chest X-ray, an echocardiogram, and a cardiac MRI as appropriate per institutional guidelines. A cardiologist should be consulted. An endomyocardial biopsy may be considered to enable a definitive diagnosis and appropriate treatment, if clinically indicated.

Patients with signs and symptoms of myocarditis, in the absence of an identified alternate etiology, should be treated according to the guidelines in Table 6.

"Use of this document is governed by the terms of use on the first page of this document."

Table 6 Management Guidelines for Immune-Related Myocarditis

Event	Management
Immune-related myocarditis, Grade 1	• Refer patient to cardiologist.<br>• Initiate treatment as per institutional guidelines.
Immune-related myocarditis, Grade 2	• Withhold atezolizumab for up to 12 weeks after event onset [a] and contact Medical Monitor.<br>• Refer patient to cardiologist.<br>• Initiate treatment as per institutional guidelines and consider antiarrhythmic drugs, temporary pacemaker, ECMO, or VAD as appropriate.<br>• Consider treatment with 1–2 mg/kg/day IV methylprednisolone or equivalent and convert to 1–2 mg/kg/day oral prednisone or equivalent upon improvement.[a]<br>• If event resolves to Grade 1 or better, resume atezolizumab. [b]<br>• If event does not resolve to Grade 1 or better while withholding atezolizumab, permanently discontinue atezolizumab and contact Medical Monitor. [c]
Immune-related myocarditis, Grade 3-4	• Permanently discontinue atezolizumab and contact Medical Monitor. [c]<br>• Refer patient to cardiologist.<br>• Initiate treatment as per institutional guidelines and consider antiarrhythmic drugs, temporary pacemaker, ECMO, or VAD as appropriate.<br>• Initiate treatment with 1–2 mg/kg/day IV methylprednisolone or equivalent and convert to 1–2 mg/kg/day oral prednisone or equivalent upon improvement. [a,b]<br>• If event does not improve within 48 hours after initiating corticosteroids, consider adding an immunosuppressive agent.<br>• If event resolves to Grade 1 or better, taper corticosteroids over ≥ 1 month.

ECMO = extracorporeal membrane oxygenation; VAD = ventricular assist device.

[a] Atezolizumab may be withheld for a longer period of time (i.e., > 12 weeks after event onset) to allow for corticosteroids (if initiated) to be reduced to ≤ 10 mg/day oral prednisone or equivalent. The acceptable length of the extended period of time must be agreed upon by the investigator and the Medical Monitor.

[b] If corticosteroids have been initiated, they must be tapered over ≥ 1 month to ≤ 10 mg/day oral prednisone or equivalent before atezolizumab can be resumed.

[c] Resumption of atezolizumab may be considered in patients who are deriving benefit and have fully recovered from the immune-related event. Patients can be re-challenged with atezolizumab only after approval has been documented by both the investigator (or an appropriate delegate) and the Medical Monitor.

"Use of this document is governed by the terms of use on the first page of this document."

INFUSION-RELATED REACTIONS

No premedication is indicated for the administration of Cycle 1 of atezolizumab. However, patients who experience an infusion-related reaction (IRR) with Cycle 1 of atezolizumab may receive premedication with antihistamines or antipyretics/analgesics (e.g., acetaminophen) for subsequent infusions. Metamizole (dipyrone) is prohibited in treating atezolizumab-associated IRRs because of its potential for causing agranulocytosis.

Guidelines for medical management of IRRs during Cycle 1 are provided in Table 7. For subsequent cycles, IRRs should be managed according to institutional guidelines.

Table 7 Management Guidelines for Infusion-Related Reactions

Event	Management
IRR, Grade 1	• Reduce infusion rate to half the rate being given at the time of event onset.<br>• After the event has resolved, the investigator should wait for 30 minutes while delivering the infusion at the reduced rate.<br>• If the infusion is tolerated at the reduced rate for 30 minutes after symptoms have resolved, the infusion rate may be increased to the original rate.
IRR, Grade 2	• Interrupt atezolizumab infusion.<br>• Administer aggressive symptomatic treatment (e.g., oral or IV antihistamine, anti-pyretic medication, glucocorticoids, epinephrine, bronchodilators, oxygen).<br>• After symptoms have resolved to baseline, resume infusion at half the rate being given at the time of event onset.<br>• For subsequent infusions, consider administration of oral premedication with antihistamines, anti-pyretics, and/or analgesics and monitor closely for IRRs.
IRR, Grade 3 or 4	• Stop infusion.<br>• Administer aggressive symptomatic treatment (e.g., oral or IV antihistamine, anti-pyretic, glucocorticoids, epinephrine, bronchodilators, oxygen).<br>• Permanently discontinue atezolizumab and contact Medical Monitor. [a]

IRR=infusion-related reaction.

[a] Resumption of atezolizumab may be considered in patients who are deriving benefit and have fully recovered from the immune-related event. Patients can be re-challenged with atezolizumab only after approval has been documented by both the investigator (or an appropriate delegate) and the Medical Monitor.

"Use of this document is governed by the terms of use on the first page of this document."

PANCREATIC EVENTS

Symptoms of abdominal pain associated with elevations of amylase and lipase, suggestive of pancreatitis, have been associated with the administration of atezolizumab. The differential diagnosis of acute abdominal pain should include pancreatitis. Appropriate work-up should include an evaluation for ductal obstruction, as well as serum amylase and lipase tests. Management guidelines for pancreatic events, including pancreatitis, are provided in Table 8.

Table 8 Management Guidelines for Pancreatic Events, Including Pancreatitis

Event	Management
Amylase and/or lipase elevation, Grade 2	• Continue atezolizumab.<br>• Monitor amylase and lipase weekly.<br>• For prolonged elevation (e.g., > 3 weeks), consider treatment with 10 mg/day oral prednisone or equivalent.
Amylase and/or lipase elevation, Grade 3 or 4	• Withhold atezolizumab for up to 12 weeks after event onset. [a]<br>• Refer patient to GI specialist.<br>• Monitor amylase and lipase every other day.<br>• If no improvement, consider treatment with 1–2 mg/kg/day oral prednisone or equivalent.<br>• If event resolves to Grade 1 or better, resume atezolizumab. [b]<br>• If event does not resolve to Grade 1 or better while withholding atezolizumab, permanently discontinue atezolizumab and contact Medical Monitor. [c]<br>• For recurrent events, permanently discontinue atezolizumab and contact Medical Monitor. [c]

GI = gastrointestinal.

[a] Atezolizumab may be withheld for a longer period of time (i.e., > 12 weeks after event onset) to allow for corticosteroids (if initiated) to be reduced to ≤ 10 mg/day oral prednisone or equivalent. The acceptable length of the extended period of time must be agreed upon by the investigator and the Medical Monitor.

[b] If corticosteroids have been initiated, they must be tapered over ≥ 1 month to ≤ 10 mg/day oral prednisone or equivalent before atezolizumab can be resumed.

[c] Resumption of atezolizumab may be considered in patients who are deriving benefit and have fully recovered from the immune-related event. Patients can be re-challenged with atezolizumab only after approval has been documented by both the investigator (or an appropriate delegate) and the Medical Monitor.

"Use of this document is governed by the terms of use on the first page of this document."

Table 8 Management Guidelines for Pancreatic Events, Including Pancreatitis (cont.)

Event	Management
Immune-related pancreatitis, Grade 2 or 3	• Withhold atezolizumab for up to 12 weeks after event onset. [a]<br>• Refer patient to GI specialist.<br>• Initiate treatment with 1–2 mg/kg/day IV methylprednisolone or equivalent and convert to 1–2 mg/kg/day oral prednisone or equivalent upon improvement.<br>• If event resolves to Grade 1 or better, resume atezolizumab. [b]<br>• If event does not resolve to Grade 1 or better while withholding atezolizumab, permanently discontinue atezolizumab and contact Medical Monitor. [c]<br>• For recurrent events, permanently discontinue atezolizumab and contact Medical Monitor. [c]
Immune-related pancreatitis, Grade 4	• Permanently discontinue atezolizumab and contact Medical Monitor. [c]<br>• Refer patient to GI specialist.<br>• Initiate treatment with 1–2 mg/kg/day IV methylprednisolone or equivalent and convert to 1–2 mg/kg/day oral prednisone or equivalent upon improvement.<br>• If event does not improve within 48 hours after initiating corticosteroids, consider adding an immunosuppressive agent.<br>• If event resolves to Grade 1 or better, taper corticosteroids over ≥ 1 month.

GI = gastrointestinal.

[a] Atezolizumab may be withheld for a longer period of time (i.e., > 12 weeks after event onset) to allow for corticosteroids (if initiated) to be reduced to ≤ 10 mg/day oral prednisone or equivalent. The acceptable length of the extended period of time must be agreed upon by the investigator and the Medical Monitor.

[b] If corticosteroids have been initiated, they must be tapered over ≥ 1 month to ≤ 10 mg/day oral prednisone or equivalent before atezolizumab can be resumed.

[c] Resumption of atezolizumab may be considered in patients who are deriving benefit and have fully recovered from the immune-related event. Patients can be re-challenged with atezolizumab only after approval has been documented by both the investigator (or an appropriate delegate) and the Medical Monitor.

DERMATOLOGIC EVENTS

Treatment-emergent rash has been associated with atezolizumab. The majority of cases of rash were mild in severity and self-limited, with or without pruritus. A dermatologist should evaluate persistent and/or severe rash or pruritus. A biopsy should

"Use of this document is governed by the terms of use on the first page of this document."

be considered unless contraindicated. Management guidelines for dermatologic events are provided in Table 9.

Table 9 Management Guidelines for Dermatologic Events

Event	Management
Dermatologic event, Grade 1	• Continue atezolizumab.<br>• Consider treatment with topical corticosteroids and/or other symptomatic therapy (e.g., antihistamines).
Dermatologic event, Grade 2	• Continue atezolizumab.<br>• Consider patient referral to dermatologist.<br>• Initiate treatment with topical corticosteroids.<br>• Consider treatment with higher-potency topical corticosteroids if event does not improve.
Dermatologic event, Grade 3	• Withhold atezolizumab for up to 12 weeks after event onset. [a]<br>• Refer patient to dermatologist.<br>• Initiate treatment with 10 mg/day oral prednisone or equivalent, increasing dose to 1–2 mg/kg/day if event does not improve within 48–72 hours.<br>• If event resolves to Grade 1 or better, resume atezolizumab. [b]<br>• If event does not resolve to Grade 1 or better while withholding atezolizumab, permanently discontinue atezolizumab and contact Medical Monitor. [c]
Dermatologic event, Grade 4	• Permanently discontinue atezolizumab and contact Medical Monitor. [c]

[a] Atezolizumab may be withheld for a longer period of time (i.e., > 12 weeks after event onset) to allow for corticosteroids (if initiated) to be reduced to ≤ 10 mg/day oral prednisone or equivalent. The acceptable length of the extended period of time must be agreed upon by the investigator and the Medical Monitor.

[b] If corticosteroids have been initiated, they must be tapered over ≥ 1 month to ≤ 10 mg/day oral prednisone or equivalent before atezolizumab can be resumed.

[c] Resumption of atezolizumab may be considered in patients who are deriving benefit and have fully recovered from the immune-related event. Patients can be re-challenged with atezolizumab only after approval has been documented by both the investigator (or an appropriate delegate) and the Medical Monitor.

NEUROLOGIC DISORDERS

Myasthenia gravis and Guillain-Barré syndrome have been observed with single-agent atezolizumab. Patients may present with signs and symptoms of sensory and/or motor neuropathy. Diagnostic work-up is essential for an accurate characterization to differentiate between alternative etiologies. Management guidelines for neurologic disorders are provided in Table 10.

"Use of this document is governed by the terms of use on the first page of this document."

Table 10 Management Guidelines for Neurologic Disorders

Event	Management
Immune-related neuropathy, Grade 1	• Continue atezolizumab.<br>• Investigate etiology.
Immune-related neuropathy, Grade 2	• Withhold atezolizumab for up to 12 weeks after event onset. [a]<br>• Investigate etiology.<br>• Initiate treatment as per institutional guidelines.<br>• If event resolves to Grade 1 or better, resume atezolizumab. [b]<br>• If event does not resolve to Grade 1 or better while withholding atezolizumab, permanently discontinue atezolizumab and contact Medical Monitor. [c]
Immune-related neuropathy, Grade 3 or 4	• Permanently discontinue atezolizumab and contact Medical Monitor. [c]<br>• Initiate treatment as per institutional guidelines.
Myasthenia gravis and Guillain-Barré syndrome (any grade)	• Permanently discontinue atezolizumab and contact Medical Monitor. [c]<br>• Refer patient to neurologist.<br>• Initiate treatment as per institutional guidelines.<br>• Consider initiation of 1–2 mg/kg/day oral or IV prednisone or equivalent.

[a] Atezolizumab may be withheld for a longer period of time (i.e., > 12 weeks after event onset) to allow for corticosteroids (if initiated) to be reduced to ≤ 10 mg/day oral prednisone or equivalent. The acceptable length of the extended period of time must be agreed upon by the investigator and the Medical Monitor.

[b] If corticosteroids have been initiated, they must be tapered over ≥ 1 month to ≤ 10 mg/day oral prednisone or equivalent before atezolizumab can be resumed.

[c] Resumption of atezolizumab may be considered in patients who are deriving benefit and have fully recovered from the immune-related event. Patients can be re-challenged with atezolizumab only after approval has been documented by both the investigator (or an appropriate delegate) and the Medical Monitor.

IMMUNE-RELATED MENINGOENCEPHALITIS

Immune-related meningoencephalitis is an identified risk associated with the administration of atezolizumab. Immune-related meningoencephalitis should be suspected in any patient presenting with signs or symptoms suggestive of meningitis or encephalitis, including, but not limited to, headache, neck pain, confusion, seizure, motor or sensory dysfunction, and altered or depressed level of consciousness. Encephalopathy from metabolic or electrolyte imbalances needs to be distinguished from potential meningoencephalitis resulting from infection (bacterial, viral, or fungal) or progression of malignancy, or secondary to a paraneoplastic process.

"Use of this document is governed by the terms of use on the first page of this document."

All patients being considered for meningoencephalitis should be urgently evaluated with a CT scan and/or MRI scan of the brain to evaluate for metastasis, inflammation, or edema. If deemed safe by the treating physician, a lumbar puncture should be performed and a neurologist should be consulted.

Patients with signs and symptoms of meningoencephalitis, in the absence of an identified alternate etiology, should be treated according to the guidelines in Table 11.

Table 11 Management Guidelines for Immune-Related Meningoencephalitis

Event	Management
Immune-related meningoencephalitis, all grades	• Permanently discontinue atezolizumab and contact Medical Monitor. [a]<br>• Refer patient to neurologist.<br>• Initiate treatment with 1–2 mg/kg/day IV methylprednisolone or equivalent and convert to 1–2 mg/kg/day oral prednisone or equivalent upon improvement.<br>• If event does not improve within 48 hours after initiating corticosteroids, consider adding an immunosuppressive agent.<br>• If event resolves to Grade 1 or better, taper corticosteroids over ≥ 1 month.

[a] Resumption of atezolizumab may be considered in patients who are deriving benefit and have fully recovered from the immune-related event. Patients can be re-challenged with atezolizumab only after approval has been documented by both the investigator (or an appropriate delegate) and the Medical Monitor.

"Use of this document is governed by the terms of use on the first page of this document."
